# Supplementary material for: A low molecular weight dextran sulphate, ILB®, for the treatment of amyotrophic lateral sclerosis (ALS): An open-label, single-arm, single-centre, phase II trial
Source: PLoS One. 2024 Jul 11;19(7):e0291285. doi: 10.1371/journal.pone.0291285 (PMC11239073; doi:10.1371/journal.pone.0291285)
Supplement: S1 Appendix — A sample version of the finalised trial protocol (Version 8.0) dated 25-Nov-2020. (PDF) [file pone.0291285.s001.pdf]

Supplementary appendix 1: Sample version of the ALS trial protocol

A Phase II pilot safety and tolerability study of ILB in patients with Motor Neurone Disease (MND)/ Amyotrophic Lateral Sclerosis (ALS)

Trial Protocol Version	8.0
Date	25-Nov-2020
Sponsor	University of Birmingham
EudraCT Number	2018-000668-28
ISRCTN	TBC
Clinicaltrials.gov identifier	NCT03705390
Sponsor Protocol Number	RG_17-250
CRCTU Reference	XX2007

SAMPLE

Study Management Group

**Chief Investigator**	Dr Venkataramanan Srinivasan<br>[redacted]
**Co-Investigators**	Mr Simon Bach<br>[redacted]<br><br>[redacted]<br><br>Kristian Brock<br>[redacted]
**Lead Clinical Pharmacist**	[redacted]
**Clinical Coordinator**	[redacted]<br><br>[redacted]
**TikoMed Medical Expert**	[redacted]
**Translational Collaborators**	Dr Zsuzsanna Nagy<br>[redacted]
**Trial Coordinator**	Siân Jenkins<br>[redacted]
**Senior Trial Coordinator**	Abigail Clutterbuck-James<br>[redacted]
**Trial Biostatistician**	Victoria Homer<br>[redacted]
**Senior Trial Biostatistician**	[redacted]

SAMPLE

Trial Management Team Leader Mr Darren Barton
[redacted]

ALS Trial Office

D^{3}B – Diagnostics Drugs Devices & Biomarkers, CRCTU
Heritage Building
5th Floor East, ITM,
Mindelsohn Way,
Edgbaston,
Birmingham,
B15 2TH

Enquires
☎ +44(0) 121 371 8492
✉ ALS@trials.bham.ac.uk

Registration
www.cancertrials.bham.ac.uk

Emergency Registration
☎+44(0) 121 371 8468

Serious Adverse Event Reporting
✉ reg@trials.bham.ac.uk
☎+ 44(0) 121 371 8492

SAMPLE

SPONSOR

The University of Birmingham, Edgbaston, Birmingham, B15 2TT

SIGNATURE PAGE

ALS Trial Protocol v8.0_25 Nov 2020

This protocol has been approved by:

**Name:**	Dr Venkataramanan Srinivasan	**Trial Role:**	Chief Investigator
**Signature:**	______________________	**Date:**	DD / MON / YYYY

SAMPLE

This protocol describes the ALS trial and provides information about procedures for patients taking part in the ALS trial. The protocol should not be used as a guide for treatment of patients not taking part in the ALS trial.

This Protocol was written using CRCTU-PRT-QCD-002 Version 1.0

AMENDMENTS

The following amendments and/or administrative changes have been made to this protocol since the implementation of the first approved version

Amendment number	Date of amendment	Protocol version number	Type of amendment	Summary of amendment
1	31-May-2018	2.0	Substantial	Amendments to Data Protection (Section 15) to incorporate the General Data Protection Regulation (GDPR) 2018
2	11-June-2018	3.0	Not applicable	Minor amendments following internal review by Research Governance
3	20-Jul-2018	4.0	Substantial	Amendments to Section 1.2.3 (rationale for dose), Schedule of assessments to include a week 26 visit, contraception advice clarified, amendments to RSI and AE reporting as per MHRA initial assessment of CTA request
4	04-Apr-2019	5.0	Substantial	Amendment to 1.1.5. Update to background of ILB in line with Investigator Brochure V3.0<br><br>Schedule of assessments, Section 2.0, Section 7.4 and Appendix 12 have been amended to add PBMC sample collection to assess phenotypic profiling.<br><br>Section 4.0 eligibility criteria has been amended to update the lung function assessments inclusion criteria (inclusion criteria 4), changes to the homeostasis markers (inclusion criteria 6) and clarification to exclusion criteria 10.<br><br>Amendment to schedule of assessments, section 7.3.1 Myoglobin has been removed from the biochemistry sample analysis

SAMPLE

5		6.0	Substantial	**Clinical Coordinator**<br>Clinical coordinator added<br>**2.1 Primary Objective**<br>Time frame for objective has been removed<br>**3.0 Trial Design**<br>**4.2 Eligibility Criteria**<br>Eligibility criteria to continue onto the treatment extension has been added.<br>**Trial Duration**<br>Trial duration has been amended depending on if patients continue onto treatment extension<br>**Trial Schema**<br>Updated based on treatment extension<br>**Schedule of Assessments**<br>Pregnancy testing to be completed at screening and prior to Day 1 treatment<br>Additional schedule added for patients who go onto the treatment extension after 10 weeks.<br>**5.1 Consent**<br>Patient process to re-consent to treatment extension<br>**6.1.3 Treatment Continuation Registration**<br>**7.2 Treatment Schedule**<br>**7.6 Treatment Interruptions**<br>'Maximum of 2 weeks without treatment' has been removed. This is reviewed at clinicians discretion

SAMPLE

6	16-Jan-2020	7.0	Substantial	**Schedule of assessments**<br>Option to extend treatment to a maximum of up to 48 weeks based on clinical assessments and a discussion between the patient and clinician.<br><br>Week 11 lung function tests have been moved to week 9.<br><br>Week 11 intensive visit moved to week 10.<br><br>**Trial Schema**<br>Updated to reflect the possible further treatment period of up to a maximum of 48 weeks.<br><br>**Clinical assessments**<br>Thyroid and troponin tests have been removed from assessments post week 24 in order to reduce treatment costs<br><br>**Dynamometer test**<br>Has been introduced into the study in order to assess the muscle strength using a hand-held device.

SAMPLE

7		8.0	Substantial	**Trial Synopsis**<br>Updated to include single point long-term remote follow up visit starting in Q1 2021; Amended secondary outcome measures and exploratory outcome measures; list of biomarkers to be analysed; Updated trial duration<br><br>**Schedule of assessments**<br>Updated to include single point long-term remote follow up visit and collection of ALSFRS-R. ALSAQ-40, concomitant and current medication<br><br>**Trial Schema**<br>Updated to include single point long-term remote follow up visit<br><br>**2.4 Secondary Outcome Measures**<br>Updated to include PK analysis<br><br>**2.5 Exploratory Outcome Measures**<br>List of all biomarkers to be analysed<br><br>**3. Trial Design**<br>Updated to include single point long-term remote follow up visit<br><br>**5.1 Informed Consent**<br>5.1.2 Added for electronic re-consent for the single point long-term remote follow up visit<br><br>**7.3.5 Quality of life**<br>Updated to include the collection of QoL data in the form of ALSAQ-40 in single point long-term remote follow up visit<br><br>**7.4 Sample collection**<br>Amendments throughout to

SAMPLE

				reflect planned analysis, sample storage and laboratories involved<br><br>**7.14 Patient Follow Up**<br>Updated to include single point follow-up visit<br><br>**8.2.1.2 Serious Adverse Events**<br>Updated to reflect e-mail reporting procedure.<br><br>**11. End of Trial Definition**<br>Amended to coincide with updated timelines; final summary of clinical trial report and addendum including data from single point long-term remote follow up visits<br>**Non-substantial changes**<br><br>1. Changes to trial management personal / trial management group<br>2. All references to saliva sample collection and analysis removed as per previous amendment<br>**3.** Removal of fax information for SAE reporting in line with updated processes<br>**4.** Reformatted reference library to link to correct references, amending a formatting error in previous versions

SAMPLE

TRIAL SYNOPSIS

Trial Title

A Phase II pilot single-arm safety and tolerability study of ILB in patients with Motor Neurone Disease (MND)/ Amyotrophic Lateral Sclerosis (ALS)

Trial Design

This is a phase IIa, pilot, single arm (uncontrolled) study to assess the safety and tolerability of ILB

Objectives

Primary:

- To determine the safety and tolerability of ILB administered subcutaneously weekly in ALS patients

Secondary:

To describe the effect of ILB administered subcutaneously weekly

  - upon the severity of symptoms associated with ALS present at entry into the study
  - upon development of new symptoms associated with ALS during the study
  - upon patients' quality of life during the study

- To assess changes in biomarkers, including urinary p75[ECD] and plasma NfL.

Outcome Measures

Primary

Our primary outcome measures will inform whether the therapy is feasible in this patient group.

- **Safety**
  - Measured by the incidence of serious adverse events (SAEs) and adverse events (AEs) using CTCAE v4.0. Events will be summarised by grade, relatedness, admitting event (for SAEs); expectedness and sequelae.
- **Tolerability**
  - Measured by the incidence of *intolerable adverse events*. An intolerable adverse event will satisfy all of the following criteria:
    1. Associated with a serious adverse event or a drug discontinuation of greater than three weeks;
    2. Grade 3, 4 or 5 in severity according to CTCAE version 4;
    3. In the opinion of the Investigator is i) definitely related or ii) probably related or iii) possibly related to the study drug treatment.
  - Adverse events which are considered unrelated or probably not related will not be classed as intolerable events.
- **Quantity of study drug administered**
  - Total drug administered, number of administrations, number and length of interruptions and number of discontinuations will be reported.

Secondary

Our secondary outcome measures will provide data on patient function and disease progression.

- Revised ALS Functional Rating Scale (ALSFRS-R)
  - This is a functional rating scale, including assessments of communication, mobility, feeding, dressing and respiration.
- ALS Assessment Questionnaire (ALSAQ-40)

SAMPLE

  - This patient-reported outcome measures the subjective well-being of patients. It is broader than ALSFRS-R and adds assessment of emotional reactions.
- Urinary p75^{ECD}
  - This is a biological fluid-based biomarker of ALS disease progression.
- PK ILB in plasma following administration
  - This quantifies the amount of drug detectable in the blood after administration over time
- NfL in plasma
  - This is a blood-based biomarker for neurodegeneration.

Exploratory:

- HPLC analyses of purine-pyrimidine metabolites (serum)
- HPLC analysis of fat-soluble vitamins and antioxidants (serum)
- HPLC analyses of amino acids (AA) and amino-group containing compounds (ACCG) (serum)
- Spectrophotometric analysis of lactate
  - Blood samples will be collected for analysis of change in PBMC phenotypic balance
  - HGF release after ILB administration (ELISA from PK samples)
  - Creatine kinase and Myoglobin from plasma as markers of muscle atrophy associated with ALS

Patient Population

Patients with a diagnosis of ALS

Sample Size

This study seeks to recruit 15 patients

For the purposes of safety, it is proposed that the first 2 patients will be recruited as Sentinel patients. These will be recruited in series and each will be assessed for 2 weeks before the next patient will be recruited. Data on Sentinel patients will be assessed by an independent safety monitoring committee. If the safety monitoring committee is satisfied that the product has an acceptable safety profile in the sentinel patients, the study will be opened to general recruitment.

For a patient to be evaluable completion of at least one treatment is required.

Main Eligibility Criteria

Inclusion Criteria:

1. Patients ≥18 years and who have provided written informed consent to participate in the study
2. Prior to trial entry patients will have a *definite* diagnosis of ALS according to El Escorial Criteria (Appendix 10). All patients will demonstrate either:
   - **presence of Upper Motor Neuron (UMN)** (increased tone, brisk reflexes) **as well as Lower Motor Neuron (LMN)** (weakness, wasting and fasciculation) **signs in the bulbar region and at least two of the other spinal regions** (cervical, thoracic or lumbosacral)

     or

   - **presence of UMN and LMN signs in all three spinal regions** (cervical, thoracic or lumbosacral)

SAMPLE

3. Electrophysiological tests (Electromyography (EMG) / Nerve Conduction Study (NCS)) that supports the diagnosis of Motor Neurone Disease (MND) including; i) reduced recruitment (reduced interference pattern with firing rates over 10 Hz), ii) Large motor unit action potentials (large amplitude, long duration), and Fibrillation potentials.
4. Forced Vital Capacity (FVC) ≥50% of predicted value for gender, height and age at screening and/or a mean Sniff Nasal Inspiratory Pressure (SNIP) ≥50% of predicted value for age
5. Adequate haematological function (Hb≥10g/dl, absolute neutrophil count ≥1.5x10^9/L and a platelet count ≥60 x10^9/L
6. International Normalised Ratio (INR) ≤ 1.5, Activated Partial Thromboplastin Time (aPTT) ≤ 40 seconds, Prothrombin Time (PT) ≤13.5 seconds
7. Patient willing and able to comply with schedule visits, treatment plan and other study procedures.
8. Patients taking Riluzole must have discontinued treatment ≥28 days prior to study entry
9. Women Of Child Bearing Potential (WOCBP) who agree to use highly effective means of contraception (as defined in the Heads of Medicines Agencies_Clinical Trials Facilitation Group (HMA_CTFG) guideline (see Appendix 8) and in combination with a barrier contraception method (condom, diaphragm or cap) for the entirety of the study,

Exclusion Criteria:

1. Patients classified as either probable or possible ALS according to El Escorial Criteria (see Appendix 10).
2. Subjects in whom other causes of neuromuscular weakness have not been excluded
3. Assisted ventilation of any type within 3 months before the screening visit or at screening
4. Patients requiring Radiologically Inserted Gastrostomy (RIG) or Percutaneous Endoscopic Gastroscopy (PEG) feeding
5. Involvement in any other interventional study involving use of another IMP or biological product, within 3 months of screening
6. Any use of antioxidants, edaravone, tirasemtiv or CK-2127107 within 1 month before the screening visit.
7. Any botulinum toxin use within 3 months before the screening visit
8. Any form of stem cell or gene therapy for the treatment of amyotrophic lateral sclerosis (ALS)
9. Neuroimaging of brain and cervical spine with Magnetic Resonance imaging(MRI) indicating compressive myelopathy as an alternate diagnosis
10. Laboratory examinations including Acetylcholine receptor (AChR) antibodies and Muscle Specific Kinase (MuSK) antibodies to exclude Bulbar onset Myasthenia gravis from Bulbar onset Motor neuron disease as an alternate diagnosis and Antinuclear Antibodies (ANA), Anti-neutrophil cytoplasmic antibodies (ANCA), Extractable Nuclear Antigen (ENA) antibodies, Creatine Kinase (CK), electrophoresis and immunoglobulin indicating an alternate diagnosis for muscle disease like Myositis. It is at the Chief Investigators discretion to determine if positive results from any of the mentioned tests indicates an alternative diagnosis.
11. Abnormal liver function defined as AST and/or ALT >3 times upper limit of normal
12. Any head trauma, intracranial or spinal surgery within 3 months of trial entry
13. Patients who have had recurrent falls will be excluded to reduce the risk of intracerebral haemorrhage with this Investigational Medicinal Product (IMP)
14. Current use of an anticoagulant e.g Warfarin, Aspirin, Clopidogrel, any novel anticoagulants (NOAC)s or low molecular weight subcutaneous heparin
15. Uncontrolled severe hypertension defined as systolic blood pressure (SBP) ≥220 mmHg or diastolic blood pressure (DBP) ≥120 mmHg
16. Current or previous history of heparin-induced thrombocytopenia
17. Active peptic ulcer disease
18. Known hypersensitivity to sulphur
19. Severe liver insufficiency
20. Patients with evidence of major psychiatric illness, significant cognitive impairment or clinically evident dementia that may interfere with the patient's ability to comply with study procedures.

SAMPLE

21. Pulmonary illness (e.g. asthma or Chronic Obstructive Pulmonary Disease (COPD) requiring regular treatment
22. Patient judged to be actively suicidal by the investigator during 3 months before the screening visit
23. Subjects with a diagnosis of another neurodegenerative disease (e.g. Parkinson`s disease, Alzheimer's disease and Frontotemporal dementia)
24. Pregnant and/or breastfeeding

Treatment Extension Eligibility Criteria

The following must be met for patients to be considered for treatment extension beyond 10 weeks:

Inclusion:

1. Completed 10 weeks of treatment
2. Favourable toxicity profile, as reviewed by the PI
3. Forced Vital Capacity (FVC) ≥50% of predicted value for gender, height and age at screening and/or a mean Sniff Nasal Inspiratory Pressure (SNIP) ≥50% of predicted value for age
4. Patient willing to continue

Exclusion:

1. Meeting any of the patient stopping rules (see section 7.1.6)
2. Pregnancy/breast feeding
3. Patients who have had recurrent falls will be excluded to reduce the risk of intracerebral haemorrhage with this Investigational Medicinal Product (IMP)
4. Patients requiring Radiologically Inserted Gastrostomy (RIG) or Percutaneous Endoscopic Gastroscopy (PEG) feeding
5. use of an anticoagulant e.g Warfarin, Aspirin, Clopidogrel, any novel anticoagulants (NOAC)s or low molecular weight subcutaneous heparin
6. Worsening function status and unable to attend weekly outpatient appointments.

Trial Duration

Recruitment: 12 months

Treatment Duration: The initial trial period is 10 weeks, with the option to extend treatment following an assessment of the patient to confirm they do not meet any patient stopping criteria and are deemed eligible by the PI. Patients can potentially be treated up to a maximum of 48 weeks.

Due to the early discontinuation of treatment and follow up visits as a result of the COVID-19 pandemic, a single remote follow up visit for each patient will be starting in Quarter 1 of 2021. Patients will be required to re-consent for this additional visit and data collection.

Patient Stopping Rules:

Patients will have treatment of ILB stopped if any of the following prospective criteria are met **during the treatment period**:

- The patient experiences a Serious Adverse Event related to IMP
- If there is a decrease in ALSFRS-R score (>50%) compared to baseline
- If a patient becomes pregnant during the study treatment period

SAMPLE

- Unacceptable toxicity – any adverse event which, in the investigators opinion, requires termination of the study drug
- Unforeseen events – any event which in the judgement of the Investigator makes further treatment inadvisable
- SAE requiring discontinuation of treatment
- If a patient requires an anticoagulant e.g Warfarin, Aspirin, Clopidogrel, any novel anticoagulants (NOAC)s or low molecular weight subcutaneous heparin during the clinical trial
- PTT or aPTT > 1.5 x ULN
- INR > 1.8 x ULN

Patients, who stop weekly dosing, will be invited to remain in the observational part of the study up to 24 weeks, to enable assessment of safety related measures. Patients that discontinue ILB and subsequently start or re-commence Riluzole therapy will also be invited to remain in the observational part of the study up to 24 weeks.

Rescue Medication

In the event patients are discontinued from dosing with ILB, they will be offered (supportive) standard of care with the option of starting Riluzole therapy. Riluzole is a drug that affects several neurotransmitter systems and signalling pathways[1]. From in vitro studies[2-4] and the patient side-effect profile of the drug it is clear that Riluzole affects glutamatergic signalling in neurones and most importantly it inhibits Ca-dependent downstream signalling in these cells. This suggests that Riluzole inhibits the mechanisms that are pivotal for neuronal regeneration induced by ILB. Based on the mechanism of action of ILB Riluzole is predicted to inhibit activity of ILB. Therefore, patients discontinuing trial treatment of ILB must have a ≥7 day washout period before commencing Riluzole therapy due to the potential interaction between the two drugs.

Rescue medications will be administered outside the remit of the proposed study.

Trial Stopping Rules:

The clinical trial will be subject to periodic reviews by an independent safety monitoring committee. The trial will be suspended if any of the following conditions are met:

a) ≥1 patient in the first Sentinel patients experiences a Serious Adverse Event related to IMP
b) ≥33% of patients (with n > 3) recruited to the study show a significant decrease in ALSFRS-R score (>50%) compared to baseline during the 10-week dosing period.
c) ≥33% of patients (with n > 3) recruited to the study show a significant decrease in Quality of Life (ALSAQ-40) (>50%) compared to baseline during the 10 week dosing period.

In the event that the trial is suspended, the MHRA and REC will be advised and recruitment will not recommence until after submission and approval of a Substantial Amendment.

Follow-up: 2 weeks following final IMP administration

The trial was terminated early as a result of the COVID-19 pandemic. Therefore, a single remote follow up visit for each patient will be conducted, starting in Quarter 1 of2021 to collect Quality of Life and current medication data.

Trials Office Contact Details

ALS Trial Office
D[3]B – Diagnostics Drugs Devices & Biomarkers, CRCTU
Heritage Building
5th Floor East, ITM,
Mindelsohn Way,
Edgbaston,
Birmingham,
B15 2TH

Enquires
☎ +44(0) 121 371 8108
✉ ALS@trials.bham.ac.uk

SAMPLE

Trial Schema

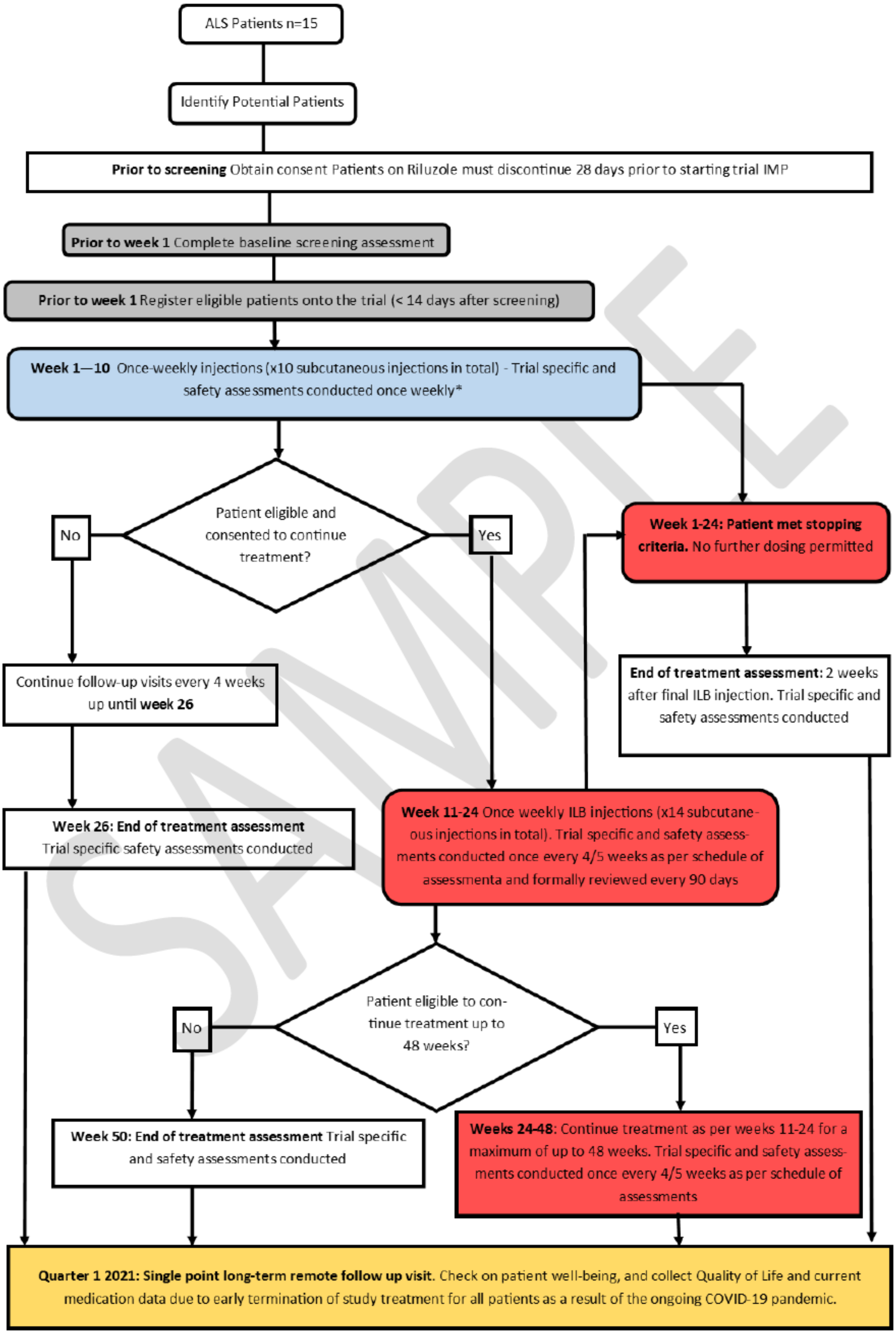

*Additional assessments conducted at week 5, week 9, week 14, week 19, week 24, end of treatment visit and subsequent additional weeks if treatment extended past 24 weeks (please see schedule of assessments table below)

SAMPLE

Schedule of Assessments (1) Main Trial

	Screening ≤ 14 days of Registration (up to 28 days prior to Day 1 for those patients taking Riluzole prior to trial entry)	Treatment Period**										Follow-up (Observational Period) following discontinuation of ILB*** All Follow-up visits (+/- 3 days)			
		Week 1	Week 2	Week 3	Week 4	Week 5	Week 6	Week 7	Week 8	Week 9	Week 10	Week 12****	Week 16	Week 20	Week 24****
Time schedule **Days +/- 1 day**	**Visit 1 Day ≤ 14**	**Visit 2 Day 1**	**Visit 3 Day 8**	**Visit 4 Day 15**	**Visit 5 Day 22**	**Visit 6 Day 29**	**Visit 7 Day 36**	**Visit 8 Day 43**	**Visit 9 Day 50**	**Visit 10 Day 57**	**Visit 11 Day 64**	**Visit 13 Day 78**	**Visit 14 Day 106**	**Visit 15 Day 134**	**Visit 16 Day 162**
1. Medical history*	X														
2. Eligibility Assessment	X	X													
3. ALSFRS-R[1]	X	X	X	X	X	X	X	X	X	X	X	X	X	X	X
4. Informed Consent ▲	X														
5. Registration♠		X													
6. Concomitant drug history	X	X	X	X	X	X	X	X	X	X	X	X	X	X	X
7. Vital signs[2]	X	X	X	X	X	X	X	X	X	X	X	X	X	X	X
8. ECG	X					X					X	X			
9. Lung Function Tests (FVC) [3]	X					X				X		X			X
10. Sniff Nasal Inspiratory Pressure (SNIP) [4]	X					X				X		X			X
11.Haematology Tests[5]	X	X	X	X	X	X	X	X	X	X	X	X	X	X	X

	Screening ≤ 14 days of Registration (up to 28 days prior to Day 1 for those patients taking Riluzole prior to trial entry)	Treatment Period**										Follow-up (Observational Period) following discontinuation of ILB*** All Follow-up visits (+/- 3 days)			
		Week 1	Week 2	Week 3	Week 4	Week 5	Week 6	Week 7	Week 8	Week 9	Week 10	Week 12****	Week 16	Week 20	Week 24****
Time schedule **Days +/- 1 day**	**Visit 1 Day ≤ 14**	**Visit 2 Day 1**	**Visit 3 Day 8**	**Visit 4 Day 15**	**Visit 5 Day 22**	**Visit 6 Day 29**	**Visit 7 Day 36**	**Visit 8 Day 43**	**Visit 9 Day 50**	**Visit 10 Day 57**	**Visit 11 Day 64**	**Visit 13 Day 78**	**Visit 14 Day 106**	**Visit 15 Day 134**	**Visit 16 Day 162**
12. Coagulation Tests[6]	X	X	X	X	X	X	X	X	X	X	X	X	X	X	X
13 Biochemistry Tests[7]	X	X	X	X	X	X	X	X	X	X	X	X	X	X	X
14. Creatine Kinase (serum)	X					X					X	X			
15.U&Es & LFTs[8]	X	X	X	X	X	X	X	X	X	X	X	X	X	X	X
16.Thyroid Function Tests[9]	X										X	X			X
17.Immunoglobulins [10]	X										X	X			
18.Quality of Life Questionnaire[11]	X	X	X	X	X	X	X	X	X	X	X	X	X	X	X
19. Urinary p75[ECD12]		X				X					X	X			X
20. NfL levels[13]		X				X					X	X			
21. Exploratory Biomarkers (serum and PBMC)[14]		X				X					X	X			X
22. Pregnancy test (for WOCBP only)	X	X			X				X		X	X			
23.PK Sampling[15]		X													

	Screening ≤ 14 days of Registration (up to 28 days prior to Day 1 for those patients taking Riluzole prior to trial entry)	Treatment Period**										Follow-up (Observational Period) following discontinuation of ILB*** All Follow-up visits (+/- 3 days)			
		**Week 1**	**Week 2**	**Week 3**	**Week 4**	**Week 5**	**Week 6**	**Week 7**	**Week 8**	**Week 9**	**Week 10**	**Week 12******	**Week 16**	**Week 20**	**Week 24******
Time schedule **Days +/- 1 day**	**Visit 1 Day ≤ 14**	**Visit 2 Day 1**	**Visit 3 Day 8**	**Visit 4 Day 15**	**Visit 5 Day 22**	**Visit 6 Day 29**	**Visit 7 Day 36**	**Visit 8 Day 43**	**Visit 9 Day 50**	**Visit 10 Day 57**	**Visit 11 Day 64**	**Visit 13 Day 78**	**Visit 14 Day 106**	**Visit 15 Day 134**	**Visit 16 Day 162**
24. Dynamometer test	X	X	X	X	X	X	X	X	X	X	X				
25. Study drug administration 2mg /kg s.c.[16]		X ♠	X	X	X	X	X	X	X	X	X				
26. Adverse Event/Toxicity Reporting		Throughout						Throughout				Throughout			

Schedule of Assessments (2) Treatment Extension (Week 11 to Week 24)

**Schedule of Assessments**	**Week 11**	**Week 12**	**Week 13**	**Week 14**	**Week 15**	**Week 16**	**Week 17**	**Week 18**	**Week 19**	**Week 20**	**Week 21**	**Week 22**	**Week 23**	**Week 24**	**Week 26******	**Treatment continuation#**
Time schedule **Days +/- 1 day**[≠]	**Day 71**	**Day 78**	**Day 85**	**Day 92**	**Day 99**	**Day 106**	**Day 113**	**Day 120**	**Day 127**	**Day 134**	**Day 141**	**Day 148**	**Day 155**	**Day 162**	**End of treatment visit Only for those patients completing up to 48 weeks of treatment**	**Treatment beyond 24 weeks will be a discussion between patient and clinician. If treatment is continued, treatment and visits will follow the same pattern as week 11-24 for a maximum of 48 weeks**
1. Re-consent	X															
2. ALSFRS-R[1]	X			X					X					X	X	
3. Concomitant drug history	Throughout															
4. Vital signs[2]				X					X					X	X	
5. ECG				X					X					X	X	
6. Lung Function Tests (FVC)[3]				X					X					X	X	
7. Sniff Nasal Inspiratory Pressure (SNIP)[4]				X					X					X	X	
8.Haematology Tests[5]				X					X					X	X	
9. Coagulation Tests[6]				X					X					X	X	
10 Biochemistry Tests[7]				X					X					X	X	
11. Creatinine Kinase (serum)				X					X					X	X	
12.U&Es & LFTs[8]				X					X					X	X	
13.Thyroid Function Tests[9]				X					X					X	X	

Schedule of Assessments	Week 11	Week 12	Week 13	Week 14	Week 15	Week 16	Week 17	Week 18	Week 19	Week 20	Week 21	Week 22	Week 23	Week 24	Week 26****	Treatment continuation#
Time schedule **Days +/- 1 day**[≠]	Day 71	Day 78	Day 85	Day 92	Day 99	Day 106	Day 113	Day 120	Day 127	Day 134	Day 141	Day 148	Day 155	Day 162	**End of treatment visit** Only for those patients completing up to 48 weeks of treatment	Treatment beyond 24 weeks will be a discussion between patient and clinician. If treatment is continued, treatment and visits will follow the same pattern as week 11-24 for a maximum of 48 weeks
14.Immunoglobulins[10]				X					X					X	X	
15.Quality of Life Questionnaire[11]				X					X					X	X	
16 Urinary $p75^{ECD}$[12]														X		
17. NfL levels[13]														X	X	
18. Exploratory Biomarkers (serum and PBMC)[14]														X	X	
19. Pregnancy test (for WOCBP only)				X					X					X	X	
20. Dynamometer test				X					X					X	X	
21. Study drug administration 2mg /kg bw s.c.[15]	X	X	X	X	X	X	X	X	X	X	X	X	X	X		
23. Adverse Event/Toxicity Reporting	Throughout															

Schedule of Assessments (2) Treatment Extension (Week 25 to Week 38)

**Schedule of Assessments**	**Week 25**	**Week 26**	**Week 27**	**Week 28**	**Week 29**	**Week 30**	**Week 31**	**Week 32**	**Week 33**	**Week 34**	**Week 35**	**Week 36**	**Week 37**	**Week 38**	**Week 40******	**Treatment continuation**
Time schedule **Days +/- 1 day[≠]**	**Day 169**	**Day 176**	**Day 183**	**Day 190**	**Day 197**	**Day 204**	**Day 211**	**Day 218**	**Day 225**	**Day 232**	**Day 239**	**Day 246**	**Day 253**	**Day 260**	**<u>End of treatment visit</u> Only for those patients completing up to 38 weeks of treatment**	**Extended treatment will be a discussion between patient and clinician. If treatment is continued, treatment and visits will follow the same pattern as week 11-24 for a maximum of 48 weeks**
1. Re-consent																
2. ALSFRS-R[1]				X					X					X	X	
3. Concomitant drug history	Throughout															
4. Vital signs[2]				X					X					X	X	
5. ECG				X					X					X	X	
6. Lung Function Tests (FVC)[3]				X					X					X	X	
7. Sniff Nasal Inspiratory Pressure (SNIP) [4]				X					X					X	X	
8.Haematology Tests[5]				X					X					X	X	
9. Coagulation Tests[6]				X					X					X	X	
10 Biochemistry Tests[7]				X					X					X	X	
11. Creatinine Kinase (serum)				X					X					X	X	
12.U&Es & LFTs[8]				X					X					X	X	
13.Thyroid																

**Schedule of Assessments**	**Week 25**	**Week 26**	**Week 27**	**Week 28**	**Week 29**	**Week 30**	**Week 31**	**Week 32**	**Week 33**	**Week 34**	**Week 35**	**Week 36**	**Week 37**	**Week 38**	**Week 40******	**Treatment continuation**
Time schedule **Days +/- 1 day**‡	**Day 169**	**Day 176**	**Day 183**	**Day 190**	**Day 197**	**Day 204**	**Day 211**	**Day 218**	**Day 225**	**Day 232**	**Day 239**	**Day 246**	**Day 253**	**Day 260**	**End of treatment visit Only for those patients completing up to 38 weeks of treatment**	**Extended treatment will be a discussion between patient and clinician. If treatment is continued, treatment and visits will follow the same pattern as week 11-24 for a maximum of 48 weeks**
Function Tests[9]																
14.Immunoglobulins[10]				X					X					X	X	
15.Quality of Life Questionnaire[11]				X					X					X	X	
16 Urinary p75^{ECD}[12]														X		
17. NfL levels[13]														X	X	
18. Exploratory Biomarkers (serum and PBMC)[14]														X	X	
19. Pregnancy test (for WOCBP only)				X					X					X	X	
20. Dynamometer test				X					X					X	X	
21. Study drug administration 2mg /kg bw s.c.[15]	X	X	X	X	X	X	X	X	X	X	X	X	X	X		
23. Adverse Event/Toxicity Reporting	Throughout															

Schedule of Assessments (2) Treatment Extension (Week 39 to Week 48)

**Schedule of Assessments**	**Week 39**	**Week 40**	**Week 41**	**Week 42**	**Week 43**	**Week 44**	**Week 45**	**Week 46**	**Week 47**	**Week 48**	**Week 50******
Time schedule **Days +/- 1 day**ǂ	**Day 267**	**Day 274**	**Day 281**	**Day 288**	**Day 295**	**Day 302**	**Day 309**	**Day 316**	**Day 323**	**Day 330**	**<u>End of treatment visit</u> Only for those patients completing up to 48 weeks of treatment**
1. Re-consent											
2. ALSFRS-R[1]					X					X	X
3. Concomitant drug history	Throughout										
4. Vital signs[2]					X					X	X
5. ECG					X					X	X
6. Lung Function Tests (FVC)[3]					X					X	X
7. Sniff Nasal Inspiratory Pressure (SNIP) [4]					X					X	X
8.Haematology Tests[5]					X					X	X
9. Coagulation Tests[6]					X					X	X
10 Biochemistry Tests[7]					X					X	X
11. Creatinine Kinase (serum)					X					X	X
12.U&Es & LFTs[8]					X					X	X
13.Thyroid Function Tests[9]											
14.Immunoglobulins[10]					X					X	X
15.Quality of Life Questionnaire[11]					X					X	X

**Schedule of Assessments**	**Week 39**	**Week 40**	**Week 41**	**Week 42**	**Week 43**	**Week 44**	**Week 45**	**Week 46**	**Week 47**	**Week 48**	**Week 50******
Time schedule **Days +/- 1 day**[≠]	**Day 267**	**Day 274**	**Day 281**	**Day 288**	**Day 295**	**Day 302**	**Day 309**	**Day 316**	**Day 323**	**Day 330**	**End of treatment visit Only for those patients completing up to 48 weeks of treatment**
16 Urinary p75[ECD12]											
17. NfL levels[13]											X
18. Exploratory Biomarkers (serum and PBMC)[14]											X
19. Pregnancy test (for WOCBP only)					X					X	X
20. Dynamometer test					X					X	X
21. Study drug administration 2mg /kg bw s.c.[15]	X	X	X	X	X	X	X	X	X	X	
23. Adverse Event/Toxicity Reporting	Throughout										

Schedule of Assessments – Single Point Long-Term Remote Follow Up Visit

Schedule of Assessments	
Time Schedule (8 week period)	Quarter 1 2021
1. Re-consent	X
2. ALSFRS-R[1]	X
3. Quality of Life Questionnaire[11]	X
4. Concomitant Medication	X

SAMPLE

Schedule of Events

*to include an assessment of any mental or physical contraindications to study entry

** For patients that discontinue treatment prior to Week 10, follow-up should continue 4 weekly, after the end of treatment visit, up to week 24. These visits should all follow the week 16 assessments apart from the final visit which follows week 24.

*** If patients do not continue treatment after week 10, follow the Follow-up (Observational Period) schedule in Schedule of Assessments (1) Main Trial. If the criteria is met for IMP treatment to continue beyond week 10, follow Schedule of Assessments (2): Treatment Extension after week 10.

**** End of treatment visit should occur 2 weeks after last dose of IMP (as per the Week 12 assessments) regardless of how many treatment doses a patient has received.

Patients that reach week 24 of treatment and are still eligible, the patient will discuss their future treatment options with the clinician and decide together whether to continue treatment which can be extended up to a maximum of 48 weeks. A formal review will be held every 90 days to decide whether continuation of treatment is in the best interest of the patient.

▲ Patients on Riluzole prior to study entry must discontinue treatment on the day of consent and must have a 28 day wash-out period (i.e. discontinue Riluzole ≥28 days prior to commencing study drug). Screening tests in this patient group can commence up to 28 days prior to Day 1 but study drug must not be commenced until 28 days has elapsed since last dose of Riluzole

♠ Registration and the start of study drug dosing (Week 1, Day 1) must not be < 28 days from the last dose of Riluzole

≠ If patients have an interruption to treatment the time schedule in days pauses until they recommence treatment.

1 The Amyotrophic Lateral Sclerosis Functional Rating Scale (Revised) (ALSFRS-R)

2 Vital Signs (must be performed prior to administration of study drug) – Blood pressure, heart rate, body temperature, pulse oximetry and respiration rate. Results of oximetry must be the average of three recordings. Body weight, height (at screening only) and Body Mass Index (BMI)

3 Forced Vital Capacity (FVC)

4 Sniff Nasal Inspiratory Pressure (SNIP) results must be recorded as the mean of three recordings. The same nostril used at baseline must be used for subsequent visits/assessments

5 To include Full Blood Count (FBC), Erythrocyte Sedimentation Rate (ESR) (must be performed prior to administration of study drug)

6 To include International Normalized Ratio (INR), Activated Partial Thromboplastin Time (APTT) and Prothrombin Time (PT) (must be performed prior to administration of study drug)

7 Biochemistry tests to include: Troponin I (up to 24 weeks), C-reactive protein (CRP), Glucose, Calcium (must be performed prior to administration of study drug)

8 Urea and Electrolytes (U&Es), Liver Function Tests (LFTs), (must be performed prior to administration of study drug)

SAMPLE

9 To include Thyroid Stimulating Hormone (TSH), Free Thyroxine (Free T4)

10 Immunoglobulins (IgG, IgM)

11 ALS Assessment Questionnaire (ALSAQ-40) (must be performed prior to administration of study drug)

12 Urinary $p75^{ECD}$ (must be performed prior to administration of study drug)

13 Neurofilament Light Chain (NfL) sample - Collect whole blood to allow for 4-5ml plasma for analysis (must be performed prior to administration of study drug)

14 Collection of Exploratory Biomarker blood samples (must be performed prior to administration of study drug). See Appendix 12 and the latest version of the Laboratory Manual for full details

15 Pharmacokinetic (PK) sample

16 The time of study drug administration will be recorded at each visit. All subsequent doses must be given within +/- 24 hours of the Day 1 Week 1 administration time

SAMPLE

ABBREVIATIONS

AA	Amino Acids
ABPI	Association of the British Pharmaceutical Industry
ACCG	Amino-group containing compounds
ACHR	Acetylcholine Receptor
ADL	Activities of Daily Living
AE	Adverse Event
ALP	Alkaline Phosphate
ALS	Amyotrophic Lateral Sclerosis
ALSAQ-40	Amyotrophic Lateral Sclerosis Assessment Questionnaire (40 item/questions)
ALSFRS-R	Amyotrophic Lateral Sclerosis Functional Rating Scale (revised)
ALT	Alanine Transaminase
ANA	Antinuclear Antibodies
ANCA	Anti-neutrophil cytoplasmic antibodies
API	Active Pharmaceutical Ingredient
APTT	Activated Partial Thromboplastin Time
AST	Aspartate Aminotransferase
BDNF	Brain Derived Neurotrophic Factor
BBB	Blood Brain Barrier
BMI	Body Mass Index
CK	Creatine Kinase
CK-MB	(serum) Creatine Kinase – Muscle, Brain
CNTF	Ciliary Neurotrophic Factor

SAMPLE

COPD	Chronic Obstructive Pulmonary Disease
CRCTU	Cancer Research UK Clinical Trials Unit
CRF	Case Report Form
CRP	C-reactive protein
CSF	Cerebrospinal Fluid
CTCAE	Common Terminology Criteria for Adverse Events
CTFG	Clinical Trials Facilitation Group
CV	Curriculum Vitae
DMC	Data Monitoring Committee
DS	Dextran Sulfate
DSUR	Developmental Safety Update Report
D^3B	Diagnostics, Drugs, Devices and Biomarkers
ECD	Extracellular Domain
ECG	Electrocardiogram
EGFR	Estimated Glomerular Filtration Rate
ELISA	Enzyme-Linked Immunosorbent Assay
EMG	Electromyography
ENA	Extractable Nuclear Antigen
eRDC	Electronic Remote Data Capture
ESR	Erythrocyte Sedimentation Rate (ESR)
FBC	Full Blood Count
FDA	Food and Drug Administration
FGF	Fibroblast Growth Factor
FVC	Forced Vital Capacity

SAMPLE

GCP	Good Clinical Practice
G-CSF	Granulocyte Colony Stimulating Factor
GGT	Gamma-Glutamyl Transpeptidase
GLP	Good Laboratory Practice
GMP	Good Manufacturing Practice
GP	General Practitioner
Hb	Haemoglobin
HGF	Hepatocyte Growth Factor
HLI	Hind-limb Ischemia
HMA	Heads of Medicines Agencies
HPLC	High Performance Liquid Chromatography
HrHGF	Human Recombinant Hepatocyte Growth Factor
HUVEC	Human Umbilical Vein Endothelial Cells
IB	Investigator Brochure
ICF	Informed Consent Form
IGF-1	Insulin-like Growth Factor 1
IgG	Immunoglobulin G
IgM	Immunoglobulin M
IMP	Investigational Medicinal Product
INR	International Normalised Ratio
ISF	Investigator Site File
ITM	Institute of Translational Medicine
i.v	Intravenous
kDa	kilo Dalton

SAMPLE

LFT	Liver Function Tests
LMN	Lower Motor Neuron
LMW-DS	Low Molecular Weight Dextran Sulfate
LPLV	Last Patient Last Visit
MBP	Myelin Basic Protein
MCV	Mean Cell Volume
Mg	Milligram
MHRA	Medicines and Healthcare Products Regulatory Agency
Ml	Millilitre
MND	Motor Neurone Disease
MS	Multiple Sclerosis
MUSK	Muscle-Specific Kinase
NAA	N-acetyl-aspartate
NaCL	Sodium Chloride
NCS	Nerve Conduction Study
NFL	Neurofilament, light
NHS	National Health Service
NOAC	Novel Anticoagulants
$P75^{ECD}$	Extracellular domain of p75
PBMC	Peripheral Blood Mononuclear Cells
PEG	Percutaneous Endoscopic Gastrostomy
PCR	Polymerase Chain Reaction
pH	Potential of Hydrogen
PI	Principal Investigator

SAMPLE

PIS	Patient Information Sheet
PK	Pharmacokinetics
PT	Prothrombin Time
QOL	Quality of Life
REC	Research Ethics Committee
RIG	Radiologically Inserted Gastrostomy
RNA	Ribonucleic Acid
SAE	Serious Adverse Event
SAR	Serious Adverse Reaction
SC	Subcutaneous
sncRNA	Small Non-coding RNA
SNIP	Sniff Nasal Inspiratory Pressure
SUSAR	Suspected Unexpected Serious Adverse Reaction
T4	Thyroxine
TMG	Trial Management Group
TSC	Trial Steering Committee
TSH	Thyroid Stimulating Hormone
UE	Urea and Electrolytes
UK	United Kingdom
µl	Microliter
ULN	Upper Limit of Normal
UMN	Upper Motor Neuron
VEGF	Vascular Endothelial Growth Factor
WBC	White Blood Cell Count

SAMPLE

WMA	World Medical Association
WOCBP	Women Of Child Bearing Potential

SAMPLE

Table of Contents

SPONSOR ii
Signature page iii
AMENDMENTS iv
Trial Synopsis ix
Trial Schema xv
Schedule of Assessments (1) Main Trial xvii
Schedule of Assessments (2) Treatment Extension (Week 11 to Week 24) xx
Schedule of Assessments (2) Treatment Extension (Week 25 to Week 38) xxii
Schedule of Assessments (2) Treatment Extension (Week 39 to Week 48) xxiv
Schedule of Assessments – Single Point Long-Term Remote Follow Up Visit xxvi
Schedule of Events xxvii
Abbreviations xxix
1 Background and Rationale 39
1.1 Background 39
1.1.1 Amyotrophic Lateral Sclerosis 39
1.1.2 Treatment for ALS patients 39
1.1.3 ILB 40
1.1.4 Mechanism of action 40
1.1.5 Previous human experience with ILB 41
1.2 Trial Rationale 42
1.2.1 Justification for patient population 42
1.2.2 Justification for design 43
1.2.3 Choice of treatment 43
1.2.4 Justification for single point long-term remote follow up visit 46
2. Objectives and Outcome Measures 47
2.1 Primary Objective 47
2.2 Secondary Objectives 47
2.3 Primary Outcome Measures 47
2.4 Secondary Outcome Measures 47
2.5 Exploratory Outcome Measures 48
3. Trial Design 49
4. Eligibility 50
4.1 Main Eligibility criteria 50
4.2 Treatment Extension Eligibility Criteria 52
5. Consent and Screening 53
5.1 Informed Consent 53
5.1.1 Treatment Extension 53
5.1.2 Single Point Long-Term Remote Follow Up 54
5.2 Screening 54
6. Trial Entry 55
6.1 Registration 55
6.1.1 Emergency Registration 55
6.1.2 Protocol waivers or exemptions 56
6.1.3 Treatment Extension registration 56
7. Treatment Details 57
7.1 Investigational Medicinal Product 57

SAMPLE

7.1.1 Dosage, Form and Composition ... 57
7.1.2 Packaging and Labelling ... 57
7.1.3 Storage and handling ... 57
7.1.4 Trial Treatment ... 58
7.1.5 Continuation of treatment beyond 10 weeks
7.1.6 Patient Stopping Rules ... 58
7.1.7 Rescue Medication ... 58
7.1.8 Special Warnings and Special Precautions for use ... 58
7.2 Treatment Schedule ... 59
7.3 Assessments ... 65
7.3.1 Blood chemistry and Haematology ... 65
7.3.1.1 Liver Chemistry Management ... 65
7.3.2 Physical examination/symptom assessment ... 66
7.3.3 Electrocardiogram (ECG) ... 66
7.3.4 Lung Function Tests ... 66
7.3.5 Quality of Life ... 66
7.3.6 Pregnancy testing ... 67
7.4 Sample Collection ... 67
7.4.1 Blood samples ... 67
7.4.2 Urine Samples ... 69
7.5 Dose Modifications ... 69
7.6 Treatment Interruptions ... 69
7.7 Treatment Compliance ... 69
7.8 Treatment Discontinuation ... 70
7.9 Treatment after the end of the study ... 70
7.10 Supportive Treatment ... 70
7.11 Concomitant Medication ... 70
7.12 Prohibited Medication ... 70
7.13 Contraception and Pregnancy ... 70
7.14 Patient Follow Up ... 71
7.15 Patient Withdrawal ... 75
8. Adverse Event Reporting ... 76
8.1 Reporting Requirements ... 76
8.1.1 Adverse Events ... 76
8.1.1.1 Pre-existing conditions ... 76
8.1.2 Serious Adverse Events ... 76
8.1.2.2 Liver Related Toxicity: Biochemistry ... 77
8.1.3 Reporting period ... 77
8.2 Reporting Procedure ... 78
8.2.1 Site ... 78
8.2.2 Trials Office ... 79
8.2.3 Reporting to the Competent Authority and Research Ethics Committee ... 79
8.2.4 Investigators ... 79
8.2.5 Data Monitoring Committee ... 79
8.2.6 Manufacturer of Investigational Medicinal Product ... 79
9. Data Handling and Record Keeping ... 80
9.1 Data Collection ... 80
9.2 Data Collection Procedure ... 82

SAMPLE

9.2.1 Electronic Remote Data Capture (eRDC) systems only 82
9.2.2 Paper Case Report Forms only 82
9.3 Archiving 83
10. Quality Management 84
10.1 Site Set-up and Initiation 84
10.2 On-site Monitoring 84
10.3 Central Monitoring 84
10.4 Audit and Inspection 84
10.5 Notification of Serious Breaches 85
11. End of Trial Definition 86
12. Statistical Considerations 86
12.1 Definition of Outcome Measures 86
12.2 Analysis of Outcome Measures 86
12.2.1 Analysis of Primary Outcome Measures 86
12.2.2 Analysis of Secondary Outcome Measures 86
12.3 Planned Sub Group Analyses 87
12.4 Planned Interim Analysis 87
12.5 Planned Final Analyses 87
12.6 Power Calculations 87
13. Trial Organisational Structure 88
13.1 Sponsor 88
13.2 Coordinating Centre 88
13.3 Trial Management Group 88
13.4 Trial Steering Committee 88
13.5 Data Monitoring Committee 88
13.6 Finance 89
13.6.1 Payments to individual NHS Trusts (per-patient payment) 89
14. Ethical Considerations 90
15. Confidentiality and Data Protection 91
16. Insurance and Indemnity 92
17. Publication Policy 92
18. Reference List 93
Appendix 1 – ALS Functional rating scale revised (ALSFRS-R) 96
Appendix 2 - WMA Declaration of Helsinki 97
Appendix 3 - Definition of Adverse Events 100
Appendix 4 - Common Toxicity Criteria Gradings 102
Appendix 5 – Liver safety required actions and follow-up assessments 103
Appendix 6 – ALS Assessment Questionnaire (ALSAQ-40) 105
Appendix 7 – EQuation for estimated Glomerular filtration Rate 106
Appendix 8 – HMA CTFG guidelines on contraception 107
Appendix 9 – El escorial world federation of neurology criteria for the diagnosis of als 109
Appendix 10 – Sniff nasal inspiratory pressure (snip) & Forced vital capacity (FVC) 126
Appendix 11 – Targeted metabolic markers in serum (And urine p75) 127

SAMPLE

LIST OF FIGURES

Figure 1 Chemical structure of ILB 40
Figure 2 HGF levels at 30 min to 9 hours after administration of 3-24 mg/kg ILB in human (clinical study LMW-DS-102) 45

LIST OF TABLES

Table 1 Mean pharmacokinetic exposure parameters in plasma from healthy volunteers 44
Table 2 Dose Modifications for Adverse Events 69
Table 3 ALS Trial Case Report Form 80

SAMPLE

1 BACKGROUND AND RATIONALE

1.1 Background

1.1.1 Amyotrophic Lateral Sclerosis

Amyotrophic Lateral Sclerosis (ALS), also known as Lou Gehrig´s disease is a rare neurological disease and is the most common Motor Neurone Disease. It is a fatal disease with specific loss of motor neurons in the spinal cord, brain stem and motor cortex leading to progressive paralysis and usually death within five years of diagnosis. The disease begins focally in the central nervous system and then spreads relentlessly. The clinical diagnosis, defined by progressive signs and symptoms of upper and lower motor neuron dysfunction, is confirmed by clinical findings, electromyography, blood and cerebrospinal fluid (CSF) analysis. Additional testing with for instance Magnetic Resonance Imaging (MRI) excludes other conditions. The disease is heterogeneous, but most patients die of respiratory muscle weakness less than 3-5 years from symptom-onset. Like other age-related neurodegenerative diseases, ALS has genetic, metabolic and environmental triggers.

Of the 5-10% of cases that are inherited, mutations have been discovered for a high proportion. In addition to genetic factors, metabolic factors, inflammatory diseases, age, tobacco use, and athleticism may contribute to sporadic ALS, but important aetiologies are unidentified for most patients. ALS disease contains postulated pathophysiological mechanisms, including mitochondrial dysfunction, aggregation of misfolded protein, oxidative stress, excitotoxicity, inflammation and apoptosis, and involve both motor neurons and surrounding glial cells. There is clinical and pathological overlap with other neurodegenerative diseases, particularly frontotemporal dementia. The mechanisms leading to disease propagation in the brain are a current focus of research.

Many neurotrophic growth factors are known to promote survival of neurons and various factors have been investigated both pre-clinically and clinically for the treatment of ALS. Yet no substantial positive effects upon growth factor-therapy have been observed in clinical studies using ciliary neurotrophic factor (CNTF), brain derived neurotrophic factor (BDNF), insulin-like growth factor (IGF-1), or granulocyte colony stimulating factor G-CSF. But, for various reasons, in these studies the dose and/or the administration route were not optimized for therapeutic efficacy, hence, results should be interpreted carefully[5]. Studies in the rat ALS-model using human recombinant hepatocyte growth factor (hrHGF) have shown functional recovery in the animals[6]. HGF is a pleiotropic growth factor and also a potent survival-promoting factor for motor neurons. In the first clinical study performed ILB was found to increase (up to 100-fold) plasma levels of hepatocyte growth factor (HGF) in healthy volunteers[7]. This effect was verified in a number of clinical studies.[8, 9]

1.1.2 Treatment for ALS patients

There is as yet no cure for ALS, and management is focused on a combination of neuroprotective medication, multidisciplinary clinics and respiratory support. Speciality clinics for ALS emerged in the 1980s and most large centres in developed countries currently offer multidisciplinary care[10]. Patients treated by ALS care teams may have higher quality of life[11] and longer survival[12]. Malnutrition and dehydration are common as ALS advances. Later therapy often includes percutaneous gastric feeding and non-invasive ventilatory support.

To date, one medication (Riluzole, licensed in 1996) has been proven to prolong survival somewhat in ALS[13]. Riluzole was developed because it possesses anti-glutamatergic properties that might reduce

excitotoxicity in ALS. Riluzole slowed disease progression in two randomized controlled trials,[14, 15] and prolongs survival in patients with ALS by about 2-3 months[13]. The most common side effects are exanthema, diarrhoea, dizziness, fatigue, nausea, and somnolence. Elevation of liver enzymes can occur, but rarely to levels that are clinically meaningful. Many patients in Europe and more than half of ALS patients in the U.S. take riluzole[16].

Numerous trials have so far been unable to identify another neuroprotective agent. Researchers now aim to slow disease progression by targeting known pathophysiological pathways or genetic defects. Until there is better understanding of the causes and mechanisms underlying progression that lead to more robust neuroprotective agents, symptomatic therapies can extend life and improve quality of life. Palliative care providers such as hospices give emotional and physical support to patients and families throughout much of the disease course[17].

1.1.3 ILB

During the development, this specific molecule and solutions of this molecule have been known under several names: DSSS5, ILB, TM-500, TM-700, LMW-DS and IBsolvMIR. These are all different names of exactly the same molecule and solutions of this molecule that are collectively referred to as ILB.

The investigational product, ILB, is a sulfated form of polymerised anhydroglucose which contains approximately 17% sulphur. The backbone of the chain may vary in length. ILB has an average molecular weight of 5,000 Dalton and is negatively charged.

The ILB formulation for subcutaneous injection contains 100 mg/ml of the active substance and 9 mg/ml of NaCl and is a hypertonic solution.

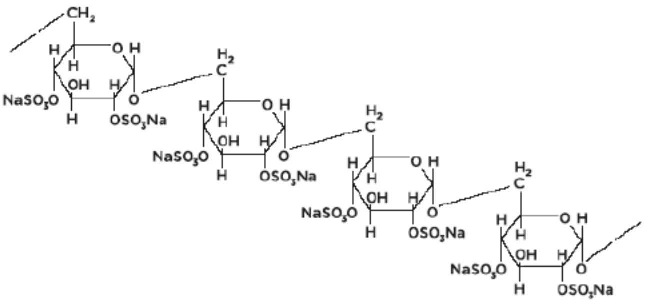

Figure 1 Chemical structure of ILB

1.1.4 Mechanism of action

Motor neurone dysfunction and degeneration in ALS involves several pathogenic mechanisms, which include disturbed energy metabolism, cytoskeletal abnormalities, changes in transcription, glutamate excitotoxicity, glial hyper activation and reduced glutamate uptake[18]. In our in vitro models we found that ILB is able to modulate molecular processes relevant to ALS[2-4] generally enhancing the protective effect of growth factors. In glial cells the ILB reduces oxidative stress, mitochondrial dysfunction and glial activation. It also promotes cell survival, differentiation, myelin basic protein (MBP) expression and glutamate uptake. In neuronal cultures ILB reduces neuronal cell death, oxidative stress and mitochondrial dysfunction. In neurones the compound also promotes differentiation, neurite outgrowth and cellular homeostasis. Most importantly glutamate production and release in neuronal cells is NOT affected by ILB.

Glutamate production and release (followed by Ca-mediated signalling cascades) in neuronal cells is one of the most important factors required for synaptic remodelling necessary for regeneration and repair of neuronal networks affected in ALS (and indeed all neurodegenerative diseases). This process is controlled by the growth factors modulated by ILB and leads to the neurite outgrowth and differentiation of these cells. This is the process that will lead to a measurable functional benefit in the nervous system and clinical improvement in the patients.

1.1.5 Previous human experience with ILB

Dextran sulfate was originally developed and explored more than 50 years ago for its anticoagulant properties[19].Since then, it has been shown to have a number of additional clinical effects and low molecular weight dextran sulfate (LMW-DS), has clinically been investigated as an antiviral compound[20, 21],in patients with post-perfusion lung damages[22],and in stroke patients[23].

ILB has been evaluated in a phase I, open single-centre study in healthy volunteers (n=34)[7]. This study was designed to find the optimal dosing regimen for maintaining predetermined plasma concentrations of ILB during a 5-hour infusion period, with the intention to be used as guidance for single-dose short-term administration in the subsequent phase II study in diabetic patients (n=10). The results of the phase I study indicate that ILB in doses that maintain APTT up to 150 seconds for 5 hours, corresponding to a total exposure of approximately 15 mg/kg, can be safely infused with no signs of increased bleeding or other severe side effects.

An open dose-finding study in healthy male volunteers (n=24) was performed with the objective to evaluate pharmacokinetics, safety, tolerability and pharmacodynamics effects of mobilization of CD34+ cells, lymphocytes and HGF[8]. The study was divided into two parts, Part A and Part B. In Part A, the doses 3,6,9,12 and 15 mg/kg of ILB was administered as single 10-minutes` infusions to 3 subjects per dose level (15 subjects in total). In Part B, 3 subjects received 18 mg/kg and 6 subjects received 24 mg/kg. ILB was well tolerated and showed no serious adverse events in the investigated doses (up to 24 mg/kg). ILB caused an increase in both HGF and lymphocyte levels, but did not fulfil the intended 4-fold increase in CD34+ stem cell mobilisation. The half-life in human was again confirmed to be approximately 3 hours.

A randomised, open-label, placebo-controlled, single centre study in healthy male volunteers was performed[9]. The aim was to evaluate the efficacy, safety and tolerability of ILB treatment in combination with the current standard hematopoietic mobilisation therapy, subcutaneous (s.c) injections of filgrastim once daily for 5 days. ILB treatment, when administered as a single 10 minutes` infusion (18 mg/kg) following 5 days of once daily s.c. treatment with filgrastim, was compared to a single s.c. injection of plerixafor or a 10 minutes` infusion of NaCl (control) following 5 days of once daily s.c. treatment with filgrastim. A stem cell mobilising effect of ILB superior to the current standard was not achieved, and the study was ended prematurely. No safety issues were reported in the administered 18mg/kg dose.

A phase I study was performed in healthy male volunteers (n=12)[24]. The aim was to investigate the pharmacokinetics and bioavailability of ILB after s.c. administration compared to the i.v. administration route, as well as assessing the local tolerability at the s.c. injection site. The s.c. injection was well tolerated with only mild, transient local effects. Despite no prior administration of antihistamines, no hypersensitivity reactions were observed. Altogether, the bioavailability and tolerability found in the present study with s.c. administered ILB, justify the use of this formulation in clinical studies. The pharmacokinetics corresponded well with data from previous clinical and nonclinical studies with a lower and delayed maximum plasma concentration, and a longer half-life for the s.c. as compared to the i.v. administration. The s.c. injections was well tolerated with only mild, transient local effects. No hypersensitivity reactions were observed.

A phase II open, randomised, multi-center study, was performed to investigate the effects of ILB in pancreatic islet transplantation in diabetes type 1 patients compared to heparin (i.e. conventional

SAMPLE

treatment)[25]. In this study, 19 transplantations (repetitive transplantations are included in this number) with ILB was performed in 10 patients. ILB was administered as a bolus dose of 1.5 mg/kg immediately prior to transplantation, followed by intraportal infusion of 3.0 mg/kg together with the islet transplant. A continuous ILB infusion was maintained during 5 hours after transplant with a total daily dose of up to 18.3 mg/kg. The primary endpoint was not fulfilled as ILB did not show superiority over heparin with regard to C-peptide response from the mixed-meal tolerance test. Thirty-one serious adverse events were reported during the study (including the pre-randomization period), but none of the SAEs appeared due to the study interventions. The results from this study show that administration of ILB is as safe and effective as the heparin standard therapy for allogenic islet transplantation.

1.2 Trial Rationale

1.2.1 Justification for patient population

HGF is a potent survival-promoting factor for motor neurons and has been suggested for ALS treatment[26, 27]. Human recombinant HGF (hrHGF) has been shown to attenuate spinal motor neuron degeneration in transgenic ALS rat models, and improve functional recovery in a non-human primate model of contusive cervical spinal cord injury[6, 28]. In a model of ALS in mice (SOD1 mutation), over-expression of HGF in the nervous system showed reduced motor neuron death and axonal degeneration as well as a prolonged life span[29]. HGF appears to be a good candidate for the treatment of ALS since it passes the blood-brain barrier (BBB)[30] and the HGF levels in ALS patients seem dysregulated[31]. ILB administration increases the plasma level of HGF in human healthy volunteers[8, 9, 32] and in rats and mice[33-35]. The levels in humans are increased >50-fold after intravenous administration of 3 mg/kg[8.] In TikoMed`s clinical and non-clinical studies, it has been shown that the mobilising effects of HGF is greater in man than in rodents[32, 35].

Other growth factors with potential therapeutic use in ALS are vascular endothelial growth factor (VEGF) and fibroblast growth factors (FGF). VEGF treatment increases survival, delays disease onset, improves motor functions, protects motor neurons and the neuromuscular junction in studies on rodent ALS models. VEGF is an attractive therapeutic factor since it passes the BBB and is down-regulated in the late stage of ALS[5]. FGF can protect motor neurons after axotomy-induced apoptosis and support neuronal regeneration[5]. Sulfated polysaccharides, like ILB, have been shown to bind and affect levels and potentiate VEGF and FGF signalling/effects. BDNF was one of the first neurotrophic factors discovered and demonstrated positive effects on motor neuron survival. BDNF has been administered in clinical trials in ALS patients. After administration of ILB a minor plasma concentration increase of BDNF has been documented in healthy volunteers[8, 36]. This demonstrates that ILB has an effect on a variety of growth factors and neurotrophic factors. The effect of ILB on growth factors has also been demonstrated in a gene array study, which also showed a potential for ILB to reduce glutamate signalling and oxidative stress which are both known factors of neurodegenerative diseases[3.]

ALS is a type of motor neurone disease that causes premature cell death of anterior horn cells and of motor neurons in the cerebral cortex. In an *in vitro* study on cultured human motor neurons and mouse cortical neurons ILB promoted the differentiation of neuronal cells within a very short period of time[2]. A gene expression study has been performed in 4 cell lines (HUVEC, Schwann cell, motor neuron and cortical cerebral neuronal cells). Using the gene expression data for `in silico` modelling, results indicate that ILB inhibits neuronal cell death, apoptosis, cell differentiation and homeostasis; and activates angiogenesis, migration of cells, cell viability, cell survival, proliferation of cells, cell cycle progression, cell transformation and expression of RNA. Under normal culture conditions the motor neurons and also cortical neurons appear to suffer from significant oxidative stress, leading to activation of some apoptotic

mechanisms as well as production of beta-amyloid in cells further exacerbating oxidative stress and mitochondrial fragmentation and oxidation of fatty acids. The addition of ILB to the cultures ameliorates these negative effects by preventing apoptosis, preventing beta-amyloid production and Lewy bodies, and subsequent damage by inhibiting fatty acid oxidation[3]. Furthermore, ALS is associated with aberrations in intracellular calcium levels, which could be mitigated by administration of ILB, as bradykinin is also a known regulator of intracellular calcium levels[37].

ILB administration in a model of multiple sclerosis has shown positive effects on disease progression[38, 39]. In a rat stroke model, ILB administration indicated improved motor function, which could be a neuro-protective effect and/or a result of increased angiogenesis[40]. Increased angiogenesis after ILB administration have been established in a number of hind-limb ischemia (HLI) studies on both mice and rats[41-44].

Several reports have demonstrated the reductions of blood flow and glucose metabolism in the cerebral cortex and the spinal cord in both ALS patients and the animal studies[45-48]. In an ALS model in mice, *in vivo* capillary imaging showed progressive decrease of capillary diameter, capillary density, and red blood cell speed during the disease course[49]. ILB is known to activate the Kinin-Kallikrein system which activates bradykinin through factor XII in the coagulation cascade. Bradykinin is a highly angiogenic peptide with a potential to restore blood flow in ischaemic tissues, an effect that has been well documented in several animal models of ischaemic diseases using ILB. In a series of studies it has been documented that ILB promotes increased angiogenesis selectively in ischemic/injured areas. This may contribute to a beneficial effect in ALS patients.

In conclusion, it is possible that the positive effect of ILB in ALS would be mediated through an increase of tissue factors. It is also possible that ILB has a direct effect on neuronal cells, as displayed *in vitro* with e.g. increased cell differentiation and decreased apoptosis. This effect might contribute to a beneficial effect in the treatment of neurological diseases including ALS and could be one mechanism behind the positive effects observed in MS and ischemic stroke models. In addition, an angiogenic effect may contribute to beneficial effects in ALS.as shown in a number of previous animal models[40-44].

1.2.2 Justification for design

We have proposed a single-arm design because it will allow us to quickly and effectively achieve the objectives of this trial. Specifically, it will allow us to assess the safety and tolerability of repeated doses of ILB and assess any early signs of potential efficacy.

1.2.3 Choice of treatment

Patients will receive 2mg/kg of ILB subcutaneously for ten weeks once per week in the first instance, during out-patient appointments at site.

Data from toxicity studies performed in mice, rats and monkeys, data from studies in healthy subjects and diabetes patients, and additional information obtained from the literature with similar substances, indicate that the proposed doses have a favourable safety profile.

In the two-part dose-finding study in healthy volunteers clinical study LMW-DS-102[8] using LMW-DS, dose proportionality of ILB was indicated by an apparent linear increase in C_{max} with increasing doses (3-24 mg/kg), and also AUC_{inf} appeared to increase linearly with increasing dose (see Table 1). Further,

SAMPLE

the pharmacokinetic parameters in study LMW-DS-102 correlated well with the results from other healthy volunteer studies, LMW-DS-101 (4.5 mg/kg)[7] and TM-104 (6 mg/kg, i.v. and s.c)[24]. Based on these results, it was considered reasonable to expect that the pharmacokinetics of ILB is approximately linear also at 2 mg/kg, and thus it could be anticipated that a dose of 2 mg/kg would after s.c. administration give a C_{max} around 5 mg/L and the AUC would be around 30 h*mg/L.

Table 1 Mean pharmacokinetic exposure parameters in plasma from healthy volunteers

Dose # (mg/kg)	C_{max} (mg/L)	AUC_{inf} (h*mg/L)	Treatment	Clinical Study No.
4.5	32.1	65.5	1.5 mg/kg, i.v. bolus and 3 mg/kg, 20 minutes i.v. infusion	**LMW-DS-101**
6	63.1	106	10 min i.v. infusion	**TM-104**
6	14.6	92	s.c.	
3	29.4	NC	10 minutes i.v. infusion	**LMW-DS-102**
6	70.0	154*		
9	76.8	156*		
12	120	264		
15	171	337		
18	159	330		
24	209	457		
Linear Extrapolation for 2 mg/ml				
2	***~5***	***~30***	***s.c.***	

\# PK results not fully comparable between i.v. studies, as different i.v. dosing schedules
Mean of n=3 or n=6 (24 mg/kg only) and n=12 (cross-over) in TM-104
NC: Not Calculated
*one subject

This is a first-in-patient study to carefully evaluate the safety and tolerability of ILB in ALS patients. Doses up to 24 mg/kg ILB has been administered to 79 healthy volunteers, but as ILB has never been administered to ALS patients and never more than two subsequent injections, a careful approach with low dose (2 mg/kg) with 1-week dosing interval has been chosen.

The vast majority of all cases of ALS are not caused by genetic factors, and for this population there are currently no animal models available. As a consequence, no *in vivo* pharmacology studies in ALS have been performed as a basis for this study. The rationale for the use of ILB in ALS is based on results from previous clinical trials and *in vitro* testing, and the dose and dosing interval is balanced to achieve an appropriate safety margin, as the safety of the ALS patients is here of utmost importance.

Production of several growth factors are dysregulated in ALS, and the study drug ILB has been shown to clearly increase the levels of growth factors such as HGF in both clinical and *in vivo* studies. In a dose-response study in healthy volunteers, where ILB was administered in a wide range of doses between 3-24 mg/kg, a similar increase (> 50-fold) in HGF levels was nonetheless reached, irrespective of the dose, within 30 minutes to 1 hour after administration (Figure 2). By extrapolating, it is anticipated that a significant increase in HGF levels will be reached with the dose of 2 mg/kg chosen for this study. Between 3-9 hours after ILB administration, the HGF levels drop in roughly a dose-dependent manner.

SAMPLE

Through its receptor c-MET, HGF activates the RAS-RAF pathway downstream, which in turn activates MAPK to translocate to the nucleus and activate transcription factors responsible for regulating a large number of genes[49]. That ILB has this effect on gene expression has been demonstrated through a gene array study, where the presence of ILB reduced expression of genes associated with oxidative stress, glutamate synthesis and apoptosis. As a similar increase in levels of HGF is achieved early after ILB administration, a similar effect on gene expression could also be reached, irrespective of the ILB dose administered.

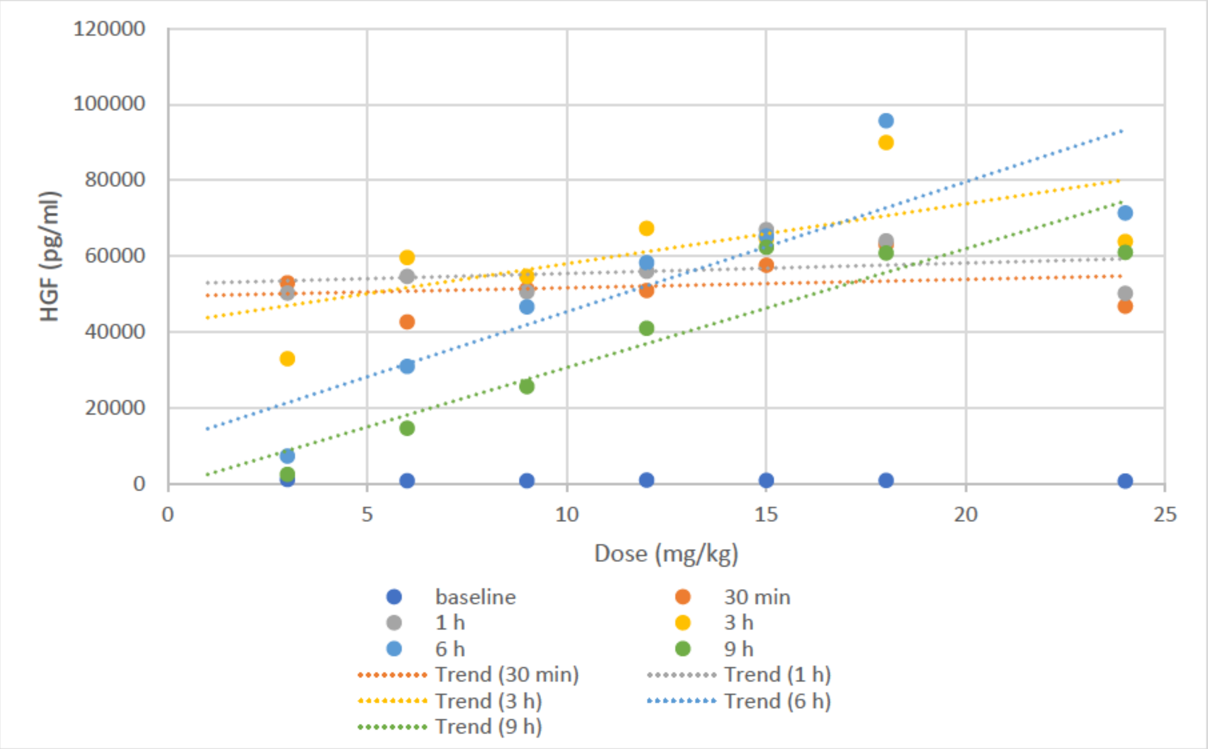

Figure 2 HGF levels at 30 min to 9 hours after administration of 3-24 mg/kg ILB in human (clinical study LMW-DS-102)

A similar increase (> 50-fold) in HGF levels is reached, irrespective of the dose, within 30 minutes to 1 hour after administration. By extrapolating, it is anticipated that a significant increase in HGF levels will be reached with the dose of 2 mg/kg chosen. Between 3-9 hours after ILB administration, the HGF levels drop in roughly a dose-dependent manner.

An intermittent dosing with 1-week interval has been chosen as a balance between a safe dose and an effective dose; As up to 24 mg/kg has been administered in man and toxicology studies show safe dosing of 15 mg/kg in rat and 30 mg/kg in monkey daily for 28 days, the 2 mg/kg dose with dosing once weekly provides a good safety margin (see also Section 5.4.2.3 in the updated IB, version 3.0). From an efficacy perspective, considering that the effect of ILB is on a gene level, there is reason to believe that even though the ILB *per se* is eliminated within less than 24 hours, the downstream effects may be more long lasting. Therefore, we propose a 1-week dosing interval in this first-in-patient study.

At this point, our view is that, for safety reasons, it is too soon to initiate a full dose-response study with ILB in ALS-patients. By starting at a low dose and infrequent dosing in a few ALS patients, this study will safely evaluate the tolerability of ILB, detect any early signs of potential efficacy on for instance biomarkers.

1.2.4 Justification for single point long-term remote follow up visit

A single point long-term remote follow up visit is planned for a time period of 8 weeks starting in Quarter 1 of 2021. Each patient will be asked to provide consent for this visit. Additional ALSFRS-R and Quality of Life data will be collected along with information on any medication each patient is taking for their ALS. The aim of this visit is to check on the wellbeing of participants and to increase scientific impact of the study which was terminated early as a result of the ongoing COVID-19 pandemic in 2020. The additional data will be analysed and provided in an addendum to the final report of the study results.

Patients with MND of any type are at a higher risk of developing severe complications and dying following COVID-19 infection[50]. As the study requires frequent visits to hospital, specifically for treatment, a risk/benefit analysis was completed by the study management team (March 2020) which concluded that that whilst the COVID-19 pandemic was prevalent in the United Kingdom currently recruited patients would receive no further treatment and recruitment of additional patients would be suspended. In October 2020 it was decided that due to the ongoing situation with regards to COVID-19 pandemic in the United Kingdom further treatment and recruitment of additional patients would be permanently ended. This study remains valid and in order to maximise the scientific value of the study and the clinical potential of the data collected, one additional follow–up visit to collect some secondary outcome data (as detailed above) was deemed to be substantially beneficial for developing the intervention for future patients whilst minimising the impact on the patients who have participated in this clinical study.

SAMPLE

2. OBJECTIVES AND OUTCOME MEASURES

2.1 Primary Objective

- To determine the safety and tolerability of ILB administered subcutaneously weekly in ALS patients.

2.2 Secondary Objectives

To describe the effect of ILB administered subcutaneously weekly

- upon severity of symptoms associated with ALS present at entry into the study
- upon development of new symptoms associated with ALS during the study
- upon patients quality of life (ALSAQ-40) during the study

To assess changes in biomarkers, including urinary p75^{ECD} and NfL in plasma

2.3 Primary Outcome Measures

Our primary outcome measures will inform whether the therapy is feasible in this patient group.

- **Safety**
  - Measured by the incidence of serious adverse events (SAEs) and adverse events (AEs) using CTCAE v4.0. Events will be summarised by grade, relatedness, admitting event (for SAEs); expectedness and sequelae.
- **Tolerability**
  - Measured by the incidence of *intolerable adverse events*. An intolerable adverse event will satisfy all of the following criteria:

    1. Associated with a serious adverse event or a drug discontinuation of greater than three weeks;

    2. Grade 3, 4 or 5 in severity according to CTCAE version 4;

    3. In the opinion of the Investigator is i) definitely related or ii) probably related or iii) possibly related to the study drug treatment.
  - Adverse events which are considered unrelated or probably not related will not be classed as intolerable events.
- **Quantity of study drug administered**
  - Total drug administered, number of administrations, number and length of interruptions, and number of discontinuations will be reported.

2.4 Secondary Outcome Measures

Our secondary outcome measures will provide data on patient function and disease progression

- Revised ALS Functional Rating Scale (ALSFRS-R)
  - This is a functional rating scale, including assessments of communication, mobility, feeding, dressing and respiration.
- ALS Assessment Questionnaire (ALS AQ-40)
  - This patient-reported outcome measures the subjective well-being of patients. It is broader than ALSFRS-R and adds assessment of emotional reactions.
- Urinary p75^{ECD}
  - This is a biological fluid-based biomarker of ALS disease progression
- PK ILB in plasma following administration
  - This quantifies the amount of drug detectable in the blood after administration over time
- NfL in plasma
  - This is a blood-based biomarker for neurodegeneration

SAMPLE

2.5 Exploratory Outcome Measures

- HPLC analyses of purine-pyrimidine metabolites (serum)
- HPLC analysis of fat-soluble vitamins and antioxidants (serum)
- HPLC analyses of amino acids (AA) and amino-group containing compounds (ACCG) (serum)
- Spectrophotometric analysis of lactate
- PBMC samples will be collected for analysis of change in phenotypic balance
- HGF release in plasma following ILB administration (ELISA from PK samples)
- Creatine kinase and Myoglobin from plasma as markers of muscle atrophy associated with ALS

SAMPLE

3. TRIAL DESIGN

This is a Phase IIa, pilot, single arm (uncontrolled) study, which seeks to recruit 15 patients with a diagnosis of ALS. The trial has no control arm because control outcomes will not address the primary objective of the trial. On recruitment to the study, patients will undergo a baseline screening assessment (within 14 days of initiation of treatment with ILB, or within 28 days for those patients discontinuing Riluzole at screening) to include a medical history, vital signs, haematology and blood biochemistry (laboratory assessments). Patients will also complete a baseline assessment on the ALS Functional Rating Scale (ALSFRS-R) and a Quality of Life Assessment (ALSAQ-40).

Patients will receive 2mg/kg of ILB subcutaneously, weekly for ten weeks (in the first instance), during out-patient visits at site. Dosing beyond 10 weeks (initially to 24 weeks and a maximum of up to 48 weeks) is dependent upon a formal review of the patient's eligibility, their wishes and the most suitable treatment options. If treatment extension is agreed (at the PIs discretion), patients will initially go on to complete an additional 14 weeks of treatment, with the option to extend treatment further. While patients are on the treatment extension phase of the trial a formal review will occur every 90 days.

During the clinical trial, regular assessments of adverse events, vital signs, haematology and blood biochemistry (laboratory assessments), ALSFRS-R and Quality of Life (ALSAQ-40) will be performed. These will be scheduled to occur weekly (during the main trial phase) and monthly thereafter in follow-up and/or treatment extension phase as per the schedule of events.

Following the suspension of treatment in March 2020 and the early termination of the study in October 2020 due to the COVID-19 pandemic, as well as the high risk status of participants, a single point long-term remote follow up visit will take place starting in Quarter 1 of 2021.

Patients will be asked to re-consent for this additional visit and data capture which will be conducted by the site PI via video call over an 8 week period.

During this remote visit, data collected will include ALSFRS-R, Quality of Life (ALSAQ-40) and concomitant medication – patients will be asked to provide information on any medication they have been taking for their condition, such as standard of care Riluzole.

SAMPLE

4. ELIGIBILITY

4.1 Main Eligibility criteria

Inclusion Criteria:

1. Patients ≥18 years and who have provided written informed consent to participate in the study
2. Prior to trial entry patients will have a *definite* diagnosis of ALS according to El Escorial Criteria (Appendix 10). All patients will demonstrate either:
    - **presence of Upper Motor Neuron (UMN)** (increased tone, brisk reflexes) **as well as Lower Motor Neuron (LMN)** (weakness, wasting and fasciculation) **signs in the bulbar region and at least two of the other spinal regions** (cervical, thoracic or lumbosacral)

      or

    - **presence of UMN and LMN signs in all three spinal regions** (cervical, thoracic or lumbosacral)
3. Electrophysiological tests (Electromyography (EMG) / Nerve Conduction Study (NCS)) that supports the diagnosis of Motor Neurone Disease (MND) and to exclude mimic disorders
4. Forced Vital Capacity (FVC) ≥50% of predicted value for gender, height and age at screening and/or a mean Sniff Nasal Inspiratory Pressure (SNIP) ≥50% of predicted value for age
5. Adequate haematological function (Hb≥10g/dl absolute neutrophil count ≥1.5x10^9/L and a platelet count ≥60 x10^9/L
6. International Normalised Ratio (INR) ≤ 1.5, Activated Partial Thromboplastin Time (aPTT) ≤ 40 seconds, Prothrombin Time (PT) ≤ 13.5 seconds
7. Patient willing and able to comply with schedule visits, treatment plan and other study procedures.
8. Patients taking Riluzole must have discontinued treatment ≥28 days prior to study entry (and following consent to take part in the study)
9. Women Of Child Bearing Potential (WOCBP) who agree to use highly effective means of contraception (as defined in the Heads of Medicines Agencies_Clinical Trials Facilitation Group (HMA_CTFG) guideline (see Appendix 8) and in combination with a barrier contraception method (condom, diaphragm or cap) for the entirety of the study

SAMPLE

Exclusion Criteria:

1. Patients classified as either probable or possible ALS according to El Escorial Criteria (see Appendix 10).
2. Subjects in whom other causes of neuromuscular weakness have not been excluded
3. Assisted ventilation of any type within 3 months before the screening visit or at screening
4 Patients requiring Radiologically Inserted Gastrostomy (RIG) or Percutaneous Endoscopic Gastroscopy (PEG) feeding
5. Involvement in any other interventional study involving use of another IMP or biological product, within 3 months of screening

6. Any use of antioxidants, edaravone, tirasemtiv or CK-2127107 within 1 month before the screening visit
7. Any botulinum toxin use within 3 months before the screening visit.
8. Any form of stem cell or gene therapy for the treatment of amyotrophic lateral sclerosis (ALS)
9. Neuroimaging of brain and cervical spine with Magnetic Resonance imaging (MRI) indicating compressive myelopathy as an alternate diagnosis
10. Laboratory examinations including Acetylcholine receptor (AChR) antibodies and Muscle Specific Kinase (MuSK) antibodies to exclude Bulbar onset Myasthenia gravis from Bulbar onset Motor neuron disease as an alternate diagnosis and Antinuclear Antibodies (ANA), Anti-neutrophil cytoplasmic antibodies (ANCA), Extractable Nuclear Antigen (ENA) antibodies, Creatine Kinase (CK), electrophoresis and immunoglobulin indicating an alternate diagnosis for muscle disease like Myositis. It is at the Chief Investigators discretion to determine if positive results from any of the mentioned tests indicates an alternative diagnosis.
11. Abnormal liver function defined as Aspartate Transaminase (AST) and/or Alanine Transaminase (ALT) >3 times upper limit of normal
12. Any head trauma, intracranial or spinal surgery within 3 months of trial entry
13. Patients who have had recurrent falls will be excluded to reduce the risk of intracerebral haemorrhage with this IMP
14. Current use of an anticoagulant e.g Warfarin, Aspirin, Clopidogrel, any novel anticoagulants (NOAC)s or low molecular weight subcutaneous heparin
15. Uncontrolled severe hypertension defined as systolic blood pressure (SBP) ≥ 220 mmHg or diastolic blood pressure (DBP) ≥120 mmHg
16. Current or previous history of heparin-induced thrombocytopenia
17. Active peptic ulcer disease
18. Known hypersensitivity to sulphur
19. Severe liver insufficiency
20. Patients with evidence of major psychiatric illness, significant cognitive impairment or clinically evident dementia that may interfere with the patients' ability to comply with study procedures
21. Pulmonary illness (e.g. asthma or Chronic Obstructive Pulmonary Disease (COPD)) requiring regular treatment
22. Patient judged to be actively suicidal by the investigator during 3 months before the screening visit
23. Subjects with a diagnosis of another neurodegenerative disease (e.g. Parkinson`s disease, Alzheimer's disease and Frontotemporal dementia
24. Pregnant and/or breastfeeding

SAMPLE

4.2 Treatment Extension Eligibility Criteria

The following must be met for patients to be considered for treatment extension beyond 10 weeks:

Inclusion:

1. Completed 10 weeks of treatment
2. Favourable toxicity profile, as reviewed by the PI
3. Forced Vital Capacity (FVC) ≥50% of predicted value for gender, height and age at screening and/or a mean Sniff Nasal Inspiratory Pressure (SNIP) ≥50% of predicted value for age
4. Patient willing to continue

Exclusion:

1. Meeting any of the patient stopping rules (see section 7.1.6)
2. Pregnancy/breast feeding
3. Patients who have had recurrent falls will be excluded to reduce the risk of intracerebral haemorrhage with this Investigational Medicinal Product (IMP)
4. Patients requiring Radiologically Inserted Gastrostomy (RIG) or Percutaneous Endoscopic Gastroscopy (PEG) feeding
5. use of an anticoagulant e.g Warfarin, Aspirin, Clopidogrel, any novel anticoagulants (NOAC)s or low molecular weight subcutaneous heparin
6. Worsening function status and unable to attend weekly out-patient appointments.

SAMPLE

5. CONSENT AND SCREENING

5.1 Informed Consent

It is the responsibility of the Investigator (detailed in the site delegation log) to obtain written informed consent for each patient prior to performing any trial related procedure. A Patient Information Sheet (PIS) is provided to facilitate this process. Investigators must ensure that they adequately explain the aim, trial treatment, anticipated benefits and potential hazards of taking part in the trial to the patient. The Investigator should also stress that the patient is completely free to refuse to take part or withdraw from the trial at any time. The patient should be given ample time (a minimum of 24 hours) to read the Patient Information Sheet and to discuss their participation with others outside of the site research team. The patient must be given an opportunity to ask questions which should be answered to their satisfaction. The right of the patient to refuse to participate in the trial without giving a reason must be respected.

If the patient expresses an interest in participating in the trial they should be asked to sign and date the latest version of the Informed Consent Form (ICF). The Investigator (detailed in the site delegation log) must then sign and date the form. A copy of the ICF should be given to the patient, a copy should be filed in the hospital notes, and the original placed in the Investigator Site File (ISF). Once the patient is entered into the trial the patient's trial number should be entered on the Informed Consent Form maintained in the ISF. *In addition, if the patient has given explicit consent a copy of the signed Informed Consent Form must be sent in the post to the Trials Office for review.*

Details of the informed consent discussions should be recorded in the patient's medical notes, this should include date of, and information regarding, the initial discussion, the date consent was given, with the name of the trial and the version number of the Patient Information Sheet and Informed Consent Form. Throughout the trial the patient should have the opportunity to ask questions about the trial and any new information that may be relevant to the patient's continued participation should be shared with them in a timely manner. On occasion it may be necessary to re-consent the patient in which case the process above should be followed and the patient's right to withdraw from the trial respected.

Electronic copies of the Patient Information Sheet and Informed Consent Form are available from the Trials Office and should be printed or photocopied onto the headed paper of the local institution.

Details of all patients approached about the trial should be recorded on the Patient Screening/Enrolment Log and with the patient's prior consent their General Practitioner (GP) should also be informed that they are taking part in the trial. A GP Letter is provided electronically for this purpose.

5.1.1 Treatment Extension

If patients are eligible to continue treatment after week 10 they will receive an additional patient information sheet which will detail the treatment extension phase of the trial. Patients will be asked to sign and date an additional informed consent form to confirm they agree to continue treatment beyond week 10.

SAMPLE

5.1.2 Single Point Long-Term Remote Follow Up

Electronic consent will be taken for the single point long-term remote follow up visit. Electronic PIS will be supplied to the participants prior to the visit. Consent will be taken at the beginning of the video visit by use of an electronic consent form that the patient will complete and return to the clinician. In this scenario, a simple typewritten e-signature will be sufficient.

5.2 Screening

Prior to the formal screening process patients will be required to provide written informed consent **BEFORE** any non-standard of care assessment can be performed, as detailed in section 5.1. It is anticipated that the majority of the screening process will be completed at the same visit (i.e. all blood tests performed on the same day).

Each patient who has consented, and enters the screening process, will be allocated a registration number. The site will be provided with a Patient Screening/Enrolment log, which is pre-populated with site specific screening numbers. Please use this number in any correspondence with the ALS Trial Office. If the patient is eligible for the trial, the screening number should be completed on the Registration Form. A Trial Number will be allocated once the patient is registered.

Screening Visit (within 14 days of registration) or (within 28 days for those patients taking Riluzole prior to trial entry)

Visit 1

- Informed consent (patients on Riluzole must discontinue treatment at this visit)
- Medical history*
- Amyotrophic Lateral Sclerosis Functional Rating Scale (revised) (ALSFRS-R)
- Eligibility Assessment
- Concomitant drug history
- Vital signs (blood pressure, heart rate, body temperature, pulse oximetry and respiration rate). Results of pulse oximetry should be the average of three recordings. Body weight, height and Body Mass Index (BMI)
- Electrocardiogram (ECG)
- Lung Function Tests (Forced Vital Capacity (FVC))≥50% of predicted value for gender, height and age at screening
- Sniff Nasal Inspiratory Pressure (SNIP) Results must be recorded as the mean of three recordings Haematology tests (to include Full Blood Count (FBC), Erythrocyte Sedimentation Rate (ESR)
- Coagulation tests (to include INR, APTT and PT)
- Biochemistry tests (to include, Troponin I, C-reactive protein (CRP), Glucose, Calcium
- Serum Creatine Kinase – Muscle, Brain (CK-MB)
- Urea and Electrolytes (U&Es), Liver Function Tests (LFTs)
- Thyroid Stimulating Hormone (TSH), Free Thyroxine (Free T4)
- Immunoglobulins (IgG, IgM)
- Quality of Life questionnaire(ALSAQ-40)
- Pregnancy test for WOCBP

***to include an assessment of any mental or physical contraindications to study entry**

SAMPLE

6. TRIAL ENTRY

6.1 Registration

After the results of the screening visit are available the following must be verified by the local site PI and/or Co-investigators before registering the patient onto the trial:

- Patient`s ICF
- Confirmation of all the inclusion criteria
- Review of all the exclusion criteria
- Completion of Eligibility Checklist & Registration Form

All of the above must be completed before proceeding onto patient registration.

Sentinel patients that are eligible for inclusion in the trial and have signed an ICF will be registered via the ALS Trials Office. Informed consent must be obtained prior to registration. An Eligibility Checklist and Registration Form must be completed and faxed to the ALS Trial Office.

Registration line:

TEL: +44 (0) 121 371 8492 (09:00 – 17:00, Monday-Friday)

The patient's eligibility will be confirmed at registration by the ALS Trial Office. If eligible for the trial, the patient will be allocated a unique Trial Number.

Following recruitment of the first two sentinel patients all other patients that are eligible for inclusion in the trial and have signed an ICF will be registered via the online electronic remote data capture system (eRDC). Informed consent must be obtained prior to registration. A paper Eligibility Checklist must also be completed and faxed to the ALS Trials Office at the time of registration. The registration line will be open during office hours (09:00 – 17:00, Monday-Friday).

Registration of patients can be achieved by logging on to:

https://www.cancertrials.bham.ac.uk

Full details will be provided to sites separately.

The schedule for investigations and follow up visits is summarised in the Schedule of Assessments table.

A copy of the patients ICF must be sent in the post to the ALS Trial Office, if the patient has given explicit consent for this.

At this time point, patients will be allocated a unique trial number to preserve patient confidentiality.

Trial drug prescriptions must include the patient's Trial Number.

6.1.1 Emergency Registration

In case of any problems with on-line registration (eRDC) system a paper eligibility checklist and registration form should be completed. Site staff should then telephone the ALS Trial Office using the

SAMPLE

following telephone numbers listed below where the registration falls within office hours. If a registration falls outside of office hours and there is a problem with the online system the registration will not be able to be completed until the trial office opens.

Contact details for emergency registration:

Tel: +44 (0) 121 371 8492 (09.00 – 17.00, Mon-Fri)
Email: ALS@trials.bham.ac.uk
(include in subject header "emergency randomisation)

A unique trial number may not be allocated at the time of emergency registration; this will be allocated by the ALS Trial Office as soon as possible afterwards.

6.1.2 Protocol waivers or exemptions

Protocol waivers or exemptions are not allowed with the exception of immediate safety concerns. Therefore, adherence to the study design requirements, including those specified in the schedule of events table, and other sections of the study protocol is mandatory.

6.1.3 Treatment Extension registration

If patients are eligible and agree to continue treatment beyond 10 weeks, the following will be completed:

- Patient complete treatment continuation ICF
- Confirmation of the inclusion criteria
- Review of the exclusion criteria
- Completion of Treatment Extension registration form

A copy of the patients ICF must be sent to the ALS Trial Office, if the patient has given explicit consent for this. A treatment continuation form will be completed, stating how many/if any weeks the patient has not received treatment between the end of the initial 10 week treatment stage and the beginning of the treatment extension phase.

SAMPLE

7. TREATMENT DETAILS

7.1 Investigational Medicinal Product

The Investigational Medicinal Product (IMP) ILB is a type of low molecular weight dextran sulfate (LMW-DS).

7.1.1 Dosage, Form and Composition

The IMP contains 100mg/ml ILB in 0.9% NaCl and is a sterile, yellow-brownish solution for subcutaneous injection. The active pharmaceutical ingredient is a type of low molecular weight dextran sulfate (LMW-DS).

The IMP was manufactured according to current Good Manufacturing Practice by APL in Umeå, Sweden to meet all requirements for parenteral solutions:

Apotek Produktion & Laboratorier AB (APL)
Formvägen 5B
SE-906 04 UMEÅ
Sweden

The analytical testing of the IMP is mainly performed by APL, but parts of the release testing (average molecular weight and free sulfate content) were conducted at:

Eurofins Biopharma Product Testing Sweden
Seminariegatan 29
SE-752 28 Uppsala

Patients will receive 2mg/kg of ILB subcutaneously for ten weeks (in the first instance) once per week.

7.1.2 Packaging and Labelling

The IMP is filled in a 2.9ml glass vial sealed with a rubber stopper and a tear off aluminium cap. The vials are provided in paper boxes and there will be 36 x 2.9ml glass vials in each box. Each vial will be labelled in accordance with local law, all applicable regulatory requirements and Good Clinical Practice (GCP).

7.1.3 Storage and handling

The ILB vials must be stored at +2 to +8°C, and be in room temperature no more than one hour prior to administration. The IMP ILB should be administered as specified in the protocol.

Further handling details can be found in the current version of the ALS Pharmacy Manual

SAMPLE

7.1.4 Trial Treatment

This clinical trial is a single-arm safety and tolerability study therefore all patients registered onto the trial will receive the same treatment as detailed below:

Treatment Group: All patients will receive ILB. Administration will be weekly subcutaneous injections at a dose of 2mg/kg once per week for 10 weeks (in the first instance).

7.1.5 Continuation of treatment beyond 10 weeks (initially to 24 weeks up to a maximum of 48 weeks)

See Schedule of Assessments (2): Treatment extension. All patients will be reviewed for eligibility to continue treatment beyond 10 weeks by the PI.

7.1.6 Patient Stopping Rules

Patients will have treatment of ILB stopped if any of the following prospective criteria are met **during the treatment period**:

- The patient experiences a Serious Adverse Event related to IMP
- If there is a decrease in ALSFRS-R score (>50%) compared to baseline
- If a patient becomes pregnant during the study treatment period
- Unacceptable toxicity – any adverse event which, in the investigators opinion, requires termination of the study drug
- Unforeseen events – any event which in the judgement of the Investigator makes further treatment inadvisable
- SAE requiring discontinuation of treatment
- If a patient requires an anticoagulant e.g Warfarin, Aspirin, Clopidogrel, any novel anticoagulants (NOAC)s or low molecular weight subcutaneous heparin during the clinical trial
- PTT or aPTT > 1.5 x ULN
- INR > 1.8 x ULN

Patients, who stop weekly dosing, will be invited to remain in the observational part of the study up to 24 weeks, to enable assessment of safety related measures. Patients that discontinue ILB and subsequently start or re-commence Riluzole therapy will also be invited to remain in the observational part of the study up to 24 weeks.

7.1.7 Rescue Medication

See protocol page (ix)

7.1.8 Special Warnings and Special Precautions for use

The early clinical studies show that short-term treatment with ILB is well tolerated. However, prolonged treatment may lead to more serious adverse events. ILB prolongs APTT in a dose-dependent manner, and after prolonged treatment in humans with a similar compound, but with higher molecular weight, thrombocytopenia has been observed. Both these effects might lead to bleeding complications.

A hypersensitivity reaction may occur as reported in a previous study.

SAMPLE

7.2 Treatment Schedule

The following treatment schedule is based on an initial 10 week schedule of drug administration with the option to continue to 24 weeks in the first instance.

Treatment Week 1 – Day 1 (not <28 days prior to last dose of Riluzole for those on treatment) Visit 2

- Eligibility Assessment
- ALSFRS-R
- Registration (must not be <28 days prior to last dose of Riluzole for those on treatment)
- Concomitant drug history
- Vital signs
- Haematology tests (to include Full Blood Count (FBC), Erythrocyte Sedimentation Rate (ESR)
- Coagulation Tests (to include INR, APTT and PT)
- Biochemistry tests (to include Troponin I, C-reactive protein (CRP), Glucose, Calcium
- Urea and Electrolytes (U&Es), Liver Function Tests (LFTs)
- Quality of Life questionnaire (ALSAQ-40)
- Urinary $p75^{ECD}$
- Neurofilament Light Chain (NfL) levels (collect 10mls of whole blood to allow for 4-5mls plasma for analysis)
- Collection of exploratory biomarker blood samples
- PK Testing
- **Study Drug administration** (the time of study drug administration must be recorded)
- Adverse event/Toxicity reporting
- Dynamometer test

SAMPLE

Treatment Week 2-4
(Visits 3, 4 and 5)

- ALSFRS-R
- Concomitant drug history
- Vital signs
- Haematology tests (to include Full Blood Count (FBC), Erythrocyte Sedimentation Rate (ESR)
- Coagulation Tests (to include INR, APTT and PT)
- Biochemistry tests (to include Troponin I, C-reactive protein (CRP), Glucose, Calcium
- Urea and Electrolytes (U&Es), Liver Function Tests (LFTs)
- Quality of Life questionnaire (ALSAQ-40)
- Pregnancy test for WOCBP (Week 4, day 22 only)
- **Study Drug administration** (The time of study drug administration will be recorded at each visit. All doses must be given at within +/- 24 hours of the Day 1 Week 1 administration time
- Adverse event/Toxicity reporting
- Dynamometer test

Treatment Week 5
(Visit 6)

- ALSFRS-R
- Concomitant drug history

- Vital signs
- ECG
- Haematology tests (to include Full Blood Count (FBC), Erythrocyte Sedimentation Rate (ESR)
- Coagulation tests (INR, APTT and PT)
- Biochemistry tests (to include Troponin I, C-reactive protein (CRP), Glucose, Calcium
- Serum Creatine Kinase – Muscle, Brain (CK-MB)
- Urea and Electrolytes (U&Es), Liver Function Tests (LFTs)
- Quality of Life questionnaire(ALSAQ-40)
- Urinary $p75^{ECD}$ levels
- Neurofilament Light Chain (NfL) levels (collect 10mls of whole blood to allow for 4-5mls plasma for analysis)
- Collection of Exploratory Biomarker Blood Samples
- Lung Function Tests (Forced Vital Capacity (FVC))≥50% of predicted value for gender, height and age at screening
- Sniff Nasal Inspiratory Pressure (SNIP) Results must be recorded as the mean of three recordings Haematology tests (to include Full Blood Count (FBC), Erythrocyte Sedimentation Rate (ESR)
- **Study Drug administration** (The time of study drug administration will be recorded at each visit. All doses must be given within +/- 24 hours of the Day 1 Week 1 administration time
- Adverse event/Toxicity reporting
- Dynamometer test

Treatment Week 6, 7, 8

(Visit 7, 8 and 9)

- ALSFRS-R
- Concomitant drug history
- Vital signs
- Haematology tests (to include Full Blood Count (FBC), Erythrocyte Sedimentation Rate (ESR)
- Coagulation tests (to include INR, APTT and PT)
- Biochemistry tests (to include: Troponin I, C-reactive protein (CRP), Glucose, Calcium
- Urea and Electrolytes (U&Es), Liver Function Tests (LFTs)
- Quality of Life questionnaire(ALSAQ-40)
- **Pregnancy test for WOCBP (Week 8, day 50 only)**
- **Study Drug administration** (The time of study drug administration will be recorded at each visit. All doses must be given within +/- 24 hours of the Day 1 Week 1 administration time Adverse event/Toxicity reporting
- Dynamometer test

Treatment Week 9

(Visit 10)

- ALSFRS-R
- Concomitant drug history
- Vital signs
- Lung Function Tests (FVC)
- SNIP test
- Haematology tests (to include Full Blood Count (FBC), Erythrocyte Sedimentation Rate (ESR)
- Coagulation tests (to include INR, APTT and PT)
- Biochemistry tests (to include: Troponin I, C-reactive protein (CRP), Glucose, Calcium
- Urea and Electrolytes (U&Es), Liver Function Tests (LFTs)
- Quality of Life questionnaire(ALSAQ-40)
- Dynamometer test

Treatment Week 10

(Visit 11)

- ALSFRS-R
- Concomitant drug history
- Vital signs
- ECG
- Haematology tests (to include Full Blood Count (FBC), Erythrocyte Sedimentation Rate (ESR)
- Coagulation tests (to include INR, APTT and PT)
- Biochemistry tests (to include: Troponin I, C-reactive protein (CRP), Glucose, Calcium
- Creatinine Kinase
- Urea and Electrolytes (U&Es), Liver Function Tests (LFTs)
- Thyroid function tests
- Immunoglobulins
- Quality of Life questionnaire(ALSAQ-40)
- Urinary $p75^{ECD}$ levels
- Neurofilament Light Chain (NfL) levels (collect 10mls of whole blood to allow for 4-5mls plasma for analysis)
- Collection of exploratory biomarker blood samples
- Pregnancy test for WOCBP
- Dynamometer test
- **Study Drug administration** (The time of study drug administration will be recorded at each visit. All doses must be given within +/- 24 hours of the Day 1 Week 1 administration time

Prior to Week 11

- **A formal review will be conducted to discuss patient eligibility to determine whether to continue treatment beyond 10 weeks. If a patient consents to continued treatment they will follow the below continuation of treatment visits. If a patient is not eligible/ does not want to continue treatment they will follow the follow up (observation period) schedule.**

Treatment Week 11

- Re-consent
- ALSFRS-R
- Concomitant drug history
- **Study Drug administration** (The time of study drug administration will be recorded at each visit. All doses must be given within +/- 24 hours of the Day 1 Week 1 administration time
- Adverse event/Toxicity reporting

Treatment Week 12-24

- Concomitant drug history
- **Study Drug administration** (The time of study drug administration will be recorded at each visit. All doses must be given within +/- 24 hours of the Day 1 Week 1 administration time
- Adverse event/Toxicity reporting

Treatment Week 14

- ALSFRS-R
- Concomitant drug history
- Vital signs
- ECG
- Haematology tests (to include Full Blood Count (FBC), Erythrocyte Sedimentation Rate (ESR)
- Coagulation tests (INR, APTT and PT)
- Biochemistry tests (to include Troponin I, C-reactive protein (CRP), Glucose, Calcium
- Serum Creatine Kinase – Muscle, Brain (CK-MB)
- Urea and Electrolytes (U&Es), Liver Function Tests (LFTs)
- Quality of Life questionnaire(ALSAQ-40)
- Lung Function Tests (Forced Vital Capacity (FVC))≥50% of predicted value for gender, height and age at screening
- Sniff Nasal Inspiratory Pressure (SNIP) Results must be recorded as the mean of three recordings Haematology tests (to include Full Blood Count (FBC), Erythrocyte Sedimentation Rate (ESR)
- Pregnancy test for WOCBP
- **Study Drug administration** (The time of study drug administration will be recorded at each visit. All doses must be given within +/- 24 hours of the Day 1 Week 1 administration time Adverse event/Toxicity reporting
- Dynamometer test

Treatment Week 15, 16,17 and 18

- Concomitant drug history
- **Study Drug administration** (The time of study drug administration will be recorded at each visit. All doses must be given within +/- 24 hours of the Day 1 Week 1 administration time Adverse event/Toxicity reporting

SAMPLE

Treatment Week 19

- ALSFRS-R
- Concomitant drug history
- Vital signs
- ECG
- Haematology tests (to include Full Blood Count (FBC), Erythrocyte Sedimentation Rate (ESR)
- Coagulation tests (INR, APTT and PT)
- Biochemistry tests (to include Troponin I, C-reactive protein (CRP), Glucose, Calcium
- Serum Creatine Kinase – Muscle, Brain (CK-MB)
- Urea and Electrolytes (U&Es), Liver Function Tests (LFTs)
- Quality of Life questionnaire(ALSAQ-40)
- Lung Function Tests (Forced Vital Capacity (FVC))≥50% of predicted value for gender, height and age at screening
- Sniff Nasal Inspiratory Pressure (SNIP) Results must be recorded as the mean of three recordings Haematology tests (to include Full Blood Count (FBC), Erythrocyte Sedimentation Rate (ESR)
- Pregnancy test for WOCBP
- **Study Drug administration** (The time of study drug administration will be recorded at each visit. All doses must be given within +/- 24 hours of the Day 1 Week 1 administration time Adverse event/Toxicity reporting
- Dynamometer test

Treatment Week 20-23

- Concomitant drug history
- **Study Drug administration** (The time of study drug administration will be recorded at each visit. All doses must be given within +/- 24 hours of the Day 1 Week 1 administration time Adverse event/Toxicity reporting

Treatment Week 24

- ALSFRS-R
- Concomitant drug history
- Vital signs
- ECG
- Haematology tests (to include Full Blood Count (FBC), Erythrocyte Sedimentation Rate (ESR)
- Coagulation tests (INR, APTT and PT)
- Biochemistry tests (to include Troponin I, C-reactive protein (CRP), Glucose, Calcium
- Serum Creatine Kinase – Muscle, Brain (CK-MB)
- Urea and Electrolytes (U&Es), Liver Function Tests (LFTs)
- Quality of Life questionnaire(ALSAQ-40)
- Urinary $p75^{ECD}$ levels
- Neurofilament Light Chain (NfL) levels (collect 10mls of whole blood to allow for 4-5mls plasma for analysis)
- Collection of Exploratory Biomarker Blood Samples
- Lung Function Tests (Forced Vital Capacity (FVC))≥50% of predicted value for gender, height and age at screening
- Sniff Nasal Inspiratory Pressure (SNIP) Results must be recorded as the mean of three recordings Haematology tests (to include Full Blood Count (FBC), Erythrocyte Sedimentation Rate (ESR)

SAMPLE

- Pregnancy test for WOCBP
- **Study Drug administration** (The time of study drug administration will be recorded at each visit. All doses must be given within +/- 24 hours of the Day 1 Week 1 administration time Adverse event/Toxicity reporting
- Dynamometer test

If after review, patients are eligible to continue treatment beyond 24 weeks up to a maximum of 48 weeks, they will repeat the treatment and assessment schedule week 11 – 24.

SAMPLE

7.3 Assessments

7.3.1 Blood chemistry and Haematology

The following blood tests will be performed at each visit. All safety assessments will be done in local laboratories at the investigative site.

Biochemistry	Blood test
U&Es	Sodium<br>Potassium<br>Urea<br>Creatinine<br>*Estimated Glomerular Filtration Rate (eGFR) (calculated by Cockcroft-Gault equation or alternative as per institutional practice – Appendix 6)
LFTs	Albumin<br>Aspartate transaminase (AST)<br>Alanine Transaminase (ALT)<br>Alkaline Phosphatase (ALP)<br>Bilirubin (direct and indirect if total is ≥30)<br>Gamma Glutamyl Transpeptidase (GGT)
Other	Serum Creatine Kinase-MB (CK-MB)<br>Troponin I (not tested after treatment week 24)<br>C-reactive protein (CRP)<br>Glucose<br>Calcium<br>Thyroid Stimulating Hormone (TSH) (not tested after treatment week 24)<br>Free Thyroxine (Free T4) (not tested after treatment week 24)<br>Immunoglobulin G (IgG)<br>Immunoglobulin M (IgM)
**Haematology**	**Blood test**
FBC	Haemoglobin<br>Platelets<br>Red Blood Cells<br>White Blood Cells<br>Haematocrit<br>Mean Cell Volume (MCV)<br>Mean Cell Haemoglobin<br>Neutrophils<br>Lymphocytes<br>Monocytes<br>Eosinophils<br>Basophils
Other	ESR
Coagulation Tests	INR<br>APTT<br>PT

7.3.1.1 Liver Chemistry Management

There are no liver-specific stopping criteria as such (although there are opportunities for an independent committee to review general safety data and, if necessary, advise the trial be stopped. In the event of

liver parameters deviating from the thresholds described in Appendix 5, follow instructions in that section.

Liver chemistry increased monitoring criteria have been designed to assure subject safety and evaluate liver event aetiology (in alignment with the FDA premarketing clinical liver safety guidance). http://www.fda.gov/downloads/Drugs/GuidanceComplianceRegulatoryInformation/Guidances/UCM174090.pdf

7.3.2 Physical examination/symptom assessment

All physical assessments will be performed by a suitability qualified medical practitioner/research nurse PRIOR TO ADMINISTRATION OF STUDY DRUG and shall include:

- Blood pressure
- Heart rate
- Pulse oximetry. Results of oximetry should be the average of three recordings
- Weight (kg) to allow for accurate dosing of IMP
- Height (at screening only)
- Body Mass Index (BMI)
- Body temperature
- Respiratory rate
- Adverse Event assessment/reporting
- Dynamometer test

7.3.3 Electrocardiogram (ECG)

- 12 lead electrocardiogram (performed prior to administration of study drug)

7.3.4 Lung Function Tests (performed prior to administration of study drug)

- Lung Function Tests (Forced Vital Capacity (FVC))
  Ventilatory support is indicated when the FVC is less than 50 per cent of predicted in many patients with respiratory muscle weakness[51] therefore an FVC ≥50% of predicted value for gender, height and age is required for trial entry. FVC will also be measured at Week 12, Week 24 and 2 weeks after the end of treatment. Should patients continue treatment beyond 24 weeks, FVC will be measured as per the Schedule of Assesments
- Sniff Nasal Inspiratory Pressure (SNIP). Results must be recorded as the mean of three recordings and the same nostril used at baseline must be used for subsequent visits/assessments

7.3.5 Quality of Life (performed prior to administration of study drug)

- Amyotrophic Lateral Sclerosis Functional Rating Scale (Revised) (ALSFRS-R)
- QOL ALSAQ-40

Quality of Life assessments will be performed at treatment weeks (Weeks 1 – 10) and during follow up visits (Week 12, 16, 20 and 24). Should patients continue treatment beyond 10 weeks Quality of Life assessments will be performed as per the Schedule of Assessments.

All patients will be invited to provide data on Quality of Life in a single point long-term remote follow up visit conducted over an 8 week period starting in Quarter 1 of 2021.

7.3.6 Pregnancy testing

Urine pregnancy test for WOCBP ≤7 days prior the first dose of IMP and every 4 weeks during the study. Should patients continue treatment beyond 10 weeks urine pregnancy test for WOCBP will also be performed regularly as per the Schedule of Assessments.

7.4 Sample Collection

7.4.1 Blood samples

7.4.1.1 Pharmacokinetic samples

For all patients, blood samples for the determination of ILB levels will be collected as detailed in the Schedule of Assessments in the study protocol.

Samples (plasma) for PKs should be collected from whole blood, collected in anti-coagulant-treated Vacutainer tubes (3.2% sodium citrate (blue-topped)). Serum should not be prepared.

Samples for ILB measurements should be sent (single/few batches) to:

Eurofins BioPharma Product Testing Munich GmbH
Chun-Wei Lee
Behringstr. 6-8
821 52 Planegg
Germany
+49 89 899 65 00 (phone)
Chun-WeiLee@eurofins.com

Time Point	Blood Sample Type	Volume of blood (ml)
Pre-dose (30 minutes prior to ILB injection)	Blood Plasma	14
30 minutes post-injection	Blood Plasma	3.5
60 minutes post-injection	Blood Plasma	3.5
120 minutes post –injection	Blood Plasma	3.5
150 minutes post-injection	Blood Plasma	3.5
180 minutes post-injection	Blood Plasma	3.5
240 minutes post-injection	Blood Plasma	3.5
360 minutes post-injection	Blood Plasma	3.5

Please use the current version of the laboratory manual for further information.

The remaining PK samples should be stored in a -80ºC freezer and will be shipped in batches for HGF measurement (exploratory biomarker) to the Neuregenix laboratory.

SAMPLE

7.4.1.2 NfL levels (performed prior to administration of study drug)

- 10mls Whole Blood collected in EDTA-treated Vacutainer tubes (lavender/purple top) (to allow for 4-5mls plasma for analysis).

Samples should be stored in a -80ºC freezer and will be shipped in batches to Sahlgrenska University Hospital, (Neurokemi lab), Gothenburg, Sweden

Please see the latest version of the laboratory manual for further information

7.4.1.3 Exploratory Biomarker Blood Samples (performed prior to administration of study drug)

Exploratory biomarker samples for:

a) HPLC analysis of purines, pyrimidines, oxidative/nitrosative stress compounds (n = 60)
b) HPLC analysis of fat-soluble vitamins and antioxidants (n = 60)
c) HPLC analysis of amino acids and amino group-containing compounds (n = 60)
d) Spectrophotometric analysis of lactate (n = 60)
e) PBMC samples will be collected for analysis of change in phenotypic balance
f) HGF release in plasma following ILB administration (ELISA from PK samples)
g) Creatine kinase and Myoglobin from plasma as markers of muscle atrophy associated with ALS

Please see the latest version of the laboratory manual for further information.

Samples	Visit	Blood sample type	Volume (ml)
NfL	Wk 1, Wk 5, Wk 11/12*, week 24, week 26**	Plasma	6
Exploratory Biomarker	Wk1, Wk5, Wk11/12*, Wk24, Wk26**	Serum	5
Exploratory Biomarker	Wk1, Wk5, Wk11/12*, Wk24, Wk 26**	PBMC	10

*Week 11 if patients continue onto the treatment extension phase of the trial. Week 12 if patients are on end of treatment visit.
** Patients who complete 24 weeks of treatment and do not continue on to further treatment will have an end of treatment visit week 26.
Please see Appendix 12 and the latest version of the laboratory manual for further information.

7.4.1.4 Research Samples

Any remaining blood samples at the end of the study will be shipped and stored at the Sponsor's biobank for use in future ethically and scientifically approved research in the UK or overseas; including genetic studies, research which may use animals or in vitro models, and research involving private or commercial companies.

SAMPLE

Please see the latest version of the laboratory manual for further information.

7.4.2 Urine Samples (performed prior to administration of study drug)

Where possible the first urine of the morning should be obtained for each patient for:

- Urinary $p75^{ECD}$ levels

Please see Appendix 12 and the latest version of the laboratory manual for further information

7.5 Dose Modifications

During the dosing period, if patients experience any adverse events the following criteria must be followed:

Table 2 Dose Modifications for Adverse Events

CTCAE Grade	ACTION
Grade 1-2	At the discretion of the treating clinician continue treatment
Grade 3	The treating clinician should consider stopping study treatment at this point. Or omit dose until resolved to grade 1-2
Grade 4	Discontinue study treatment

Definition of Grade – see NCI Common Terminology Criteria for Adverse Events version 4 (NCI CTCAE v4)

7.6 Treatment Interruptions

At the discretion of the treating clinician, patients can recommence study drug following omitted dose(s) up until Week 10 and after consent to continue treatment after week 10.The treatment interruption guidelines will also apply should treatment fall over a public holiday (e.g. Easter break) and due to logistics a patient is unable to attend a hospital visit and receive study drug.

7.7 Treatment Compliance

ILB will be administered subcutaneously to patients at the site; they will receive study treatment directly from the investigator or designee (as documented on the study delegation log), under medical supervision. The date and time of each dose administered in the clinic will be recorded in the source documents.

The full and accurate record of drug preparation, (batch numbers, and time of preparation, dispensing and time of actual administration) will be maintained by the individual site pharmacy. This information will be provided to the sponsor upon request and some information will be recorded in the case report

SAMPLE

forms. Full drug accountability records will be maintained by the participating site and some of this information will also be recorded within the case report forms.

Please refer to the current ALS pharmacy manual.

7.8 Treatment Discontinuation

See Patient Stopping Rules and Trial Stopping Rules (see protocol page (xiv))

7.9 Treatment after the end of the study

See protocol page (xiv) Rescue Medication

7.10 Supportive Treatment

See RESCUE MEDICATION page (xiv) of the protocol

7.11 Concomitant Medication

Patients may continue with previous concomitant treatments (i.e. prescription, non-prescription or alternative therapies) at the same doses and schedule as prior to the start of trial treatment, at the discretion of the local investigator provided the medication is not prohibited within the exclusion criteria for trial participation.

All medication that each participant is taking at the time, and within 3 months prior, to enrolment will be recorded. New medications or changes to current medications during the trial will also be recorded.

The pharmaceutical/trade name, dose, route of administration, indication, start/stop date of each new medication within the trial will be recorded. Any drug that is licensed within the United Kingdom and Europe, that is deemed necessary for the participant's health-care, will be permitted at the discretion of the Chief Investigator.

7.12 Prohibited Medication

The following medications or therapies are prohibited:

- Any other investigational medicinal or biological product, within 3 months of screening
- Use of Riluzole within 28 days of 1st dose of ILB
- Use of Riluzole during study treatment. Patients considered suitable to commence Riluzole following discontinuation of study treatment must have a ≥ 7 day wash-out period between the last dose of ILB and the first dose of Riluzole
- Any use of antioxidants, edaravone, tirasemtiv or CK-2127107 within 1 month before the screening visit
- Any botulinum toxin use within 3 months before the screening visit
- Current use of an anticoagulant e.g Warfarin, Aspirin, Clopidogrel, any novel anticoagulants (NOAC)s or low molecular weight subcutaneous heparin

7.13 Contraception and Pregnancy

All WOCBP must agree to use a highly effective method of contraception as defined in the HMA_CRFG guidelines (see Appendix 8) from the Screening visit and throughout the trial period and following the

SAMPLE

last dose of study drug. Lactating women must agree to discontinue breast feeding before trial investigational medicinal product administration.

For the purposes of this trial, a female patient of childbearing potential is a woman who has not had a hysterectomy, bilateral oophorectomy, or medically-documented ovarian failure. Women ≤ 50 years of age with amenorrhea of any duration will be considered to be of childbearing potential.

Men, if not vasectomised, must agree to use barrier contraception (condom plus spermicide) during heterosexual intercourse from screening through to trial completion and from the last dose of trial investigational medicinal product.

Refer to section 8.1.2.1 if a pregnancy is reported, either that of a female participant, or the partner of a male participant.

7.14 Patient Follow Up

This follow up schedule is based on patients who discontinue treatment after 10 weeks of drug administration..

End of treatment visit (Week 12) (Day 78 +/-3 days)

Visit 12

(2 weeks after last dose of IMP)

- ALSFRS-R
- Concomitant drug history
- Vital signs (blood pressure, heart rate, body temperature, pulse oximetry and respiration rate). Results of oximetry should be the average of three recordings. Body weight and Body Mass Index (BMI)
- ECG
- Lung Function Tests (FVC)
- SNIP
- Haematology tests (FBC, ESR)
- Coagulation tests (to include INR, APTT and PT)
- Biochemistry tests (Troponin I, C-reactive protein (CRP), Glucose, Calcium
- Creatinine Kinase
- Urea & Electrolytes (U&Es), Liver Function Tests (LFTs)
- Thyroid Function Tests (TSH, Free T4)
- Immunoglobulins (IgG, IgM)
- Quality of Life questionnaire (ALSAQ-40)
- Urinary p75^{ECD}
- NfL levels
- Collection of exploratory biomarker blood samples
- Pregnancy test for WOCBP
- Adverse Event assessment/reporting

An end of treatment visit must be performed 2 weeks from the last dose of ILB regardless of how many treatment doses a patient has received.

SAMPLE

In the event a patient discontinues treatment prior to the end of week 10 the follow up schedule as detailed in the Schedule of Assessments 1 should be followed using the Week 16 schedule as a guide for visits. If drug administration ends at week 10, follow up visits should continue every 4 weeks from week 12 at which point patients should continue with the follow up schedule as per the Schedule of Assessments 1 table. i.e. week 12, week 16, week 20 and week 24 follow up visits.

If drug administration is approved to continue after week 10 but the patient discontinues early, prior to the end of week 24, follow up visits should continue every 4 weeks until week 24 where an end of treatment visit will occur.

Follow up schedule for patients who finish treatment at week 10

Week 16

Visit 13

- ALSFRS-R
- Concomitant drug history
- Vital signs (blood pressure, heart rate, body temperature, pulse oximetry and respiration rate). Results of oximetry should be the average of three recordings. Body weight
- Haematology tests (FBC, ESR)
- Coagulation tests (to include INR, APTT and PT)
- Biochemistry tests (Troponin I, C-reactive protein (CRP), Glucose, Calcium)
- Urea & Electrolytes (U&Es), Liver Function Tests (LFTs)
- Quality of Life Questionnaire (ALSAQ-40)
- Adverse Event assessment/reporting

SAMPLE

Week 20

Visit 14

- ALSFRS-R
- Concomitant drug history
- Vital signs (blood pressure, heart rate, body temperature, pulse oximetry and respiration rate). Results of oximetry should be the average of three recordings. Body weight
- Haematology tests (FBC, ESR)
- Coagulation tests (to include INR, APTT and PT)
- Biochemistry tests (Troponin I, C-reactive protein (CRP), Glucose, Calcium)
- Urea & Electrolytes (U&Es), Liver Function Tests (LFTs)
- Quality of Life Questionnaire (ALSAQ-40)
- Adverse Event assessment/reporting

Week 24

Visit 15

- ALSFRS-R
- Concomitant drug history
- Vital signs (blood pressure, heart rate, body temperature, pulse oximetry and respiration rate). Results of oximetry should be the average of three recordings. Body weight and Body Mass Index (BMI)
- ECG
- Lung Function Tests (FVC)
- SNIP
- Haematology tests (FBC, ESR)
- Coagulation tests (to include INR, APTT and PT)
- Biochemistry tests (Troponin I, C-reactive protein (CRP), Glucose, Calcium
- Creatinine Kinase
- Urea & Electrolytes (U&Es), Liver Function Tests (LFTs)
- Thyroid Function Tests (TSH, Free T4)
- Immunoglobulins (IgG, IgM)
- Quality of Life questionnaire (ALSAQ-40)
- Urinary p75^{ECD}
- NfL levels
- Collection of exploratory biomarker blood samples
- Pregnancy test for WOCBP
- Adverse Event assessment/reporting

SAMPLE

Follow-up schedule 2 weeks after final IMP Administration

- ALSFRS-R
- Concomitant drug history
- Vital signs (blood pressure, heart rate, body temperature, pulse oximetry and respiration rate). Results of oximetry should be the average of three recordings. Body weight and Body Mass Index (BMI)
- ECG
- Lung Function Tests (FVC)
- SNIP
- Haematology tests (FBC, ESR)
- Coagulation tests (to include INR, APTT and PT)
- Biochemistry tests (Troponin I, C-reactive protein (CRP), Glucose, Calcium
- Creatinine Kinase
- Urea & Electrolytes (U&Es), Liver Function Tests (LFTs)
- Thyroid Function Tests (TSH, Free T4)
- Immunoglobulins (IgG, IgM)
- Quality of Life questionnaire (ALSAQ-40)
- Urinary $p75^{ECD}$
- NfL levels
- Collection of exploratory biomarker blood samples
- Pregnancy test for WOCBP
- Adverse Event assessment/reporting

For patients who continue treatment, an end of treatment visit will occur 2 weeks after the last ILB injection, following the sample assessment as detailed for week 24 above.

Single Point Long-Term Remote Follow up Visit

All patients will be invited to provide quality of life data during an 8 week single point remote follow-up visit period starting in Quarter 1 of 2021.

Patients will be asked to re-consent electronically. After electronic consent has been given, the visit will be completed by the ALS study clinician via video conference and the following assessed:

- ALSFRS-R
- ALSAQ-40
- Concomitant medication including any current ALS treatment

SAMPLE

7.15 Patient Withdrawal

The Investigator will make every reasonable effort to keep each patient on treatment. However, if the Investigator removes a patient from the trial treatment or if (s)he declines further participation final assessments will be performed, if possible. All the results of the evaluations and observations, together with a description of the reasons for trial withdrawal, must be recorded in the CRF.

In the event of a patient's decision to withdraw from the trial, the Investigator must ascertain from which aspects of the trial the patient wishes to withdraw, and record the details on the appropriate CRF. All patients will continue to be followed-up (if they agree), and all information and tissue samples collected up until the point of retraction, will be retained and analysed.

Patients who are removed from treatment due to adverse events (clinical or laboratory) will be treated and followed up according to accepted medical practice. All pertinent information concerning the outcome of such treatment must be recorded in the CRF.

The following are justifiable reasons for the Investigator to withdraw a patient from trial:

- Withdrawal of consent
- Serious violation of the trial protocol (including persistent patient attendance failure and persistent non-compliance)
- Withdrawal by the Investigator for clinical reasons not related to the trial drug treatment

This section should also be applied in the event of continuation of treatment beyond 10 weeks.

A patient that has not received any IMP treatment will be non-evaluable and therefore will be replaced.

SAMPLE

8. ADVERSE EVENT REPORTING

The collection and reporting of Adverse Events (AEs) will be in accordance with the Medicines for Human Use Clinical Trials Regulations 2004 and its subsequent amendments. Definitions of different types of AE are listed in Appendix 3. The Investigator should assess the seriousness and causality (relatedness) of all AEs experienced by the patient (this should be documented in the source data) with reference to the *current IB Investigator Brochure.*

8.1 Reporting Requirements

8.1.1 Adverse Events

All medical occurrences which meet the definition of an AE (see Appendix 4 for definition) should be reported. Please note this includes abnormal laboratory findings including those that are not clinically significant.

8.1.1.1 Pre-existing conditions

Any pre-existing conditions should be reported as medical history, and should not be reported as an AE unless the condition worsens by at least one CTCAE grade during the trial. The condition, however, must be reported in the CRF.

8.1.2 Serious Adverse Events

Investigators should report AEs that meet the definition of an SAE (see Appendix 3 for definition)

8.1.2.1 Monitoring pregnancies for potential Serious Adverse Events

It is important to monitor the outcome of pregnancies of patients in order to provide SAE data on congenital anomalies or birth defects.

In the event that a patient or their partner becomes pregnant during the SAE reporting period please complete a Pregnancy Notification Form (providing the patient's details) and return to the ALS Trials Office as soon as possible. If it is the patient who is pregnant provide outcome data on a follow-up Pregnancy Notification Form. Where the patient's partner is pregnant consent must first be obtained and the patient should be given a Pregnancy Release of Information Form to give to their partner. If the partner is happy to provide information on the outcome of their pregnancy they should sign the Pregnancy Release of Information Form. Once consent has been obtained provide details of the outcome of the pregnancy on a follow-up Pregnancy Notification Form. If appropriate also complete an SAE Form as detailed below.

SAMPLE

8.1.2.2 Liver Related Toxicity: Biochemistry

All liver related toxicity: (Biochemistry blood tests) will be reported in accordance with Appendix 5 of the study protocol. Specific information will be provided on a Serious Adverse Event Case Report Form and liver toxicity Case Report Form as applicable. Reporting of these events will need to be via expedited Serious Adverse Event procedures detailed in Section 8.2.1.2 of the protocol. The information relating to these procedures which will be reported under medically significant events (SAE categorisation) is also summarised below.

Other Medically significant events that need to be reported as an SAE include:

- Is associated with liver injury and impaired liver function defined as:

o ALT > 3xULN and total bilirubin# > 2xULN (>35% direct), or ALT > 3xULN and INR >1.5.

Serum bilirubin fractionation should be performed if testing is available; if unavailable, measure urinary bilirubin via dipstick. If fractionation is unavailable and ALT > 3xULN and total bilirubin > 2xULN, then the event is still to be reported as an SAE. If INR measurement is obtained, the value is to be recorded on the SAE form.

8.1.3 Reporting period

Details of all AEs will be documented and reported from the signing of the informed consent and will include the collection of all baseline AEs. AE collection will continue until 30 days after the end of treatment visit.

Details of SAEs will be documented and reported for each patient from the date of consent until 30 days after their last administration of trial treatment.

SAMPLE

8.2 Reporting Procedure

8.2.1 Site

8.2.1.1 Adverse Events

AEs should be reported on an AE Form (and where applicable on an SAE Form). AEs should be completed at each visit and the information recorded on the case report form (eRDC) / or paper form as appropriate.

AEs will be reviewed using the Common Terminology Criteria for Adverse Events (CTCAE), version 4.0 (see Appendix 4). Any AEs experienced by the patient but not included in the CTCAE should be graded by an Investigator and recorded on the AE Form in accordance with the following guidelines: Grade (1) Mild, Grade (2) Moderate, Grade (3) Severe, Grade (4) Life-threatening or Grade (5) Death. For each sign/symptom, the highest grade observed since the last visit should be recorded.

8.2.1.2 Serious Adverse Events

For more detailed instructions on SAE reporting refer to the SAE Form Completion Guidelines contained in the Investigator Site File (ISF).

AEs defined as serious and which require reporting as an SAE should be reported on an SAE Form. When completing the form, the Investigator will be asked to define the causality and the severity of the AE which should be documented using the CTCAE version 4.0.

On becoming aware that a patient has experienced an SAE, the Investigator (or delegate) must complete, date and sign an SAE Form. The form should be scanned to the Trials Office email address listed below as soon as possible and no later than 24 hours after first becoming aware of the event:

To report an SAE by email, email a scanned copy of the SAE form to:

reg@trials.bham.ac.uk (cc in trial mailbox: als@trials.bham.ac.uk)

On receipt the ALS Trials Office will allocate each SAE a unique reference number. This number will be included in a response e-mail as acknowledgement of receipt. If confirmation of receipt is not received within 1 working day please contact the Trials Office. The SAE reference number should be quoted on all correspondence and follow-up reports regarding the SAE. The response e-mail from the ALS Trials Office should be filed with the SAE Form in the ISF.

For SAE Forms completed by someone other than the Investigator the Investigator will be required to countersign the original SAE Form to confirm agreement with the causality and severity assessments. The form should then be returned to the ALS Trials Office in the post and a copy kept in the ISF.

Investigators should also report SAEs to their own Trust in accordance with local practice.

8.2.1.3 Provision of follow-up information

Patients should be followed up until resolution or stabilisation of the event. Follow-up information should be provided on a new SAE Form (refer to the SAE Form Completion Guidelines for further information).

SAMPLE

8.2.2 Trials Office

On receipt of an SAE Form seriousness and causality will be determined independently by a Clinical Coordinator. An SAE judged by the Investigator or Clinical Coordinator to have a reasonable causal relationship with the trial medication will be regarded as a Serious Adverse Reaction (SAR). The Clinical Coordinator will also assess all SARs for expectedness. If the event meets the definition of a SAR that is unexpected (i.e. is not defined in the Investigator Brochure) it will be classified as a Suspected Unexpected Serious Adverse Reaction (SUSAR).

8.2.3 Reporting to the Competent Authority and Research Ethics Committee

8.2.3.1 Suspected Unexpected Serious Adverse Reactions

The ALS Trials Office will report a minimal data set of all individual events categorised as a fatal or life threatening SUSAR to the Medicines and Healthcare products Regulatory Agency (MHRA), Research Ethics Committee (REC) and to the Sponsor (University of Birmingham) within 7 days. Detailed follow-up information will be provided within an additional 8 days.

All other events categorised as SUSARs will be reported within 15 days.

8.2.3.2 Serious Adverse Reactions

The ALS Trials Office will report details of all SARs (including SUSARs) to the MHRA and REC annually from the date of the Clinical Trial Authorisation, in the form of a Developmental Safety Update Report (DSUR).

8.2.3.3 Adverse Events

Details of all AEs will be reported to the MHRA on request.

8.2.3.4 Other safety issues identified during the course of the trial

The MHRA and REC will be notified immediately if a significant safety issue is identified during the course of the trial.

8.2.4 Investigators

Details of all SUSARs and any other safety issue which arises during the course of the trial will be reported to Principal Investigators. A copy of any such correspondence should be filed in the ISF.

8.2.5 Data Monitoring Committee

The independent Data Monitoring Committee (DMC) will review all SAEs.

8.2.6 Manufacturer of Investigational Medicinal Product

All Serious Adverse Reactions (SARs) and Suspected Unexpected Serious Adverse Reaction (SUSARs) will be reported to the TikoMed Medical Expert by email or fax within 72 hours of the sponsor categorising the event as a SAR/SUSAR. Subsequent acknowledgement from TikoMed is expected within 48 hours of receipt.

SAMPLE

9. DATA HANDLING AND RECORD KEEPING

9.1 Data Collection

Data will be collected during this clinical trial by utilising both electronic remote data capture (eRDC) systems and paper case report forms (CRF).

The Case Report Form (CRF) will comprise the following forms:

Table 3 ALS Trial Case Report Form

Form	Summary of data recorded	Schedule for submission	Paper/eRDC
Eligibility Form (checklist)	Confirmation of eligibility and satisfactory screening investigations where necessary	Faxed/emailed at point of registration	Paper
Registration Form	Patient details and registration number	Faxed/emailed at point of registration	Paper
Treatment Extension Registration Form	Patient details and registration number	Faxed/emailed at point of re-consent	Paper
Screening Visit	Visit details including confirmation of blood tests and vital signs, records of medical history (including abnormal blood tests/values) and concomitant medications		eRDC
Treatment Form	Actual IMP dose, dates given, details and reasons for dose reductions and delays; details of supportive treatment, confirmation of blood tests, vital signs, records of AEs and concomitant medications	Within 1 week of treatment visit	eRDC
Treatment Form (Week 11 onwards – as applicable)	Actual IMP dose, dates given, details and reasons for dose reductions and delays; details of supportive treatment, confirmation of blood tests, vital signs, records of AEs and concomitant medications	Within 1 week of treatment visit	eRDC

SAMPLE

Adverse Event Form	AE start and stop dates, grade, outcome and causality assessed by clinician	Alongside each visit form	eRDC
Concomitant Medication Form	Concomitant medications start and stop dates, dose, route, frequency and indication	Alongside each visit form	eRDC
ALSFRS-R	Patient response to ALSFRS	Within 2 weeks of visit date	Paper initially and then transferred to eRDC by study team.
ALSAQ-40 Questionnaire	Patient response to ALSAQ-40	Within 2 weeks of visit date	Paper initially and then transferred to eRDC by study team.
Biochemistry	Date, visit, results, clinical significance	Within 2 weeks of visit date	eRDC
Haematology	Date, visit, results, clinical significance	Within 2 weeks of visit date	eRDC
Pharmacokinetics	Date, visit, sample collection, time taken	Within 2 weeks	eRDC
Biomarker Sample	Date, visit, sample collection, time taken	Within 2 weeks	eRDC
Liver Specific Toxicity – additional information form	For the collection of additional information see Section 8.1.2.2 and Appendix 5 of the protocol	Within 72 hours of liver toxicity event i.e elevated ALT, Bilirubin (see Appendix 5)	eRDC
Follow-up Form	Date, visit details including clinical assessment and results of trial procedures	Within 2 weeks of final visit	eRDC
Single point Long-term Remote Follow Up Form	Date, Re-consent, ALSFRS-R, ALSAQ-40, Concomitant Medication	Quarter 1 2021	eRDC
Unscheduled visit form	As required		eRDC
End of treatment form	Date and visit details including a Summary of protocol defined treatment and reasons for treatment discontinuation, clinical assessment and results of trial procedures	Within 2 weeks of visit date	eRDC

SAMPLE

Death Form	Date and cause of death	Immediately upon notification of patient's death	eRDC
Deviation Form	Completed in the event of a deviation from the protocol	Immediately upon discovering deviation	eRDC
Withdrawal Form	Used to notify the Trials Office of patient withdrawal from the trial	Immediately upon patient withdrawal	eRDC

Ad hoc forms

Serious Adverse Event Form

Relapse/Death Report Form

Pregnancy Notification Form

9.2 Data Collection Procedure

9.2.1 Electronic Remote Data Capture (eRDC) systems only

The majority of patient clinical data will be collected via bespoke electronic data capture systems (ERDC) developed by the CRCTU programming team. Access to the ERDC system will be via standard web portals software (Microsoft Internet Explorer Version 8+, Firefox and/or Chrome) and individual site staff will be granted access to the system via unique username and password. Additional detailed information/training will be provided by the sponsor via 1:1 training throughout the study and an ERDC guidance/completion document will be provided to study site personnel.

9.2.2 Paper Case Report Forms only

The CRF must be completed, signed/dated and returned to the ALS Trials Office by the Investigator or an authorised member of the site research team (as delegated on the Site Signature and Delegation Log) within the timeframe listed above. *The exceptions to this are the SAE Form and Withdrawal Form which must be co-signed by the Investigator.*

Entries on the CRF should be made in ballpoint pen, in blue or black ink, and must be legible. Any errors should be crossed out with a single stroke, the correction inserted and the change initialled and dated. If it is not obvious why a change has been made, an explanation should be written next to the change.

Data reported on each form should be consistent with the source data or the discrepancies should be explained. If information is not known, this must be clearly indicated on the form. All missing and ambiguous data will be queried. All sections are to be completed before returning.

Data not captured in the source data for e.g. Quality of Life, this data will be recorded directly onto the CRF.

In all cases it remains the responsibility of the Investigator to ensure that the CRF has been completed correctly and that the data are accurate. The completed originals should be sent to the ALS Trials Office and a copy filed in the Investigator Site File.

SAMPLE

Trial forms may be amended by the Trials Office, as appropriate, throughout the duration of the trial. Whilst this will not constitute a protocol amendment, new versions of the form must be implemented by participating sites immediately on receipt.

9.3 Archiving

It is the responsibility of the Principal Investigator to ensure all essential trial documentation and source records (e.g. signed Informed Consent Forms, Investigator Site Files, Pharmacy Files, patients' hospital notes, copies of CRFs etc) at their site are securely retained for at least 25 years after the end of the trial or following the processing of all biological material collected for research, whichever is the later. Do not destroy any documents without prior approval from the CRCTU Document Storage Manager.

SAMPLE

10. QUALITY MANAGEMENT

10.1 Site Set-up and Initiation

All sites will be required to sign a *Clinical Study Site Agreement* prior to participation. In addition all participating Investigators will be asked to sign the necessary agreements and *Investigator registration forms* and supply a current CV to the ALS Trials Office. All members of the site research team will also be required to sign the *Site Signature and Delegation Log,* which should be returned to the ALS Trials Office. Prior to commencing recruitment all sites will undergo a process of initiation. Key members of the site research team will be required to attend either a meeting or a teleconference covering aspects of the trial design, protocol procedures, Adverse Event reporting, collection and reporting of data and record keeping. Sites will be provided with an Investigator Site File *and a Pharmacy File* containing essential documentation, instructions, and other documentation required for the conduct of the trial. The ALS Trials Office must be informed immediately of any change in the site research team.

10.2 On-site Monitoring

Monitoring will be carried out as required following a risk assessment and as documented in the *ALS* Quality Management Plan. Additional on-site monitoring visits may be triggered for example by poor CRF return, poor data quality, low SAE reporting rates, excessive number of patient withdrawals or deviations. If a monitoring visit is required the Trials Office will contact the site to arrange a date for the proposed visit and will provide the site with written confirmation. Investigators will allow the *ALS* trial staff access to source documents as requested.

10.3 Central Monitoring

Where a patient has given explicit consent sites are requested to send in copies of signed Informed Consent Forms for in-house review.

Trials staff will be in regular contact with the site research team to check on progress and address any queries that they may have. Trials staff will check incoming Case Report Forms for compliance with the protocol, data consistency, missing data and timing. Sites will be sent Data Clarification Forms requesting missing data or clarification of inconsistencies or discrepancies.

Sites may be suspended from further recruitment in the event of serious and persistent non-compliance with the protocol and/or GCP, and/or poor recruitment. Any major problems identified during monitoring may be reported to *the Trial Management Group,* and the relevant regulatory bodies. This includes reporting serious breaches of GCP and/or the trial protocol to the Research Ethics Committee (REC) and the Medicines for Healthcare products Regulatory Agency (MHRA).

10.4 Audit and Inspection

The Investigator will permit trial-related monitoring, audits, ethical review, and regulatory inspection(s) at their site, providing direct access to source data/documents.

Sites are also requested to notify the ALS Trials Office of any MHRA inspections.

SAMPLE

10.5 Notification of Serious Breaches

In accordance with Regulation 29A of the Medicines for Human Use (Clinical Trials) Regulations 2004 and its amendments the Sponsor of the trial is responsible for notifying the licensing authority in writing of any serious breach of:

- The conditions and principles of GCP in connection with that trial or;
- The protocol relating to that trial, within 7 days of becoming aware of that breach

For the purposes of this regulation, a "serious breach" is a breach which is likely to effect to a significant degree:

- The safety or physical or mental integrity of the subjects of the trial; or
- The scientific value of the trial

Sites are therefore requested to notify the Trials Office of a suspected trial-related serious breach of GCP and/or the trial protocol. Where the Trials Office is investigating whether or not a serious breach has occurred sites are also requested to cooperate with the Trials Office in providing sufficient information to report the breach to the MHRA where required and in undertaking any corrective and/or preventive action.

SAMPLE

11. END OF TRIAL DEFINITION

The end of trial will be the date of Last Patient Last Visit (LPLV). The Trials Office will notify the MHRA and the REC that the trial has ended and a summary of the clinical trial report will be provided within 12 months of the end of trial.

After closure of the trial with the MHRA the Sponsor is no longer required to notify the MHRA and main REC of changes of Principal Investigator. However, sites should continue to notify the Trials Office of changes in Principal Investigator by completing and returning (where required) an Investigator Registration Form together with a current signed and dated CV.

As a result of the ongoing COVID-19 pandemic, and the high at risk status of study participants, further recruitment and study treatment was paused temporarily. A decision was made by the funders and sponsor to terminate the study prematurely in October 2020. Last patient treatment visits were conducted in March 2020, however further follow up visits are planned to start in Quarter 1 of2021. The last remote follow-up visit will be regarded as the last data capture for this trial.

12. STATISTICAL CONSIDERATIONS

12.1 Definition of Outcome Measures

Outcome measures are listed in Section 2.

12.2 Analysis of Outcome Measures

Numerical outcomes will be summarised and presented as means and standard deviations where the outcome is approximately normal; or median and inter-quartile ranges where non-normal.

12.2.1 Analysis of Primary Outcome Measures

12.2.1.1 Safety

The number of SAEs and AEs will be calculated and summarised. Furthermore, the number of patients experiencing each will be calculated. Each of these analyses will be broken down by grade, relatedness, event type (admitting event for SAEs); expectedness (SAEs only) and sequelae (SAEs only).

12.2.1.2 Tolerability

Line listings for all intolerable adverse events will be given. The number of intolerable events will be calculated for each patient and summarised.

12.2.1.3 Quantity of study drug administered

Total drug administered, number of administrations, number and length of interruptions, and number of discontinuations will be calculated for each patient and summarised.

12.2.2 Analysis of Secondary Outcome Measures

Repeated measures numerical outcomes will be analysed by hierarchical models, with patient-level effects to account from baseline value and progression with respect to time. Transformations of the time variable and smoothed terms (e.g. splines) will be considered if the outcomes are found to be non-linear in time

SAMPLE

ALSFRS-R and ALSAQ-40 are very granular ordinal variables. They have 49 and 101 levels respectively so a categorical analysis is not feasible. There are many instances in the literature of these outcomes being analysed as numbers[52-56]. As such, we will analyse these outcomes as numbers.

12.3 Planned Sub Group Analyses

There are no sub-group analyses planned.

12.4 Planned Interim Analysis

Interim analyses of safety outcomes and available efficacy outcomes will be presented to the relevant independent oversight committee after the sentinel patients and periodically thereafter.

Proposed analysis for publications will occur at the following end points:

-
- We propose to conduct and submit for publication an analysis of available primary and secondary outcomes for all fifteen patients after the fifteenth patient has been evaluated to the fullest extent over the initial 10 week dosing period.
- Further analysis of outcomes will be submitted for publication after all patients who continue treatment complete 24 weeks of treatment.

12.5 Planned Final Analyses

We will seek to conduct and distribute for publication the final analysis within 6 months of the final protocol assessment of the final patient.

12.6 Power Calculations

No sample size calculations have been undertaken. The sample size has been selected based on what is feasible to be recruited at a single centre in a reasonable timeframe for this phase of clinical trial.

SAMPLE

13. TRIAL ORGANISATIONAL STRUCTURE

13.1 Sponsor

The trial is sponsored by the University of Birmingham

13.2 Coordinating Centre

The trial is being conducted under the auspices of the Cancer Research UK Clinical Trials Unit (CRCTU), University of Birmingham according to their local procedures.

13.3 Trial Management Group

The Trial Management Group (TMG) will be responsible for the day-to-day running and management of the trial and will meet by teleconference or in person as required. Members of the TMG include the Chief Investigator (CI), CRCTU Trial Management Team Leader, Senior Trial Coordinator, Trial Coordinator, Lead Trial Statistician and a Pharmacy representative. The TMG will meet or hold a teleconference every 2nd month during recruitment, or as required.

13.4 Trial Steering Committee

This clinical trial will not require a separate stand-alone Trial Steering Committee. Both the Data Monitoring Committee (DMC) and the TMG will undertake traditional roles of the TSC.

13.5 Data Monitoring Committee

Data analyses will be supplied in confidence to an independent Data Monitoring Committee (DMC), which will be asked to give advice on whether the accumulated data from the trial, together with the results from other relevant research, justifies the continuing recruitment of further patients. The DMC will operate in accordance with a trial specific charter based upon the template created by the Damocles Group. *The DMC will meet as soon as possible following the recruitment of the first sentinel patient to discuss the data and safety following the recruitment of the first patient. The DMC will meet again as soon as possible following the recruitment of the second sentinel patient. During the general recruitment phase of the trial the DMC is scheduled to meet three monthly via teleconference and annually thereafter.*

Additional meetings may be called if recruitment is much faster than anticipated and the DMC may, at their discretion, request to meet more frequently or continue to meet following completion of recruitment. An emergency meeting may also be convened if a safety issue is identified. The DMC will report directly to the Trial Management Group *TMG* who will convey the findings of the DMC to the funders, *MHRA and sponsor as applicable.* The DMC may consider recommending the discontinuation of the trial if the recruitment rate or data quality are unacceptable or if any issues are identified which may compromise patient safety. The trial would also stop early if any of the Trial Stopping Rules as detailed on page (ix) of the protocol are met.

In the event that the trial is suspended the MHRA and the REC will be advised and recruitment will not recommence until after submission and approval of a Substantial Amendment.

SAMPLE

13.6 Finance

This is a clinician-initiated and clinician-led trial funded by an educational grant from industry (TikoMed). TikoMed will be supplying the investigational medicinal product (IMP), ILB free of charge to all individual NHS Trusts that will be directly treating patients as part of this clinical trial.

13.6.1 Payments to individual NHS Trusts (per-patient payment)

Refer to the Clinical Study Site Agreement for details.

SAMPLE

14. ETHICAL CONSIDERATIONS

The trial will be performed in accordance with the recommendations guiding physicians in biomedical research involving human subjects, adopted by the 18th World Medical Association General Assembly, Helsinki, Finland, June 1964, amended at the 48th World Medical Association General Assembly, Somerset West, Republic of South Africa, October 1996.

The trial will be conducted in accordance with the Research Governance Framework for Health and Social Care, the applicable UK Statutory Instruments, (which include the Medicines for Human Use Clinical Trials 2004 and subsequent amendments and the Data Protection Act 1998 and Good Clinical Practice (GCP). This trial will be carried out under a Clinical Trial Authorisation in accordance with the Medicines for Human Use Clinical Trials regulations. The protocol will be submitted to and approved by the main Research Ethics Committee (REC) prior to circulation.

Before any patients are enrolled into the trial, the Principal Investigator at each site is required to obtain local R&D approval. Sites will not be permitted to enrol patients until written confirmation of R&D approval is received by the Trials Office.

It is the responsibility of the Principal Investigator to ensure that all subsequent amendments gain the necessary local approval. This does not affect the individual clinicians' responsibility to take immediate action if thought necessary to protect the health and interest of individual patients.

SAMPLE

15. CONFIDENTIALITY AND DATA PROTECTION

Personal data recorded on all documents will be regarded as strictly confidential and will be handled and stored in accordance with the General Data Protection Regulation and Data Protection Act 2018 for health and care research. The University of Birmingham, as the Sponsor for the ALS study, will be using information from patient medical records in order to undertake this trial and will act as the data controller for the study. This means that the University of Birmingham are responsible for looking after the information and using it properly. University of Birmingham and the NHS will keep identifiable information about the patients for at least 25 years after the study has finished, to allow the results of the study to be verified if needed.

All information collected by the Sponsor will be securely stored at the Trials Office at the University of Birmingham on paper and electronically and will only be accessible by authorised personnel. The only people in the University of Birmingham who will have access to information that identifies a patient will be people who manage the study or audit the data collection process.

The NHS will use the patient`s name and contact details to contact patients about the research study, and make sure that relevant information about the study is recorded for their care, and to oversee the quality of the trial. With the patient`s permission, the research team (at site) will notify the patient`s GP that they intend to participate in the study.

With the patient's consent, their *full name (on consent form only), initials and date of birth will be collected at trial entry*. The research team (at site) will send a copy of the signed consent form in the post to the Trials Office. *This will be used to perform in-house monitoring of the consent process.*

In routine communication between the hospital and the Trials Office, the patient will only be identified by study number, initials and date of birth. Data may be provided to the Trials Office on paper or electronically.

By taking part in the study, the patient will be agreeing to allow research staff from the Trials Office at the University of Birmingham to look at the study records, including their medical records. It may be necessary to allow authorised personnel from government regulatory agencies (e.g. Medicines and Healthcare products Regulatory Agency (MHRA), the Sponsor and/or NHS bodies to have access to patient medical and research records. This is to ensure that the study is being conducted to the highest possible standards. Anonymised data from the study may also be provided to other third parties (e.g. the manufacturer of the trial treatment) for research, safety monitoring or licensing purposes.

The Investigator must maintain documents not for submission to the Trials Office (e.g. Patient Identification Logs) in strict confidence. In the case of specific issues and/or queries from the regulatory authorities, it will be necessary to have access to the complete trial records, provided that patient confidentiality is protected.

SAMPLE

16. INSURANCE AND INDEMNITY

University of Birmingham employees are indemnified by the University insurers for negligent harm caused by the design or co-ordination of the clinical trials they undertake whilst in the University's employment.

In terms of liability at a site, NHS Trust and non-Trust hospitals have a duty to care for patients treated, whether or not the patient is taking part in a clinical trial. Compensation is therefore available via NHS indemnity in the event of clinical negligence having been proven.

The University of Birmingham cannot offer indemnity for non-negligent harm. The University of Birmingham is independent of any pharmaceutical company, and as such it is not covered by the Association of the British Pharmaceutical Industry (ABPI) guidelines for patient compensation.

17. PUBLICATION POLICY

Results of this trial will be submitted for publication in a peer reviewed journal. The manuscript will be prepared by the Trial Management Group (TMG) and authorship will be determined by mutual agreement.

Any secondary publications and presentations prepared by Investigators must be reviewed by the TMG. Manuscripts must be submitted to the TMG in a timely fashion and in advance of being submitted for publication, to allow time for review and resolution of any outstanding issues. Authors must acknowledge that the trial was performed with the support of the University of Birmingham. Intellectual property rights will be addressed in the *Clinical Study Site Agreement* between Sponsor and site.

SAMPLE

18. REFERENCE LIST

1. Bellingham MC. A review of the neural mechanisms of action and clinical efficiency of riluzole in treating amyotrophic lateral sclerosis: what have we learned in the last decade? CNS Neurosci Ther. 2011;17(1):4-31.

2. Neuregenix. Effects of ILB on cultured neurons and Schwann cell lines. ILB-1.

3. Neuregenix. Effect of ILB on gene expression. ILB-3.

4. Neuregenix. Effects of ILB on cerebral metabolic alterations induced by severe traumatic brain injury in the rat: a pilot study. ILB-6.

5. Henriques A, Pitzer C, Schneider A. Neurotrophic growth factors for the treatment of amyotrophic lateral sclerosis: where do we stand? Front Neurosci. 2010;4:32.

6. Warita H, Kato M, Suzuki N, Itoyama Y, Aoki M. Clinical translation of hepatocyte growth factor for amyotrophic lateral sclerosis. Rinsho Shinkeigaku. 2012;52(11):1214-7.

7. TikoMed_AB. An open, single centre, pharmacokinetics, administration optimization, safety and tolerability study of Low Molecular Weight Dextran Sulfate (LMW-DS) in healthy volunteers. LMW-DS-101.

8. TikoMed_AB. A two-part (part A and B) dose-finding study in healthy subjects to explore safety, tolerability and efficacy of escalating single doses of Low Molecular Weight Dextran Sulfate, LMW-DS solution, administered by short-term infusion LMW-DS-102. EudraCT no: 2011-004111-23, 2013. 28 Feb 2014.

9. TikoMed_AB. A randomised, open-label, placebo-controlled, single centre study in healthy male volunteers to explore efficacy, safety and tolerability of single doses of low molecular weight dextran sulphate (LMW-DS) in combination with recombinant human granulocyte colony stimulating factor (rhG-CSF, filgrastim) and in comparison with plerixa for treatment and placebo. LMW-DS-103.

10. Gordon PH, Mitsumoto H. Chapter 20 Symptomatic therapy and palliative aspects of clinical care. Handb Clin Neurol. 2007;82:389-424.

11. Lee JR, Annegers JF, Appel SH. Prognosis of amyotrophic lateral sclerosis and the effect of referral selection. J Neurol Sci. 1995;132(2):207-15.

12. Traynor BJ, Alexander M, Corr B, Frost E, Hardiman O. Effect of a multidisciplinary amyotrophic lateral sclerosis (ALS) clinic on ALS survival: a population based study, 1996-2000. J Neurol Neurosurg Psychiatry. 2003;74(9):1258-61.

13. Miller RG, Mitchell JD, Lyon M, Moore DH. Riluzole for amyotrophic lateral sclerosis (ALS)/motor neuron disease (MND). Cochrane Database Syst Rev. 2007(1):CD001447.

14. Bensimon G, Lacomblez L, Meininger V. A controlled trial of riluzole in amyotrophic lateral sclerosis. ALS/Riluzole Study Group. N Engl J Med. 1994;330(9):585-91.

15. Lacomblez L, Bensimon G, Leigh PN, Guillet P, Meininger V. Dose-ranging study of riluzole in amyotrophic lateral sclerosis. Amyotrophic Lateral Sclerosis/Riluzole Study Group II. Lancet. 1996;347(9013):1425-31.

16. Walley T. Neuropsychotherapeutics in the UK: what has been the impact of NICE on prescribing? CNS Drugs. 2004;18(1):1-12.

17. Gordon PH. Amyotrophic Lateral Sclerosis: An update for 2013 Clinical Features, Pathophysiology, Management and Therapeutic Trials. Aging Dis. 2013;4(5):295-310.

18. Taylor JP, Brown RH, Jr., Cleveland DW. Decoding ALS: from genes to mechanism. Nature. 2016;539(7628):197-206.

19. Grönwall A, Ingelman B, Mosimann H. A dextran sulphuric acid ester with heparin activity. Uppsala Läkarförening Förhandling. 1945;50:397-404.

20. Flexner C, Barditch-Crovo PA, Kornhauser DM, Farzadegan H, Nerhood LJ, Chaisson RE, et al. Pharmacokinetics, toxicity, and activity of intravenous dextran sulfate in human immunodeficiency virus infection. Antimicrob Agents Chemother. 1991;35(12):2544-50.

21. Hiebert LM, Wice SM, Jaques LB, Williams KE, Conly JM. Orally administered dextran sulfate is absorbed in HIV-positive individuals. J Lab Clin Med. 1999;133(2):161-70.

SAMPLE

22. Komai H, Naito Y, Okamura Y. Dextran sulfate as a leukocyte-endothelium adhesion molecule inhibitor of lung injury in pediatric open-heart surgery. Perfusion. 2005;20(2):77-82.

23. Fujishima M, Omae T, Tanaka K, Iino K, Matsuo O, Mihara H. Controlled trial of combined urokinase and dextran sulfate therapy in patients with acute cerebral infarction. Angiology. 1986;37(7):487-98.

24. TikoMed_AB. An open, randomised, single-centre, cross-over, bioavailability study evaluating the pharmacokinetics, safety and local tolerability of low molecular weight dextran sulfate (LMW-DS) solution given subcutaneously and intravenously to healthy volunteers. TM-104.

25. NIAID/NIDDK. Open Randomized Multi-Center Study to Evaluate Safety and Efficacy of Low Molecular Weight Sulfated Dextran in Islet Transplantation. CIT-01.

26. Maina F, Klein R. Hepatocyte growth factor, a versatile signal for developing neurons. Nat Neurosci. 1999;2(3):213-7.

27. Tonges L, Ostendorf T, Lamballe F, Genestine M, Dono R, Koch JC, et al. Hepatocyte growth factor protects retinal ganglion cells by increasing neuronal survival and axonal regeneration in vitro and in vivo. J Neurochem. 2011;117(5):892-903.

28. Ishigaki A, Aoki M, Nagai M, Warita H, Kato S, Kato M, et al. Intrathecal delivery of hepatocyte growth factor from amyotrophic lateral sclerosis onset suppresses disease progression in rat amyotrophic lateral sclerosis model. J Neuropathol Exp Neurol. 2007;66(11):1037-44.

29. Sun W, Funakoshi H, Nakamura T. Overexpression of HGF retards disease progression and prolongs life span in a transgenic mouse model of ALS. J Neurosci. 2002;22(15):6537-48.

30. Pan W, Yu Y, Yemane R, Cain C, Yu C, Kastin AJ. Permeation of hepatocyte growth factor across the blood-brain barrier. Exp Neurol. 2006;201(1):99-104.

31. Tsuboi Y, Kakimoto K, Akatsu H, Daikuhara Y, Yamada T. Hepatocyte growth factor in cerebrospinal fluid in neurologic disease. Acta Neurol Scand. 2002;106(2):99-103.

32. Schmidt P, Magnusson C, Lundgren T, Korsgren O, Nilsson B. Low molecular weight dextran sulfate is well tolerated in humans and increases endogenous expression of islet protective hepatocyte growth factor. Transplantation. 2008;86(11):1523-30.

33. Redoxis_AB. Tolerability study of Tikomed lead compound after s.c. administration in rat; 2013b. Report No. RX0113B.

34. TikoMed_AB. Evaluation of different doses of LMW-DS on mobilisation of white blood cells and hepatocyte growth factor. 2012c. Report No. TM-MOB-REP-013.

35. TikoMed_AB. Evaluation of low dose LMW-DS on hepatocyte growth factor and on mobilisation of white blood cells at different time points. 2012d. Report No. TM-MOBREP-014.

36. TikoMed_AB. Effect of LMW-DS administration to healthy volunteers on plasma concentration of brain-derived neurotrophic factor (BDNF). TM-REP-HEX-002, 2014. 15 October 2014.

37. Tadic V, Malci A, Goldhammer N, Stubendorff B, Sengupta S, Prell T, et al. Sigma 1 receptor activation modifies intracellular calcium exchange in the G93A(hSOD1) ALS model. Neuroscience. 2017;359:105-18.

38. Finland CRD. The Effect of Compound TM1 or TM2 on MOG1-125 Induced Chronic EAE in Rats. C144113, 2013. 23 October 2013.

39. Redoxis_AB. Efficacy validation in an animal model of multiple sclerosis i.e. MOG35-55 induced Experimental Autoimmune Encephalomyelitis (EAE) in mice. RX0083C, 2013. 18 January 2013(12 April 2013).

40. Pharmaseed_Ltd. Angiogenic efficacy of TM-700 in the rat stroke model – Transient Middle Cerebral Artery Occlusion. TKM-003-EF, 2014. 29 October 2014.

41. Pharmaseed_Ltd. Evaluation of angiogenesis in critical hind-limb ischemia mouse model TKM-001-EF, 2014. 8 March 2014.

42. Pharmaseed_Ltd. The effect of TM-700 on angiogenesis in a mouse model of critical limb ischemia. TKM-002-EF, 2014. 27 November 2014.

43. Pharmaseed_Ltd. Evaluation of TM-700 angiogenesis efficacy in a critical hind-limb ischemia mouse model TKM-005-EF, 2014. 29 January 2015.

44. Pharmaseed_Ltd. Angiogenesis efficacy of TM-700 in the rat hind-limb ischemia model TKM-004-EF, 2014. 4 May 2015. 49.

45. Dupuis L, Pradat PF, Ludolph AC, Loeffler JP. Energy metabolism in amyotrophic lateral sclerosis. Lancet Neurol. 2011;10(1):75-82.

SAMPLE

46. Guo Z, Kindy MS, Kruman I, Mattson MP. ALS-linked Cu/Zn-SOD mutation impairs cerebral synaptic glucose and glutamate transport and exacerbates ischemic brain injury. J Cereb Blood Flow Metab. 2000;20(3):463-8.

47. Waldemar G, Vorstrup S, Jensen TS, Johnsen A, Boysen G. Focal reductions of cerebral blood flow in amyotrophic lateral sclerosis: a [99mTc]-d,l-HMPAO SPECT study. J Neurol Sci. 1992;107(1):19-28.

48. Zhong Z, Deane R, Ali Z, Parisi M, Shapovalov Y, O'Banion MK, et al. ALS-causing SOD1 mutants generate vascular changes prior to motor neuron degeneration. Nat Neurosci. 2008;11(4):420-2.

49. Organ SL, Tsao MS. An overview of the c-MET signaling pathway. Ther Adv Med Oncol. 2011;3(1 Suppl):S7-S19.

50. Bhaskar S, Bradley S, Israeli-Korn S, Menon B, Chattu VK, Thomas P, et al. Chronic Neurology in COVID-19 Era: Clinical Considerations and Recommendations From the REPROGRAM Consortium. Front Neurol. 2020;11:664.

51. Miyazaki K, Masamoto K, Morimoto N, Kurata T, Mimoto T, Obata T, et al. Early and progressive impairment of spinal blood flow-glucose metabolism coupling in motor neuron degeneration of ALS model mice. J Cereb Blood Flow Metab. 2012;32(3):456-67.

52. Atassi N, Berry J, Shui A, Zach N, Sherman A, Sinani E, et al. The PRO-ACT database: design, initial analyses, and predictive features. Neurology. 2014;83(19):1719-25.

53. Cudkowicz ME, van den Berg LH, Shefner JM, Mitsumoto H, Mora JS, Ludolph A, et al. Dexpramipexole versus placebo for patients with amyotrophic lateral sclerosis (EMPOWER): a randomised, double-blind, phase 3 trial. Lancet Neurol. 2013;12(11):1059-67.

54. Epton J, Harris R, Jenkinson C. Quality of life in amyotrophic lateral sclerosis/motor neuron disease: a structured review. Amyotroph Lateral Scler. 2009;10(1):15-26.

55. Jenkinson C, Levvy G, Fitzpatrick R, Garratt A. The amyotrophic lateral sclerosis assessment questionnaire (ALSAQ-40): tests of data quality, score reliability and response rate in a survey of patients. J Neurol Sci. 2000;180(1-2):94-100.

56. Jenkinson C, Peto V, Jones G, Fitzpatrick R. Interpreting change scores on the Amyotrophic Lateral Sclerosis Assessment Questionnaire (ALSAQ-40). Clin Rehabil. 2003;17(4):380-5.

57. Miller RG, Rosenberg JA, Gelinas DF, Mitsumoto H, Newman D, Sufit R, et al. Practice parameter: the care of the patient with amyotrophic lateral sclerosis (an evidence-based review): report of the Quality Standards Subcommittee of the American Academy of Neurology: ALS Practice Parameters Task Force. Neurology. 1999;52(7):1311-23.

SAMPLE

APPENDIX 1 – ALS FUNCTIONAL RATING SCALE REVISED (ALSFRS-R)

ALS Functional Rating Scale Revised (ALS-FRS-R) Version: May 2015

As described in:

Cedarbaum JM, Stambler N, Malta E, Fuller C, Hilt D, Thurmond B, Nakanishi A. The ALSFRS-R: a revised ALS functional rating scale that incorporates assessments of respiratory function. BDNF ALS Study Group (Phase III). J Neurol Sci. 1999 Oct 31;169(1-2):13-21. doi: 10.1016/s0022-510x(99)00210-5. PMID: 10540002.

SAMPLE

APPENDIX 2 - WMA DECLARATION OF HELSINKI

WORLD MEDICAL ASSOCIATION DECLARATION OF HELSINKI

Recommendations guiding physicians in biomedical research involving human subjects

Adopted by the 18th World Medical Assembly Helsinki, Finland, June 1964and amended by the29th World Medical Assembly, Tokyo, Japan, October 1975, 35th World Medical Assembly, Venice, Italy, October 1983, 41st World Medical Assembly, Hong Kong, September 1989 and the 48th General Assembly, Somerset West, Republic of South Africa, October 1996

INTRODUCTION

It is the mission of the physician to safeguard the health of the people. His or her knowledge and conscience are dedicated to the fulfilment of this mission.

The Declaration of Geneva of the World Medical Association binds the physician with the words, "The Health of my patient will be my first consideration," and the International Code of Medical Ethics declares that, "A physician shall act only in the patient's interest when providing medical care which might have the effect of weakening the physical and mental condition of the patient."

The purpose of biomedical research involving human subjects must be to improve diagnostic, therapeutic and prophylactic procedures and the understanding of the aetiology and pathogenesis of disease.

In current medical practice most diagnostic, therapeutic or prophylactic procedures involve hazards. This applies especially to biomedical research.

Medical progress is based on research which ultimately must rest in part on experimentation involving human subjects.

In the field of biomedical research a fundamental distinction must be recognized between medical research in which the aim is essentially diagnostic or therapeutic for a patient, and medical research, the essential object of which is purely scientific and without implying direct diagnostic or therapeutic value to the person subjected to the research.

Special caution must be exercised in the conduct of research which may affect the environment, and the welfare of animals used for research must be respected.

Because it is essential that the results of laboratory experiments be applied to human beings to further scientific knowledge and to help suffering humanity, the World Medical Association has prepared the following recommendations as a guide to every physician in biomedical research involving human subjects. They should be kept under review in the future. It must be stressed that the standards as drafted are only a guide to physicians all over the world. Physicians are not relieved from criminal, civil and ethical responsibilities under the laws of their own countries.

I. BASIC PRINCIPLES

1. Biomedical research involving human subjects must conform to generally accepted scientific principles and should be based on adequately performed laboratory and animal experimentation and on a thorough knowledge of the scientific literature.

2. The design and performance of each experimental procedure involving human subjects should be clearly formulated in an experimental protocol which should be transmitted for consideration, comment and guidance to a specially appointed committee independent of the investigator and the sponsor provided that this independent committee is in conformity with the laws and regulations of the country in which the research experiment is performed.

3. Biomedical research involving human subjects should be conducted only by scientifically qualified persons and under the supervision of a clinically competent medical person. The responsibility for

SAMPLE

the human subject must always rest with a medically qualified person and never rest on the subject of the research, even though the subject has given his or her consent.

4. 4. Biomedical research involving human subjects cannot legitimately be carried out unless the importance of the objective is in proportion to the inherent risk to the subject.

5. Every biomedical research project involving human subjects should be preceded by careful assessment of predictable risks in comparison with foreseeable benefits to the subject or to others. Concern for the interests of the subject must always prevail over the interests of science and society.

6. The right of the research subject to safeguard his or her integrity must always be respected. Every precaution should be taken to respect the privacy of the subject and to minimize the impact of the study on the subject's physical and mental integrity and on the personality of the subject.

7. Physicians should abstain from engaging in research projects involving human subjects unless they are satisfied that the hazards involved are believed to be predictable. Physicians should cease any investigation if the hazards are found to outweigh the potential benefits.

8. In publication of the results of his or her research, the physician is obliged to preserve the accuracy of the results. Reports of experimentation not in accordance with the principles laid down in this Declaration should not be accepted for publication.

9. In any research on human beings, each potential subject must be adequately informed of the aims, methods, anticipated benefits and potential hazards of the study and the discomfort it may entail. He or she should be informed that he or she is at liberty to abstain from participation in the study and that he or she is free to withdraw his or her consent to participation at any time. The physician should then obtain the subject's freely-given informed consent, preferably in writing.

10. When obtaining informed consent for the research project the physician should be particularly cautious if the subject is in a dependent relationship to him or her or may consent under duress. In that case the informed consent should be obtained by a physician who is not engaged in the investigation and who is completely independent of this official relationship.

11. In case of legal incompetence, informed consent should be obtained from the legal guardian in accordance with national legislation. Where physical or mental incapacity makes it impossible to obtain informed consent, or when the subject is a minor, permission from the responsible relative replaces that of the subject in accordance with national legislation. Whenever the minor child is in fact able to give a consent, the minor's consent must be obtained in addition to the consent of the minor's legal guardian.

12. The research protocol should always contain a statement of the ethical considerations involved and should indicate that the principles enunciated in the present Declaration are complied with.

II. MEDICAL RESEARCH COMBINED WITH PROFESSIONAL CARE

(Clinical Research)

1. In the treatment of the sick person, the physician must be free to use a new diagnostic and therapeutic measure, if in his or her judgement it offers hope of saving life, re-establishing health or alleviating suffering.

2. The potential benefits, hazards and discomfort of a new method should be weighed against the advantages of the best current diagnostic and therapeutic methods.

3. In any medical study, every patient - including those of a control group, if any - should be assured of the best proven diagnostic and therapeutic method. This does not exclude the use of inert placebo in studies where no proven diagnostic or therapeutic method exists.

SAMPLE

4. The refusal of the patient to participate in a study must never interfere with the physician-patient relationship.

5. If the physician considers it essential not to obtain informed consent, the specific reasons for this proposal should be stated in the experimental protocol for transmission to the independent committee (I, 2).

6. The physician can combine medical research with professional care, the objective being the acquisition of new medical knowledge, only to the extent that medical research is justified by its potential diagnostic or therapeutic value for the patient.

III. NON-THERAPEUTIC BIOMEDICAL RESEARCH INVOLVING HUMAN SUBJECTS (Non-Clinical Biomedical Research)

7. In the purely scientific application of medical research carried out on a human being, it is the duty of the physician to remain the protector of the life and health of that person on whom biomedical research is being carried out.

8. The subject should be volunteers - either healthy persons or patients for whom the experimental design is not related to the patient's illness.

9. The investigator or the investigating team should discontinue the research if in his/her or their judgement it may, if continued, be harmful to the individual.

10. In research on man, the interest of science and society should never take precedence over considerations related to the wellbeing of the subject.

SAMPLE

APPENDIX 3 - DEFINITION OF ADVERSE EVENTS

Adverse Event

Any untoward medical occurrence in a patient or clinical trial subject administered a medicinal product and which does not necessarily have a causal relationship with this treatment.

Comment:

An AE can therefore be any unfavourable and unintended sign (including abnormal laboratory findings), symptom or disease temporally associated with the use of an investigational medicinal product, whether or not related to the investigational medicinal product.

Adverse Reaction

All untoward and unintended responses to an IMP related to any dose administered.

Comment:

An AE judged by either the reporting Investigator or Sponsor as having causal relationship to the IMP qualifies as an AR. The expression reasonable causal relationship means to convey in general that there is evidence or argument to suggest a causal relationship.

Serious Adverse Event

Any untoward medical occurrence or effect that at any dose:

- Results in death
- Is life-threatening*
- Requires hospitalisation** or prolongation of existing inpatients' hospitalisation
- Results in persistent or significant disability or incapacity
- Is a congenital anomaly/birth defect
- Or is otherwise considered medically significant by the Investigator***

Comments:

The term severe is often used to describe the intensity (severity) of a specific event. This is not the same as serious, which is based on patients/event outcome or action criteria.

* Life threatening in the definition of an SAE refers to an event in which the patient was at risk of death at the time of the event; it does not refer to an event that hypothetically might have caused death if it were more severe.

**Hospitalisation is defined as an unplanned, formal inpatient admission, even if the hospitalisation is a precautionary measure for continued observation. Thus, hospitalisation for protocol treatment (e.g. line insertion), elective procedures (unless brought forward because of worsening symptoms) or for social reasons (e.g. respite care) are not regarded as an SAE.

*** Medical judgment should be exercised in deciding whether an AE is serious in other situations. Important AEs that are not immediately life threatening or do not result in death or hospitalisation but may jeopardise the subject or may require intervention to prevent one of the other outcomes listed in the definition above, should be considered serious.

Serious Adverse Reaction

An Adverse Reaction which also meets the definition of a Serious Adverse Event.

Suspected Unexpected Serious Adverse Reaction

A SAR that is unexpected i.e. the nature, or severity of the event is not consistent with the applicable product information.

SAMPLE

A SUSAR should meet the definition of an AR, UAR and SAR.

Unexpected Adverse Reaction

An AR, the nature or severity of which is not consistent with the applicable product information (e.g. Investigator Brochure for an unapproved IMP or (compendium of) Summary of Product Characteristics (SPC) for a licensed product).

When the outcome of an AR is not consistent with the applicable product information the AR should be considered unexpected.

SAMPLE

APPENDIX 4 - COMMON TOXICITY CRITERIA GRADINGS

Toxicities will be recorded according to the Common Terminology Criteria for Adverse Events (CTCAE), version 4.0 (4.03 corrected). The full CTCAE document is available on the National Cancer Institute (NCI) website.

SAMPLE

APPENDIX 5 – LIVER SAFETY REQUIRED ACTIONS AND FOLLOW-UP ASSESSMENTS

Phase II liver chemistry increased monitoring criteria have been designed to assure subject safety and evaluate liver event aetiology (in alignment with the FDA premarketing clinical liver safety guidance). http://www.fda.gov/downloads/Drugs/GuidanceComplianceRegulatoryInformation/Guidances/UCM174090.pdf.

**Phase II liver chemistry increased monitoring criteria and required follow up assessments Liver Chemistry Increased Monitoring Criteria**	
**ALT-absolute**	ALT ≥ 5xULN
**ALT Increase**	ALT ≥ 3xULN persists for 4 weeks
**Bilirubin1, 2**	ALT > 3xULN **and** bilirubin 2xULN (>35% direct bilirubin)
**INR2**	ALT ≥ 3xULN **and** INR>1.5, if INR measured
**Cannot Monitor**	ALT ≥ 3xULN and cannot be monitored weekly for 4 weeks
**Symptomatic3**	ALT ≥ 3xULN associated with symptoms (new or worsening) believed to be related to liver injury or hypersensitivity
**Required Actions and Follow up Assessments following ANY Liver Event**	
**Actions**	**Follow Up Assessments**
• Report the event to Trials Office **within 72 hours**<br>• Complete the liver event CRF and complete an SAE - if the event also meets the criteria for an SAE<br>• Perform liver event follow up assessments<br>• Monitor the subject until liver chemistries resolve, stabilize, or return to within baseline (see **MONITORING** below). "Baseline" refers to the laboratory assessments performed closest and prior to dosing of study treatment.<br>**MONITORING:**<br>**For bilirubin or INR criteria:**<br>• Repeat liver chemistries (include ALT, AST, alkaline phosphatase, bilirubin) and perform liver event follow up assessments within 24 hrs	• Viral hepatitis serology[4]<br>• Blood sample for pharmacokinetic (PK) analysis, obtained within 7after an identified liver event if within 6 months post dose.5<br>• Serum creatine phosphokinase (CPK) and lactate dehydrogenase (LDH).<br>• Fractionate bilirubin, if total bilirubin 2xULN<br>• Obtain complete blood count with differential to assess eosinophilia<br>• Record the appearance or worsening of clinical symptoms of liver injury, or hypersensitivity, on the AE report form

SAMPLE

• Monitor subjects twice weekly until liver chemistries resolve, stabilize or return to within baseline<br>• A specialist or hepatology consultation is recommended<br><br>For All other criteria:<br>• Repeat liver chemistries (include ALT, AST, alkaline phosphatase, bilirubin) and perform liver event follow up assessments within 24-72 hrs<br>• Monitor subjects weekly until liver chemistries resolve, stabilize or return to within baseline	• Record use of concomitant medications on the concomitant medications report form including acetaminophen, herbal remedies, other over the counter medications.<br>• Record alcohol use on the liver event alcohol intake case report form<br>For bilirubin or INR criteria:<br>• Anti-nuclear antibody, anti-smooth muscle antibody, Type 1 anti-liver kidney microsomal antibodies, and quantitative total immunoglobulin G (IgG or gamma globulins).<br>• Serum acetaminophens adduct HPLC assay (quantifies potential acetaminophen contribution to liver injury in subjects with definite or likely acetaminophen use in the preceding week [James, 2009]).<br>• Liver imaging (ultrasound, magnetic resonance, or computerised tomography) and /or liver biopsy to evaluate liver disease; complete Liver Imaging and/or Liver Biopsy CRF forms.

1. Serum bilirubin fractionation should be performed if testing is available. If serum bilirubin fractionation is not immediately available, discontinue study treatment for that subject if ALT 3xULN **and** bilirubin 2xULN. Additionally, if serum bilirubin fractionation testing is unavailable, **record presence of detectable urinary bilirubin on dipstick**, indicating direct bilirubin elevations and suggesting liver injury.

2. All events of ALT ≥ 3xULN **and** bilirubin ≥ 2xULN (>35% direct bilirubin) or ALT ≥ 3xULN **and** INR>1.5, if INR measured which may indicate severe liver injury (possible 'Hy's Law'), **must be reported as an SAE (excluding studies of hepatic impairment or cirrhosis)**; INR measurement is not required and the threshold value stated will not apply to subjects receiving anticoagulants

3. New or worsening symptoms believed to be related to liver injury (such as fatigue, nausea, vomiting, right upper quadrant pain or tenderness, or jaundice) or believed to be related to hypersensitivity (such as fever, rash or eosinophilia)

4. Includes: Hepatitis A IgM antibody; Hepatitis B surface antigen and Hepatitis B Core Antibody (IgM); Hepatitis C RNA; Cytomegalovirus IgM antibody; Epstein-Barr viral capsid antigen IgM antibody (or if unavailable, obtain heterophile antibody or monospot testing); Hepatitis E IgM antibody

5. Record the date/time of the PK blood sample draw and the date/time of the last dose of study treatment prior to blood sample draw on the CRF. If the date or time of the last dose is unclear, provide the subject's best approximation. If the date/time of the last dose cannot be approximated OR a PK sample cannot be collected in the time period indicated above, do not obtain a PK sample.

SAMPLE

APPENDIX 6 – ALS ASSESSMENT QUESTIONNAIRE (ALSAQ-40)

Please visit the following website for details about the ALSAQ-40:

https://innovation.ox.ac.uk/outcome-measures/amyotrophic-lateral-sclerosis-assessment-questionnaire-alsaq/

The research commercialisation office of the University of Oxford, previously called **Isis Innovation**, has been renamed **Oxford University Innovation**

All documents and other materials will be updated accordingly. In the meantime the remaining content of this Isis Innovation document is still valid.

URLs beginning www.isis-innovation.com/... are automatically redirected to our new domain, www.innovation.ox.ac.uk/...

Phone numbers and email addresses for individual members of staff are unchanged

Email: enquiries@innovation.ox.ac.uk

SAMPLE

APPENDIX 7 – EQUATION FOR ESTIMATED GLOMERULAR FILTRATION RATE

COCKCROFT-GAULT

Creatinine clearance (estimated GFR) = $\frac{([(140 - \text{age in years}) \times (\text{wt in kg})] \times 1.23)}{(\text{serum creatinine in micromol/l})}$

SAMPLE

APPENDIX 8 – HMA CTFG GUIDELINES ON CONTRACEPTION

Birth control methods which may be considered as highly effective

For the purpose of this guidance, methods that can achieve a failure rate of less than 1% per year when used consistently and correctly are considered as highly effective birth control methods. Such methods include:

- combined (oestrogen and progestogen containing) hormonal contraception associated with inhibition of ovulation[1]
    - o oral
    - o intravaginal
    - o transdermal
- progestogen-only hormonal contraception associated with inhibition of ovulation[1]:
    - o oral
    - o injectable
    - o implantable[2]
- intrauterine device (IUD)[2]
- intrauterine hormone-releasing system (IUS)[2]
- bilateral tubal occlusion[2]
- vasectomised partner[2,3]
- sexual abstinence[4]

Acceptable birth control methods which may not be considered as highly effective

Acceptable birth control methods that result in a failure rate of more than 1% per year include:

- progestogen-only oral hormonal contraception, where inhibition of ovulation is not the primary mode of action
- male or female condom with or without spermicide
- cap, diaphragm or sponge with spermicide[5]

Assessment of pharmacokinetic interaction between the IMP and hormonal Contraceptives and recommendations on the use of hormonal contraceptives

For hormonal contraception methods, caution should be taken to possible interaction with a (Non-biologic) IMP. Interaction with the IMP leading to reduced efficacy of the hormonal contraception method can occur due to e.g. increased metabolism (enzyme induction).

Birth control methods which are considered unacceptable in clinical trials

Periodic abstinence (calendar, symptothermal, post-ovulation methods), withdrawal (coitus interruptus), spermicides only, and lactational amenorrhoea method (LAM) are not acceptable methods of contraception. Female condom and male condom should not be used together.

SAMPLE

[1] Hormonal contraception may be susceptible to interaction with the IMP, which may reduce the efficacy of the contraception method (see Section xx)

[2] Contraception methods that in the context of this guidance are considered to have low user dependency.

3 Vasectomised partner is a highly effective birth control method provided that partner is the sole sexual partner of the WOCBP trial participant and that the vasectomised partner has received medical assessment of the surgical success.

4 In the context of this guidance sexual abstinence is considered a highly effective method only if defined as refraining from heterosexual intercourse during the entire period of risk associated with the study treatments. The reliability of sexual abstinence needs to be evaluated in relation to the duration of the clinical trial and the preferred and usual lifestyle of the subject.

5 A combination of male condom with either cap, diaphragm or sponge with spermicide (double barrier methods) are also considered acceptable, but not highly effective, birth control methods

SAMPLE

fyi... for your information

APPENDIX 9 – EL ESCORIAL WORLD FEDERATION OF NEUROLOGY CRITERIA FOR THE DIAGNOSIS OF ALS

SAMPLE

Criteria for the diagnosis of Amyotrophic Lateral Sclerosis

The diagnoses of ALS requires the presence of

1) Signs of lower motor neuron (LMN) degeneration by clinical, electrophysiological or neuropathologic examination,
2) Signs of upper motor neuron (UMN) degeneration by clinical examination, and
3) Progressive spread of signs within a region or to other regions, together with the absence of
4) Electrophysiological evidence of other disease processes that might explain the signs of LMN and/or UMN degenerations; and
5) Neuroimaging evidence of other disease processes that might explain the observed clinical and electrophysiological signs.

Steps in the diagnosis of Amyotrophic Lateral Sclerosis

The diagnoses of ALS is made possible by

1) History, physical and appropriate neurological examinations to ascertain clinical finding which may suggest suspected, possible, probable or definite ALS,
2) Electrophysiological examinations to ascertain findings which confirm LMN degeneration in clinically involved regions, identify LMN degeneration in clinically uninvolved regions and exclude other disorders,
3) Neuroimaging examinations to ascertain findings which may exclude other disease processes,
4) Clinical laboratory examinations, determined by clinical judgment, to ascertain possible ALS-related syndromes,
5) Neuropathologic examinations, where appropriate, to ascertain findings which may confirm or exclude sporadic ALS, coexistent sporadic ALS, ALS-related syndromes or ALS variants,
6) Repetition of clinical and electrophysiological examinations at least six months apart to ascertain evidence of progression.

Clinical features in the diagnosis of ALS

Patients with signs of LMN degeneration (weakness, atrophy and clinical fasciculation's) and UMN degeneration (spasticity, pathologic reflexes, etc.) may be suspected as having ALS. Careful history,

The ALS Association, 27001 Agoura Road, Suite 250, Calabasas Hills, CA 91301-5104,
Phone: (800) 782-4747 / alsinfo@alsa-national.org / www.alsa.org

fyi... for your information

physical and neurological examination must search for further clinical evidence of LMN and UMN signs in four regions of the central nervous system.

Clinical features required for the diagnosis of ALS

1) Signs of LMN degeneration (weakness, wasting and fasciculation) in one or more of the four regions (bulbar, cervical, thoracic, lumbosacral). LMN findings in a region are without regard to right or left, but

   are indicative of the level of neuraxis involved. Therefore, spread of weakness, wasting and fasciculation's to another region is more important than spread from right to left or vice-versa.
2) Signs of UMN degeneration (increased or donic tendon reflexes, spasticity, pseudo bulbar features, Hoffmann reflex and extensor plantar response) in one or more of the four regions. These UMN signs are clinically appreciated best in the bulbar, cervical and lumbosacral regions. UMN findings in a region are also without regard to right or left. Once the physical and neurological examinations provide information on the presence or absence of LMN and UMN signs in the four regions (bulbar, cervical, thoracic, lumbosacral) they must be ordered topographically in the manner to determine the certainty of the diagnosis of ALS.
3) The topographical location of certain UMN and LMN signs in four regions of the CNS together with progression of these signs determines the certainty of the diagnoses of ALS. Progression is a cardinal feature of the clinical diagnosis of ALS. Progression of signs within a region and progression of signs to involve other regions are crucial to the diagnosis.

SAMPLE

Clinical examinations should be repeated at least every six (6) months to assess progression.

Cases which meet the topographical criteria for probable or definite ALS but which lack progression during the twelve (12) month period diagnosis should be designated as possible ALS.

Definite ALS

is defined on clinical grounds alone by the presence of UMN as well as LMN signs in the bulbar region and at least two of the other spinal regions or the presence of UMN and LMN signs in three spinal regions. The important determinants of diagnosis of definite ALS in the absence of electrophysiological, neuroimaging and laboratory examinations are the presence of UMN and LMN signs together in multiple regions.

The ALS Association, 27001 Agoura Road, Suite 250, Calabasas Hills, CA 91301-5104,
Phone: (800) 782-4747 / alsinfo@alsa-national.org / www.alsa.org

fyi... for your information

SAMPLE

Probable ALS

is defined on clinical grounds alone by UMN and LMN signs in at least two regions. While the regions may be different, some UMN signs must be rostral (above) the LMN signs. Multiple different combinations of UMN and LMN signs may be present in patients with probable ALS.

Possible ALS

is defined on clinical grounds alone when the UMN and LMN signs are in only one region or UMN signs alone are present in 2 or more regions or LMN signs are rostral to UMN signs (the latter distribution of signs needs to be differentiated from multiple non-ALS processes). Monomelic ALS, progressive bulbar palsy without spinal UMN and/or LMN signs and progressive primary lateral sclerosis without spinal LMN signs and progressive primary lateral sclerosis without spinal LMN signs constitute special cases which may develop LMN or UMN signs to meet the criteria for probable ALS with time or be subsequently confirmed at autopsy by specific LMN and UMN neuropathologic findings.

Suspected ALS

will manifest only LMN signs in 2 or more regions, although UMN pathology might be demonstrated at autopsy. However, only clinical signs are considered pertinent to this classification at the time of diagnostic evaluation.

Supportive clinical features

Clinical features that support the diagnosis of ALS include one or more of the following:

1) abnormal pulmonary function test not explained by other causes,
2) abnormal speech studies not explained by other causes,
3) abnormal swallowing studies not explained by other causes,
4) abnormal larynx function studies not explained by other causes,
5) abnormal isokinetic or isometric strength test in clinically uninvolved muscles,
6) abnormal muscle biopsy with evidence of denervation.

Inconsistent clinical features

Clinical findings inconsistent with the diagnoses of ALS include one or more of the following not explained by physiological changes associated with aging or other disease processes:

The ALS Association, 27001 Agoura Road, Suite 250, Calabasas Hills, CA 91301-5104,
Phone: (800) 782-4747 / alsinfo@alsa-national.org / www.alsa.org

fyi... for your information

1) sensory dysfunction,
2) sphincter abnormalities,
3) autonomic nervous system dysfunction,
4) anterior visual pathway abnormalities,
5) movement abnormalities associated with probable Parkinson's disease defined by DATATOP criteria,
6) cognitive abnormalities associated with clinical Alzheimer's disease as defined by NINCDS-ADRDA criteria.

If these clinical findings occur, then close attention should be paid to the possible diagnosis of other disease processes.

Lower motor neuron and upper motor neuron signs may occur together with other clinical signs in disease where the pathologic process is not primary motor neuron degeneration.

Types of ALS

The clinical signs of progressive LMN and UMN degeneration seen in ALS may

a) occur alone (sporadic ALS),
b) be present incidentally with other pre-existing disease processes that have not developed in parallel with the ALS (coexistent sporadic ALS),
c) Occur in association with laboratory-defined or epidemiologically defined abnormalities that are time- linked to the ALS (ALS-related syndromes), or
d) Occur in association with clinical, genetic or epidemiological features which develop in parallel with the ALS (ALS variants).

The physical and neurological examinations will allow for the clinical diagnosis of ALS to a particular degree of certainty as defined above; however, the history of the disease onset, toxic exposures, past medical history, injuries, family history, geographic location, etc., must be incorporated with the clinical examinations in determining whether the patient may have an ALS related syndrome or an ALS variant.

ALS-related syndromes must meet the clinical, electrophysiological and neuroimaging criteria for possible, probable or definite ALS. ALS-related syndromes have unique laboratory-defined or epidemiologically defined features which are time-linked to the development of the ALS phenotype. If correction of the associated laboratory feature does not result in correction of the ALS phenotype, then the patient with an ALS-related syndrome should be considered in the same way as a patient with sporadic ALS.

SAMPLE

fyi... *for your information*

ALS-related syndromes include

1) Monoclonal gammopathy (monoclonal gammopathy of unknown significance, Waldenstroms's macroglobulinemia, osteosclerotic myeloma, etc.),
2) Dysimmune motor system degeneration (autoimmune; high-titer GMI ganglioside antibody; etc.),
3) Nonmalignant endocrine abnormalities (hyperthyroidism, hyperparathyroidism, hypogonadism, etc.),
4) Lymphoma (Hodgkin's and non-Hodgkin's lymphoma). Cases of sporadic ALS associated with insulinoma, lung, colon or thyroid cancer are thought not to be casually related,
5) Infection (HIV-1, HTLV-I, encephalitis lethargica, varicella-zoster, brucellosis, cat-scratch disease, Creutzfeldt-Jakob disease, syphilis, delayed post-poliomyelitis, etc.),
6) Acquired enzyme defects (detoxification enzymes, etc.),
7) Exogenous toxins (lead, mercury, arsenic, thallium, cadmium, manganese, aluminum, organic pesticides, lupin seeds, etc.),
8) Physical injury (electric shock, radiation therapy, etc.),
9) Vascular (vasculitis; ischemic (Dejerine anterior bulbar artery syndrome, etc.),
10) Spondylotic myelopathy (painless myelopathy with no sensory signs, stabilization or progression post- surgery).

ALS Variants must meet the clinical, electrophysiological and neuroimaging criteria for possible, probable or definite ALS. The predominant presentation is that seen in sporadic ALS, but includes one or more features such as:

1) Familial pattern of inheritance (multiple phenol-types characterized by age of onset; site of onset; length of survival; and presumed type of inheritance.)

Familial ALS variants in genetic linkage studies should be characterized by an established genetic mode of inheritance over at least two generations and at least one clinically definite or autopsy confirmed case and compelling evidence excluding other possible causes. Affected sub pairs occurring in one generation alone may not result from a single gene effect.

Examples:

a) ALS with defined inheritance and known gene product (hexosaminidase A/B deficiency, superoxide dismutase deficiency)
b) ALS with defined inheritance and chromosome linkage but no gene product (chromosome 21 associated familial ALS or chromosome 2 associated juvenile familial ALS)

The ALS Association, 27001 Agoura Road, Suite 250, Calabasas Hills, CA 91301-5104,
Phone: (800) 782-4747 / alsinfo@alsa-national.org / www.alsa.org

SAMPLE

   c) ALS with defined inheritance and no known linkage or gene product (most cases or familial ALS).
2) Geographic clustering (including disorders seen in the Western Pacific, Guam, Kii Peninsula, North Africa, Madras, etc.)
3) Extrapyramidal signs (bradykinesia; cogwheel rigidity; tremor; clinically significant onset of supranuclear eye signs (pursuit abnormalities); familial or sporadic)
4) Cerebellar degeneration (spinocerebellar abnormalities; familial or sporadic)
5) Dementia (progressive cognitive abnormalities; familial or sporadic)
6) Autonomic nervous system involvement (clinically significant abnormal cardiovascular reflexes; bowel or bladder control problems; familial or sporadic)
7) Objective sensory abnormalities (decreased vibration; sharp-dull discrimination; blunting of cold sensation; familial or sporadic)

Electrophysiological features in the diagnoses of ALS

Patients with suspected, possible, probable or definite ALS on clinical grounds should have electrophysiological studies performed to confirm LMN degeneration in clinically affected regions, find electrophysiological evidence of LMN degeneration in clinically uninvolved regions and to exclude other pathophysiological processes.

ALS may be most reliably identified when the clinical and electrophysiological findings are widespread, involving a sufficient number of regions so that other possible cause of similar EMG abnormalities are highly unlikely. The confirmation of the diagnosis of ALS depends on finding electrophysiological evidence of LMN degeneration in at least two muscles of different root or spinal nerves and different cranial or peripheral nerve innervation in two or more of the four (bulbar, cervical, thoracic, lumbosacral) regions. The features of LMN degeneration in a particular muscle are defined by electromyographic needle examination and nerve conduction studies using standard methods for each measure.

Electrophysiological features required to identify definite primary LMN degeneration include all of the following:

1) Reduced recruitment (reduced interference pattern with firing rates over 10 Hz),
2) Large motor unit action potentials (large amplitude, long duration), and
3) Fibrillation potentials.

Electrophysiological features that support the identification of possible primary LMN degeneration include one or more of the following:

SAMPLE

fyi... for your information

1) either reduced recruitment, large motor unit potentials, fibrillation potentials or unstable motor unit potentials alone,
2) polyphasic motor unit potentials or increased single fiber density alone,
3) low amplitude compound muscle actions potentials if the disease duration is over 5 years or if there is associated atrophy,
4) low amplitude compound muscle action potentials,
5) compound muscle action potential change between proximal and distal sites of stimulation that is uniform along the length of the nerve,
6) up to 30% decrement in motor conduction velocity below established normal values if a low amplitude compound muscle action potential greater than 10 percent of normal is present,
7) up to 50% decrement in motor conduction velocity below established normal values if the compound muscle action potential is below 10% or normal,,
8) up to 20% decrement of the compound muscle action potential on 2 Hz repetitive stimulation,
9) up to 10% decrement in sensory nerve conduction velocity and action potential amplitude from established normal values,
10) complex repetitive discharges, and
11) absence of fasciculations.

Electrophysiological features compatible with UMN degeneration and not excluding ALS include one or more of the following:

1) up to 30% increment in central motor conduction velocity,
2) up to 10% decrement in somatosensory, evoked potential amplitude and up to 10% increment in somatosensory evoked potential latency,
3) mild abnormalities of autonomic function,
4) mild abnormalities of polysomnography,
5) mild abnormalities of electronystagmography.

Electrophysiological features that are inconsistent with the diagnoses of ALS or suggest the presence of additional other disease processes include one or more of the following:

1) focal reduction in compound muscle action potential or more than 10% in a 4-cm segment,
2) motor conduction velocities, F wave latencies or H wave amplitudes which are more than 30% above established normal values,
3) more than 20% decrement of repetitive stimulation at 2 Hz,
4) sensory action potential latencies more than 20% above or sensory action potential amplitudes more than 20% below established normal values,

The ALS Association, 27001 Agoura Road, Suite 250, Calabasas Hills, CA 91301-5104,
Phone: (800) 782-4747 / alsinfo@alsa-national.org / www.alsa.org

fyi... for your information

5) unstable motor unit potentials with no other electromyographic changes,
6) more than 30% increment of central motor conduction velocity,
7) more than 10% increment in sensory evoked potential latency or more than 10% decrement in sensory evoked potential amplitude,
8) moderate or greater abnormalities in autonomic function or electronystagnography.

Employing electrophysiological evidence of LMN degeneration to confirm the diagnosis of ALS

The certainty of LMN degeneration is determined by the presence of the above finding for each muscle tested in the region.

At least two muscles of different root or spinal nerve and different cranial or peripheral nerve inneveration in each region should show electrophysiological evidence of either definite, probable or possible LMN degeneration for that region to be ranked as showing definite, probable or possible LMN degeneration.

Definite LMN degeneration by EMG has the same significance as clinical LMN degeneration and can upgrade the certainty of the clinical diagnoses of ALS in the same fashion as if the clinical signs of LMN degeneration were present in that region.

Probable or possible LMN degeneration by EMG does not carry the same weight as either clinical signs of definite electrophysiological evidence of LMN degeneration in a particular region.

However, the involvement of the regions with probable electrophysiological evidence of LMN degeneration or one region with probable and one region with possible electrophysiological evidence of LMN degeneration carries the same weight as one region with definite evidence of LMN degeneration in upgrading the certainty of diagnosis of ALS.

A single region with electrophysiological evidence of probable LMN degeneration or two regions with electrophysiological evidence of possible LMN degeneration can be used to upgrade the certainty of the diagnosis of ALS from possible ALS to probable ALS but not from probable ALS to definite ALS.

Neuroimaging features in the diagnosis of ALS

Neuroimaging studies should be selected in order to exclude other conditions which may cause UMN and/or LMN signs that may stimulate sporadic ALS.

The ALS Association, 27001 Agoura Road, Suite 250, Calabasas Hills, CA 91301-5104,
Phone: (800) 782-4747 / alsinfo@alsa-national.org / www.alsa.org

SAMPLE

fyi... for your information

Neuroimaging features required for the diagnosis of ALS:

- There are no neuroimaging tests which confirm the diagnosis of ALS.

Neuroimaging features that support the diagnosis of ALS include one or more of the following:

1) minimal bony abnormalities on plain x-ray of skull or spinal canal,
2) Minimal abnormalities on head or spinal cord MRI scans without spinal cord and/or root compression,
3) Minimal abnormalities on spinal cord myelography with post-myelography CT tomography showing no spinal cord and/or root compression.

Neuroimaging features that are inconsistent with the diagnosis of ALS include one or more of the following:

1) significant bony abnormalities on plain x-ray of skull or spinal canal,
2) minimal abnormalities on head or spinal cord MRI scans without spinal cord and/or root compression,
3) minimal abnormalities on spinal cord myelography with post-myelography CT tomography showing no spinal cord and/or room compression.

Neuroimaging features that are inconsistent with the diagnosis of ALS include one or more of the following:

1) significant bony abnormalities on plain x-rays of skull or spinal canal that might explain clinical findings,
2) significant abnormalities of head or spinal cord MRI suggesting intraparenchymal processes, arteriovenous malformations or compression of brainstem/spinal cord and/or cranial nerve or spinal nerve roots by bony abnormalities, tumor, etc. MRI of craniocervical function if bulbar onset and/or MRI or pertinent spinal region if spinal onset,
3) significant abnormalities of spinal cord myelography with/without CT tomography or CT tomography alone suggesting lesions as noted above,
4) significant abnormalities on spinal cord angiography suggesting arteriovenous malformations.

Employing neuroimaging evidence to confirm the diagnosis of ALS

The absence of abnormalities in appropriately performed neuroimaging studies will raise patients with clinical and/or electrophysiological evidence of probable ALS to definite ALS.

The ALS Association, 27001 Agoura Road, Suite 250, Calabasas Hills, CA 91301-5104,
Phone: (800) 782-4747 / alsinfo@alsa-national.org / www.alsa.org

SAMPLE

fyi... for your information

The absence of neuroimaging abnormalities cannot raise possible ALS to probable ALS.

Clinical laboratory features in the diagnosis of ALS

The diagnostic process employed to confirm the diagnosis of sporadic ALS includes repeated clinical examinations, repeated electrophysiological examinations, neuroimaging to exclude other disorders and clinical laboratory examinations or exclude other disorders or support the diagnosis of ALS related syndromes.

Clinical laboratory features required for the diagnoses of ALS

- There are no clinical laboratory tests which confirm the diagnosis of ALS.

Clinical laboratory features that support the diagnosis of ALS include one or more of the following:

- normal complete blood count, platelet count, sedimentation rate, prothrombin time,
- normal electrolyte (Na+, K+, C1, C02+, Mg2+, P04) renal (BUN, creatinine) and liver function (bilirubin, SGOT, SGPT, LDH) test,
- creatine kinase (CK) elevation not more than 5 times upper limit of normal,
- normal hexosaminidase A and B activity (if possible of deficiency indicated by suggestive family history or onset under 30 years of age),
- normal cerebrospinal fluid cell count, protein (not more than 65 mg/dl), absence of intrathecal immunoglobulin synthesis, oligoclonal immunoglobulins and evidence of elevated intrathecal antibodies or infectious agents (syphilis, HIV-1, HTLV-I, etc.), if indicated,
- normal parathyroid hormone level if calcium is borderline elevated,
- normal free thyroid hormone concentrations if any thyroid function abnormalities
- (borderline elevations in T4, T3, TSH); normal glycosylated hemoglobin, if indicated,
- Normal serum protein electrophoresis and serum immunoeletrophresis with immunofixation; normal urine immunoelectrophoresis with immunofixation, if indicated,
- Minimal abnormalities in screening test for collagen vascular diseases (anti-nuclear antibody; anti-DNA antibodies; rheumatoid factor, complement, anti-tissue specific antibodies), if indicated,
- Minimal elevation in screening test for anti-neural antigen (GM1, GM2, GD1b gangliosides, myelin- associated glycoprotein, acetylcholine esterase, etc.) or anti-neuromuscular antigen (acetylcholine receptor, striated muscle, etc.) antibodies, if indicated.

The ALS Association, 27001 Agoura Road, Suite 250, Calabasas Hills, CA 91301-5104,
Phone: (800) 782-4747 / alsinfo@alsa-national.org / www.alsa.org

SAMPLE

fyi... for your information

Clinical laboratory features that support the diagnosis of ALS related syndromes

- Abnormalities consistent with monoclonal gammopathy with/without significant elevation in monoclonal anti-neural antigen antibody,
- Significant elevations in polyclonal anti-neural antigen (Gm1, Gm2, GD1b gagnliosides, myelin-associated glycoprotein, acetylcholine esterase, etc.) antibody,
- Significant elevation in parathyroid hormone, thyroid hormone or other significant endocrine abnormalities,
- Abnormalities consistent with lymphoma (Hodgkin's or non-Hodgkin's lymphoma),
- Evidence of infection (HIV-1, HTLV-I, borrelia, syphilis, brucellosis, cat-scratch disease, varicella-zoster, influenza, Creutzfeldt-Jakob disease),
- Evidence of intoxication (epidemiological evidence or elevated blood, urine, tissue or cerebrospinal fluid level of lead, mercury, arsenic, cadmium, manganese, aluminum, organic pesticides, lupin seeds, etc.),
- Evidence of physical injury (epidemiological evidence of antecedent electrical or radiation injury or severe trauma),
- Evidence of vasculitis (elevated erythrocyte sedimentation rate and cerebrospinal fluid abnormalities consistent with spinal cord vasculitis, i.e., markedly elevated cerebrospinal fluid protein) or ischemic injury to spinal cord without sensory signs,
- Evidence of pre-existing mild or moderate spinal cord spondylotic compression, not amenable to surgical correction or not responding to surgical correction, which progressed with clinical signs consistent with at least probable ALS.

Clinical laboratory features inconsistent with the diagnosis of ALS:

There is no clinical laboratory finding which, if present with the proper clinical and electrophysiological signs of ALS and appropriate neuroimaging studies, rules out the diagnosis of ALS.

ALS-related syndromes which present with the ALS phenotype as defined have been described with laboratory abnormalities (acquired or genetic), time-linked exposure to chemical, physical or infectious agents and pre-existing structural abnormalities. The correction of laboratory abnormalities, removal of chemical or physical agents, treatment of the associated disease (infection, tumor, structural abnormality) may or may not result in correction or stabilization of the ALS phenotype in ALS-related syndromes.

If correction of the abnormality does not result in improvement, the patient will be considered to have an ALS related syndrome.

The ALS Association, 27001 Agoura Road, Suite 250, Calabasas Hills, CA 91301-5104,
Phone: (800) 782-4747 / alsinfo@alsa-national.org / www.alsa.org

SAMPLE

fyi... for your information

If correction of the abnormality does not result in improvement, then the patient will be considered to have an ALS related syndrome.

If correction of the abnormality does not result in improvement then the patient will be considered to have sporadic ALS for the purpose of clinical studies and the therapeutic trials.

Neuropathological features of the diagnosis of ALS:

The clinical diagnosis may be supported or excluded by biopsy studies in the living patient and the pathological diagnosis may be proven or excluded by autopsy examination.

Pathological studies in the living patient with sporadic ALS:

Indications of biopsies:

Biopsies of the skeletal muscle, peripheral nerve and other tissues are not required for the diagnosis of amyotrophic lateral sclerosis, unless the clinical, electrophysiological or laboratory studies have revealed changes that are atypical for ALS. In addition, the muscle biopsy may be used to demonstrate LMN involvement in a body region that had not been shown to be involved by other techniques.

Muscle biopsy:

Features required for the diagnosis:
Disseminated single angulated atrophic muscle fibers, or small or large groups of such fibers.

Features that strongly support the diagnosis:
Angulated atrophic muscle fibers that are strongly positive when stained with oxidative enzyme stains and with non-specific esterase or that show immunoreactive surface staining with anti-NCAM antibodies.

Features that are compatible with, and do not exclude the diagnosis:

1) Scattered hypertrophied muscle fibers,,
2) No more than a moderate number of target or targeted fibers,,

SAMPLE

3) Fiber type grouping of not more than mild-to-moderate extent,,
4) The presence of a small number of necrotic muscle fibers.

Features that rule out the diagnoses or suggest the presence of additional disease:

1) Significant infiltration with lymphocytes and other mononuclear inflammatory cells,
2) Significant arteritis,
3) Significant numbers of muscle fibers involved with the following structural changes: necrosis; rimmed vacuoles; nemaline bodies; central cores; accumulation of mitochondria (ragged red fibers),
4) Large fiber type grouping,
5) Giant axonal swellings from accumulation of masses of neurofilaments, but not of PAS positive bodies, in intramuscular nerves.

Pathological studies at autopsy, other than in cases surviving for prolonged periods on life support systems

Gross pathological changes

Features required for the diagnosis:
There are positive diagnostic features on gross pathological examination.

Features that support the diagnosis:
There are no positive diagnostic features on gross pathological examination. Features that support the diagnosis:

1) selective atrophy of the motor cortex,
2) grayness and atrophy of the anterior spinal nerve roots compared with normal,
3) grayness of the lateral columns of the spinal cord,
4) atrophy of skeletal muscles.

Features that rule out the diagnosis of ALS or suggest the presence of additional disease:
1) Plaques of multiple sclerosis.
2) A focal cause of myelopathy. Light microscopic studies

Features required for the diagnosis:

SAMPLE

fyi…
for your information

1) Some degree of loss of both of the following neuronal systems. Large motor neurons of the anterior horns of the spinal cord and motor nuclei of the brainstem (V motor, VII motor, I and X somatic motor, and XII); and large pyramidal neurons of the motor cortex and/or large myelinated axons of the corticospinal tracts.
2) The following cellular pathological changes in the involved neuronal regions described above; neuronal atrophy with relative increase in lipofuscin and loss of Nissl substance. There should be evidence of different stages of the process of neuronal degeneration, including the presence of normal-appearing neurons, even in the same region.
3) Evidence of degeneration of the corticospinal tracts and the same level.

Features that strongly support the diagnosis:

1) Lack of pathological change in the motor neurons of cranial nerves III, IV and VI, the intermediolateral column of the spinal cord, and Onuf's nucleus.
2) The occurrence of one or more of the following cellular pathological changes in the involved neuronal systems described above:
    - Axonal spheroids with accumulation of masses of neurofilaments,
    - Bunina bodies,
    - Basophilic cytoplasmic inclusions,
    - Non-basophilic hyaline bodies ("Lewy body-like structures") seen in H&E stained sections,
    - Increased immunocytochemical staining for phosphorylated neurofiliments in perikarya of the motor neurons;
    - Atrophy or loss of the arborizations of the dendrites of the motor neurons of the anterior horns of the spinal cord and brainstem motor nuclei.
    - Wallerian degeneration in the anterior roots.

SAMPLE

Features that are compatible with, and do not exclude, the diagnoses:
Variable involvement of Clark's nucleus and the spinocerebellar tracts; posterior root ganglia, the posterior columns of the spinal cord and peripheral sensory nerves; the brainstem reticular neurons and the anterolateral columns of the spinal cord; the thalamus; subthalamic nucleus; and the subastantia nigra.

Features that rule out the diagnosis or suggest the presence of additional disease:
Major pathological involvement of other parts of the nervous system, including: cerebral cortex other than the motor cortex; basal ganglia; substantia nigra; cerebellum; cranial nerves II and VIII; dorsal root ganglia.

The following cellular pathological changes in the involved neuronal systems described above:

The ALS Association, 27001 Agoura Road, Suite 250, Calabasas Hills, CA 91301-5104,
Phone: (800) 782-4747 / alsinfo@alsa-national.org / www.alsa.org

- Extensive central chromatolysis;
- Extensive active neuronophagia;
- Neurofibrillary tangles;
- The presence of abnormal storage material;
- The presence of significant spongiform change;
- The presence of extensive inflammatory cell infiltration.

Electron-microscopic studies

Features required for the diagnosis:
Ultrastructural studies are not required for the diagnosis of ALS

Features that strongly support the diagnosis:

1) Accumulation of interwoven bundles of 10 nm neurofilaments in axonal spheroids or motor neuron perikarya, and thicker linear structures associated with dense granules (Hirano et al. 1984 J. Neuropath. Ex. Neurol. 43:461),
2) Bunina bodies (Hart et al. 1944 Acta Neuropath. 38:225).

Features that are compatible with, and do not exclude the diagnosis: The presence of intra-axonal polyglucosan bodies.

Features that rule out the diagnosis or suggest the presence of additional disease;

1) The presence of significant numbers of definite viral particles,
2) The presence of significant amounts of abnormal storage materials.
3) Extensive vacuolation of neuronal perikarya.

SAMPLE

fy i... for your information

Glossary

Definite	specific clinical exclusionary criteria met; no other diagnosis possible on basis of clinical distribution or laboratory findings
Dementia	progressive deterioration of specific cognitive functions
Extraphyramidal	clinical features localizable to basal ganglia and/or midline cerebellum
Hyperreflexia	spread of deep tendon reflex outside stimulated territory
Minor	subjective and objective complaints confirmed by examination (utilization of instrumental sensory testing may increase the detection of sensory abnormalities)
Onset	time of first subjective symptom noticed by patient which later is confirmed by examination
Possible	specific clinical and exclusionary criteria met
Probable	specific clinical and exclusionary criteria
Radicular	distribution conforming to particular nerve root
Region	brainstem, cervical, thoracic or lumbosacral spinal cord level (regional involvement is defined by either right or left sided signs
Required	necessary or sufficient
Segment	single brainstem or spinal cord level
Spread	involvement of new anatomic segments or regions in the central nervous system
Support	neither necessary nor sufficient, but may suggest

SAMPLE

The ALS Association, 27001 Agoura Road, Suite 250, Calabasas Hills, CA 91301-5104,
Phone: (800) 782-4747 / alsinfo@alsa-national.org / www.alsa.org

Systemic	non-central nervous system
Weakness	decreased isometric strength
Worsening	increased weakness of muscles in previously affected segment

--Reprinted from the Journal of the Neurological Sciences 124 (suppl.) 1994, Rev 5/96

The ALS Association, 27001 Agoura Road, Suite 250, Calabasas Hills, CA 91301-5104,
Phone: (800) 782-4747 / alsinfo@alsa-national.org / www.alsa.org

SAMPLE

APPENDIX 10 – SNIFF NASAL INSPIRATORY PRESSURE (SNIP) & FORCED VITAL CAPACITY (FVC)

Normal Sniff Nasal Inspiratory Pressure (SNIP)

**Age groups (years)**	**N (number of subjects)**	**SNIP cm H_2O (standard deviation)**
**Men**		
20 to 35	20	117 (29.5)
36 to 50	20	105 (24.5)
51 to 65	20	111.5 (15.5)
66 to 80	20	91 (21.5)
**Women**		
20 to 35	20	84 (14.5)
36 to 50	20	94 (21)
51 to 65	20	83.5 (18)
66 to 80	20	75.5 (11)

Table representing maximum values of SNIP in healthy adults.

Data from: Uldrey C, Fitting JW. Maximal values of sniff nasal inspiratory pressure in healthy subjects. Thorax 1995; 50:371.

Graphic 102592 Version 1.0

Forced Vital Capacity (FVC) – Ventilatory support is indicated when the FVC is less than 50 per cent of predicted in many patients with respiratory muscle weakness[57] therefore an FVC ≥50% of predicted value for gender, height and age is required for trial entry. FVC will also be measured at Week 12 and Week 24 as detailed in the Schedule of Assessments.

SAMPLE

APPENDIX 11 – TARGETED METABOLIC MARKERS IN SERUM (AND URINE P75)

Study Rationale

ALS, like all neurodegenerative conditions, leads to progressive neuronal loss. This loss, or damage, to nervous tissues is accompanied by the leaching into the blood and urine of various proteins and metabolites related to the breakdown of the tissue. Many of these breakdown products picked up in the blood will be common across the different neurodegenerative aetiologies. Some biomarkers are well established as indicators of brain damage in conditions including traumatic brain injury, stroke and ALS (such as lactic acid, $p75^{ECD}$ and NAA), whilst others are more novel and their utility is only now being uncovered. Such biomarker profiles will be of great future importance both in monitoring patient progress and also their response to treatments such as ILB. Here a panel of biomarkers have been chosen that are relevant to the known activities of ILB and to ALS, so they will provide insight into the mechanism and effects of ILB in the patient population under study.

Study Design

Biomarker score as a cumulative index linked to disease severity

We recommend that the study design includes a group of control, age and sex matched, healthy controls, to perform the comparison of the different compounds determined in ALS patients. It should be underlined that a control group is mandatory to calculate the Biomarker Score. In fact, this cumulative index of pathological state needs: i) to determine what compounds are statistically different in controls and patients at zero time, in order to evidence what are the potential biomarkers of the disease; ii) to calculate the 10 – 90 percentiles of each biomarker in each control and patient at any time point; iii) to assign a value of 0 to those biomarkers falling within the 10 – 90 percentiles of controls and a value of 1 to those biomarkers falling outside the 10 – 90 percentiles of controls; iv) to calculate the Biomarker Score by adding all the positive values in each control and patient at any time point; v) to perform all the further statistical comparisons, including the distribution frequency and the posterior probability curves (for a better comprehension of the Biomarker Score see Lazzarino G, Amorini AM, Petzold A, Gasperini C, Ruggieri S, Quartuccio ME, Lazzarino G, Di Stasio E, Tavazzi B. Serum compounds of energy metabolism impairment are related to disability, disease course and neuroimaging in multiple sclerosis. Mol Neurobiol 2017; 54: 7520-7533).

Please note that we can use archived non-matched controls as our reference values.

SAMPLE

Size of the study and number of samples to be assayed

15 ALS patients, 4 serum withdrawals per individual = 60 samples to be assayed for the serum samples. 4 PBMC samples per individual to be collected and stored for later analysis.

Sample withdrawals, handling, storage and shipping

Serum samples: In the case of serum samples, peripheral venous blood samples should be collected after at least 15 minutes of complete rest, using the standard tourniquet procedure, from the antecubital vein into a single VACUETTE® polypropylene tube containing serum separator and clot activator (Greiner-Bio One GmbH, Kremsmunster, Austria). After 30 minutes at room temperature, blood withdrawals should be centrifuged at 1,890 x g for 10 min and the resulting serum samples should be divided into two aliquots and clearly labeled to identify the sample.

Prior to collection of blood samples please contact either:

Professor Ann Logan on 07891 075968
Or
Dr Valentina Di Pietro on 07982 708 937

To inform of them of an imminent sample to collect from the Clinical Research Facility laboratory.

Please do not freeze the serum samples.

Analyses of metabolites in serum

a) HPLC analysis of purines, pyrimidines, oxidative/nitrosative stress compounds (n = 60)
b) HPLC analysis of fat-soluble vitamins and antioxidants (n = 60)
c) HPLC analysis of amino acids and amino group-containing compounds (n = 60)d) Spectrophotometric analysis of lactate (n = 60)

The 3 sets of HPLC analyses a), b) and c) require separate methods to determine the different classes of metabolites. Hence, 180 separate HPLC analyses + 60 spectrophotometric lactate analyses are required for serum.

PBMC samples: In the case of PBMC sample collection, peripheral venous blood samples should be collected after at least 15 minutes of complete rest, using the standard tourniquet procedure, from the antecubital vein into a single standard EDTA vacutainer (Greiner-Bio One GmbH, Kremsmunster,

SAMPLE

Austria). 10ml of peripheral venous blood should be collected and securely transported to the Neuregenix laboratory.

Prior to collection of blood samples please contact either:

Professor Ann Logan on 07891 075968

Or

Dr Valentina Di Pietro on 07982 708 937

To inform them of an imminent sample to collect from the Clinical Research Facility laboratory.

Please do not centrifuge or freeze the PBMC samples

Analyses of PBMC

a) PBMC samples will be collected for analysis of change in phenotypic balance.

Urine samples: In the case of urine, if possible the first urine of the morning should be obtained from each patient. Samples (at least 500µl, five hundred microliters) should be collected and clearly labelled to identify the sample

Please contact either:

Professor Ann Logan on 07891 075968

Or

Dr Valentina Di Pietro on 07982 708 937

To inform them of an imminent sample to collect from the Clinical Research Facility laboratory.

Please do not freeze the urine samples.

Analyses of p75^{ECD} in urine

a) ELISA of p75^{ECD} (n=60)

ALS Trial Office

D^{3}B – Diagnostics Drugs Devices & Biomarkers, CRCTU

SAMPLE

Heritage Building
5th Floor East, ITM,
Mindelsohn Way,
Edgbaston,
Birmingham,
B15 2TH

Enquires
☎ +44(0) 121 371 8108 🖶 + 44(0) 121 371 7246
✉ ALS@trials.bham.ac.uk

SAMPLE
