# Supplementary material for: A low molecular weight dextran sulphate, ILB®, for the treatment of amyotrophic lateral sclerosis (ALS): An open-label, single-arm, single-centre, phase II trial
Source: PLoS One. 2024 Jul 11;19(7):e0291285. doi: 10.1371/journal.pone.0291285 (PMC11239073; doi:10.1371/journal.pone.0291285)
Supplement: S2 Appendix — A sample version of the finalised trial patient information sheet (Version 8.0) dated 04-Mar-2020. (PDF) [file pone.0291285.s002.pdf]

Supplementary appendix 2: Sample version of the ALS trial patient information sheet

To be printed on hospital headed paper

Patient Information Sheet

SAMPLE

ALS Study

A Phase II pilot safety and tolerability study of ILB in patients with Motor Neurone Disease (MND)/ Amyotrophic Lateral Sclerosis (ALS)

Dear Patient,

We would like to invite you to take part in a research study – The Amyotrophic Lateral Sclerosis (ALS) Study which is sponsored by the University of Birmingham (the Sponsor) and is being carried out as a collaboration between the University and a Swedish industry company called TikoMed. Joining the study is entirely up to you, before you decide we would like you to understand why the research is being done and what it would involve for you. One of our team will go through this information sheet with you to help you decide if you would like to take part and answer any questions you may have. Please feel free to talk to others about the study if you wish.

Part 1 tells you the purpose of this study and what will happen to you if you take part.
Part 2 gives you more detailed information about the conduct of the study.

Please ask us if there is anything that is not clear

Original to be kept in the Investigator Site File, 1 copy in hospital notes, 1 copy to the patient,*1 copy to the ALS Trial Office*

Part 1

1. Purpose and participation

What is the purpose of this Clinical Study?

We are doing this study to look at the safety and acceptability of a new drug called ILB, for patients with Amyotrophic Lateral Sclerosis (ALS). We also hope to gain a better understanding of the effects of this drug on the symptoms of ALS, to assess if the experimental drug improves symptoms and the effect of this drug on the quality of life of patients living with the disease. A study in ALS is also being run in parallel to this UK study using the same drug (ILB). The Swedish study aims to recruit 15 patients, treating with ILB over a 4-week period.

We are asking if you would like to take part in a clinical study in the UK. We invite you to receive a weekly injection called a subcutaneous injection, which is a method of giving a medication in the fatty layer of tissue just under the skin. Subcutaneous injections can be given in a number of sites, including the arms, legs, abdomen and buttocks.

It is possible you will require several injections at each treatment visit, (given in different sites), to ensure we are able to give you the correct dose based on your body weight.

In addition to the weekly study injections, you will continue to receive your usual standard of care from your doctors, whether you take part in the study or not.

Why have I been invited?

You have been diagnosed with ALS and we believe that you may be eligible to take part in the study.

Do I have to take part?

SAMPLE

No. Your decision to take part in this study is entirely voluntary. You do not have to give a reason if you decide not to enter the study and your subsequent treatment will not be altered or affected in any way. If you choose to take part in the study but later choose to withdraw, we would still like to collect information about your treatment, as this will be invaluable to our research. If you have any objection to this, please let your doctor know. Data already collected prior to withdrawal will be kept and analysed as part of the study as planned.

If you decide not to take part, your doctor will continue to treat you with the best means available and the standard of your care will not be affected in any way.

<u>What will happen if I don't want to carry on with the study?</u>

You are free to withdraw from the study at any time and do not have to give a reason for this. Your doctor will continue to treat you with the best means available and the standard of your care will not be affected. At this point, your care will return to your normal standard of care clinic with the specialist doctor managing your ALS disease.

If you choose to take part in the study but later withdraw, we would still like to collect information about your treatment, as this will be valuable to our research. If you have any objection to this please let your doctor know at the time you decide to withdraw from the study. Data already collected prior to withdrawal will be kept and analysed as part of the study as planned.

SAMPLE

2. What is the drug being tested?

<u>ILB (the study drug)</u>

ILB is a type of low molecular weight dextran sulfate.

TikoMed initially developed ILB by investigating its use in diabetic patients, although the medication was originally developed and explored more than 50 years ago for its blood thinning abilities. TikoMed along with colleagues at the University of Birmingham have tested this drug in a number of pre-clinical

(animal studies) and in 79 humans. Results of these studies indicate there may be some beneficial effects in using ILB in a number of diseases including ALS. Early studies in healthy volunteers and in rats indicate the study drug, ILB, may have some role to play in the mechanism that helps to protect the motor neurons (nerve cells) which are the specific nerve cells affected in people with ALS disease. In the studies using ILB in healthy volunteers, the study drug was found to increase the levels (in the blood) of a growth factor called Hepatocyte Growth Factor (HGF) and it is this increase in HGF that may help to protect the nerve cells.

This study and the study being carried out in Sweden will be the first studies using ILB in this patient group.

Has the drug been tested before?

ILB has been shown to have some potential benefits in a number of conditions during animal testing.

Since its development, five studies have been carried out; four studies in healthy volunteers (69 participants) and one study in diabetic patients (10 patients), 79 participants in total have received this medication to date. In the studies, the time it took for the blood to clot was very slightly longer than before taking the drug, which may lead to bleeding complications. Two participants experienced a mild and transient allergic reaction (mild pain, itching and redness on the skin), the effects had disappeared within 30 minutes to 4 hours after administration of the drug.

This study is to investigate the safety and acceptability of ILB in ALS patients.

What happens if I don't take part in this study?

If you decide not to take part in this study, you will continue to receive the best standard of care. The specialist Doctor managing your ALS disease will continue to manage your disease. Your standard of care will not be affected in any way should you decide not to take part in this study.

This completes Part 1 of the Patient Information Sheet.

SAMPLE

If the information in Part 1 has interested you and you are considering participation, please read the additional information in Part 2 before making any decision.

Part 2

1. What does taking part involve?

Consent, Screening and Treatment

We will describe the study and talk through this information sheet with you. You will be given time to decide whether you wish to participate in the study. During this time, you can discuss this with anyone you wish including your GP.

If you decide to take part, you will be asked to sign a consent form and will be given a copy to keep. Following this, you will initially be asked to attend 15 hospital appointments over 6 months. The study will take 30 months from start to finish, although your individual contribution will be for just 6 months. There will be a chance to extend your treatment for up to a maximum of 48 weeks upon review with your study doctor. This is discussed below.

Screening Visit (Visit 1 - within 14 days of starting study drug or within 28 days of starting study drug for those on Riluzole prior to study entry)

Patients taking Riluzole prior to study entry will have their Riluzole medication withdrawn to take part in the study and put on the study drug instead. You will discontinue Riluzole on the day of consent to the study and would not receive the first injection of the study drug until at least 28 days have elapsed due to the possible interaction between Riluzole and the new drug (ILB).

At the screening visit, you will be asked to sign a consent form to indicate you agree to take part in the study. We will then check your eligibility by asking relevant questions and performing tests. Some of the tests you will have already had to confirm your ALS diagnosis prior to you being approached about the study. During this visit, we will ask you to complete a questionnaire about your health and your ability to carry out activities of daily living. We will ask you to complete this same questionnaire each

SAMPLE

time you attend clinic for treatment and during follow up appointments after completing the treatment to see if there are any changes in your quality of life. Your screening visit can take up to half a day to complete.

Week 1, Visit 2 (Day 1) - Start Study Drug - Using the test results from Visit 1 and additional tests performed during this visit your eligibility to go into the study will be confirmed. At this visit, one of two things will happen, either your eligibility for the study will be confirmed and you will be registered into the study and will receive your first study injections by a trained specialist nurse. Or you will be withdrawn from the study due to you not meeting the eligibility criteria and your care will return to your normal standard of care clinic with the specialist doctor managing your ALS disease.

If you are eligible and registered into the study, blood tests will be performed prior to the study injection and at 30, 60 (1 hour), 120 minutes (2 hours), 150, 180, 240 and 360 minutes after your injections, these are called Pharmacokinetics or PKs. This visit can take up to half a day to complete.

Weeks 2, 3 and 4 (Visits 3, 4 and 5) – Treatment These visits are called treatment visits. During this visit you will be assessed by the Doctor looking after your care and see a number of specialist nurses. We will perform tests to check your general health, and to confirm you are well, and that the drug is not having a detrimental effect on your health, and it is therefore safe for you to continue to receive the study drug. Tests include; Quality of life questionnaires, blood tests. A trained specialist nurse will give you the study injections in clinic.

Week 5, Visit 6 - This visit is also a treatment visit, but a slightly longer visit to allow time for some extra tests. Again, during the visit you will be assessed by the Doctor looking after your care and see a number of specialist nurses. We will perform tests to check your general health, and to confirm you are well, and that the drug is not having a detrimental effect on your health, and it is therefore safe for you to continue to receive the study drug. Tests include; Quality of life questionnaires, an electrocardiogram (ECG), lung function tests, blood tests and urine samples. A trained specialist nurse will give you the study injections in clinic.
Blood tests will be performed prior to the study injection. This visit can take up to half a day to complete.

Weeks 6, 7, 8 and 9 (Visits 7, 8, 9 and 10) - Treatment These visits are also treatment visits. During this visit you will be assessed by the Doctor looking after your care and see a number of specialist nurses. We will perform tests to check your general health, and to confirm you are well, and that the drug is not having a detrimental effect on your health, and it is therefore safe for you to continue to receive the study drug. Tests include; Quality of life questionnaires, blood tests. A trained specialist nurse will give you the study injections in clinic.

Treatment Continuation

At week 10, you will discuss with the study doctor the option of continuing treatment. If you are eligible and you and the study doctor agree it is the best treatment option for you, you will be asked to sign a Treatment Extension consent form and follow a different schedule of visits (outlined in the Patient Information Sheet- Treatment Extension). Treatment extension can extend up to a maximum of 48 weeks as per discussion with the study doctor.

The following visits are if you are ineligible or decline the treatment extension:

Week 12 (Visit 13) - End of Treatment/Follow-up visit - This visit is designed to check your general health and to do tests (including breathing tests, blood and urine tests). We will also ask you to complete the questionnaire about your health and your ability to carry out activities of daily living to see if there are any changes in your quality of life. This visit can take up to half a day to complete.

Week 16 (Visit 14) – Follow- up visit - These visits are designed to check your general health and to do tests including blood tests and we will ask you to complete a questionnaire about your health and your ability to carry out activities of daily living to see if there are any changes in your quality of life. This visit can take up to 2 hours to complete.

Week 20 (Visit 15) These visits are designed to check your general health and to do tests including blood tests and we will ask you to complete a questionnaire about your health and your ability to carry out activities of daily living to see if there are any changes in your quality of life. This visit can take up to 2 hours to complete.

Week 24 (Visit 16) These visits are designed to check your general health and to do tests (including breathing tests, blood and urine tests). We will ask you to complete a questionnaire about your health

SAMPLE

and your ability to carry out activities of daily living to see if there are any changes in your quality of life. This visit can take up to half a day to complete. This will be your last study visit.

Note: It is possible that you will be asked to attend additional unscheduled visits beyond those described if we feel that you need to be seen by a Doctor. Additional visits will also be necessary if you decide to extend treatment.

SAMPLE

Schedule of Assessments (1) Main Trial

	**Screening**<br>≤ 14 days of Registration<br>(up to 28 days prior to Day 1 for those patients taking Riluzole prior to trial entry)	**Treatment Period**										**Follow-up (Observational Period) following discontinuation of ILB**<br>All Follow-up visits (+/- 3 days)			
		**Week 1**	**Week 2**	**Week 3**	**Week 4**	**Week 5**	**Week 6**	**Week 7**	**Week 8**	**Week 9**	**Week 10**	**Week 12**	**Week 16**	**Week 20**	**Week 24**
Time schedule<br>**Days +/- 1 day**	**Visit 1**<br>**Day ≤ 14**	**Visit 2**<br>**Day 1**	**Visit 3**<br>**Day 8**	**Visit 4**<br>**Day 15**	**Visit 5**<br>**Day 22**	**Visit 6**<br>**Day 29**	**Visit 7**<br>**Day 36**	**Visit 8**<br>**Day 43**	**Visit 9**<br>**Day 50**	**Visit 10**<br>**Day 57**	**Visit 11**<br>**Day 64**	**Visit 13**<br>**Day 78**	**Visit 14**<br>**Day 106**	**Visit 15**<br>**Day 134**	**Visit 16**<br>**Day 162**
1. Medical history	X														
2. Eligibility Assessment	X	X													
3. ALSFRS-R	X	X	X	X	X	X	X	X	X	X	X	X	X	X	X
4. Informed Consent	X														
5. Registration		X													
6. Concomitant drug history	X	X	X	X	X	X	X	X	X	X	X	X	X	X	X
7. Vital signs	X	X	X	X	X	X	X	X	X	X	X	X	X	X	X
8. ECG	X					X					X	X			
9. Lung Function Tests (FVC)	X					X				X		X			X
10. Sniff Nasal Inspiratory Pressure	X					X				X		X			X

Original to be kept in the Investigator Site File, 1 copy in hospital notes, 1 copy to the patient,*1 copy to the ALS Trial Office*

	**Screening**<br>≤ 14 days of Registration<br>(up to 28 days prior to Day 1 for those patients taking Riluzole prior to trial entry)	**Treatment Period**										**Follow-up (Observational Period) following discontinuation of ILB**<br>All Follow-up visits (+/- 3 days)			
		**Week 1**	**Week 2**	**Week 3**	**Week 4**	**Week 5**	**Week 6**	**Week 7**	**Week 8**	**Week 9**	**Week 10**	**Week 12**	**Week 16**	**Week 20**	**Week 24**
Time schedule<br>**Days +/- 1 day**	**Visit 1**<br>**Day ≤ 14**	**Visit 2**<br>**Day 1**	**Visit 3**<br>**Day 8**	**Visit 4**<br>**Day 15**	**Visit 5**<br>**Day 22**	**Visit 6**<br>**Day 29**	**Visit 7**<br>**Day 36**	**Visit 8**<br>**Day 43**	**Visit 9**<br>**Day 50**	**Visit 10**<br>**Day 57**	**Visit 11**<br>**Day 64**	**Visit 13**<br>**Day 78**	**Visit 14**<br>**Day 106**	**Visit 15**<br>**Day 134**	**Visit 16**<br>**Day 162**
(SNIP)															
11.Haematology Tests	X	X	X	X	X	X	X	X	X	X	X	X	X	X	X
12. Coagulation Tests	X	X	X	X	X	X	X	X	X	X	X	X	X	X	X
13 Biochemistry Tests	X	X	X	X	X	X	X	X	X	X	X	X	X	X	X
14. Creatine Kinase (serum)	X					X					X	X			
15.U&Es & LFTs	X	X	X	X	X	X	X	X	X	X	X	X	X	X	X
16.Thyroid Function Tests	X										X	X			X
17.Immunoglobulins	X										X	X			
18.Quality of Life Questionnaire	X	X	X	X	X	X	X	X	X	X	X	X	X	X	X
19. Urinary p75[ECD12]		X				X					X	X			X
20. NfL levels		X				X					X	X			

Original to be kept in the Investigator Site File, 1 copy in hospital notes, 1 copy to the patient,*1 copy to the ALS Trial Office*

	Screening<br>≤ 14 days of Registration<br>(up to 28 days prior to Day 1 for those patients taking Riluzole prior to trial entry)	**Treatment Period**										**Follow-up (Observational Period) following discontinuation of ILB**<br>All Follow-up visits (+/- 3 days)			
		**Week 1**	**Week 2**	**Week 3**	**Week 4**	**Week 5**	**Week 6**	**Week 7**	**Week 8**	**Week 9**	**Week 10**	**Week 12**	**Week 16**	**Week 20**	**Week 24**
Time schedule<br>**Days +/- 1 day**	**Visit 1**<br>**Day ≤ 14**	**Visit 2**<br>**Day 1**	**Visit 3**<br>**Day 8**	**Visit 4**<br>**Day 15**	**Visit 5**<br>**Day 22**	**Visit 6**<br>**Day 29**	**Visit 7**<br>**Day 36**	**Visit 8**<br>**Day 43**	**Visit 9**<br>**Day 50**	**Visit 10**<br>**Day 57**	**Visit 11**<br>**Day 64**	**Visit 13**<br>**Day 78**	**Visit 14**<br>**Day 106**	**Visit 15**<br>**Day 134**	**Visit 16**<br>**Day 162**
21. Exploratory Biomarkers (serum and PBMC)		X				X					X	X			X
22. Pregnancy test (for WOCBP only)	X	X			X				X		X	X			
23.PK Sampling		X													
24. Dynamometer test	X	X	X	X	X	X	X	X	X	X	X				
25. Study drug administration		X	X	X	X	X	X	X	X	X	X				
26. Adverse Event/Toxicity Reporting		Throughout						Throughout				Throughout			

Schedule of Assessments (2) Treatment Extension (Week 11 to Week 24)

**Schedule of Assessments**	**Week 11**	**Week 12**	**Week 13**	**Week 14**	**Week 15**	**Week 16**	**Week 17**	**Week 18**	**Week 19**	**Week 20**	**Week 21**	**Week 22**	**Week 23**	**Week 24**	**Week 26**	**Treatment continuation**
Time schedule **Days +/- 1 day**	**Day 71**	**Day 78**	**Day 85**	**Day 92**	**Day 99**	**Day 106**	**Day 113**	**Day 120**	**Day 127**	**Day 134**	**Day 141**	**Day 148**	**Day 155**	**Day 162**	**End of treatment visit Only for those patients completing up to 48 weeks of treatment**	**Treatment beyond 24 weeks will be a discussion between patient and clinician. If treatment is continued, treatment and visits will follow the same pattern as week 11-24 for a maximum of 48 weeks**
1. Re-consent	X															
2. ALSFRS-R	X			X					X					X	X	
3. Concomitant drug history	Throughout															
4. Vital signs				X					X					X	X	
5. ECG				X					X					X	X	
6. Lung Function Tests (FVC)				X					X					X	X	
7. Sniff Nasal Inspiratory Pressure (SNIP)				X					X					X	X	
8.Haematology Tests				X					X					X	X	
9. Coagulation Tests				X					X					X	X	
10 Biochemistry Tests				X					X					X	X	
11. Creatinine Kinase (serum)				X					X					X	X	
12.U&Es & LFTs				X					X					X	X	

Original to be kept in the Investigator Site File, 1 copy in hospital notes, 1 copy to the patient,*1 copy to the ALS Trial Office*

**Schedule of Assessments**	**Week 11**	**Week 12**	**Week 13**	**Week 14**	**Week 15**	**Week 16**	**Week 17**	**Week 18**	**Week 19**	**Week 20**	**Week 21**	**Week 22**	**Week 23**	**Week 24**	**Week 26**	**Treatment continuation**
Time schedule **Days +/- 1 day**	**Day 71**	**Day 78**	**Day 85**	**Day 92**	**Day 99**	**Day 106**	**Day 113**	**Day 120**	**Day 127**	**Day 134**	**Day 141**	**Day 148**	**Day 155**	**Day 162**	**End of treatment visit Only for those patients completing up to 48 weeks of treatment**	**Treatment beyond 24 weeks will be a discussion between patient and clinician. If treatment is continued, treatment and visits will follow the same pattern as week 11-24 for a maximum of 48 weeks**
13.Thyroid Function Tests				X					X					X	X	
14.Immunoglobulins				X					X					X	X	
15.Quality of Life Questionnaire				X					X					X	X	
16 Urinary p75^{ECD12}														X		
17. NfL levels														X	X	
18. Exploratory Biomarkers (serum and PBMC)														X	X	
19. Pregnancy test (for WOCBP only)				X					X					X	X	
20. Dynamometer test				X					X					X	X	
21. Study drug administration	X	X	X	X	X	X	X	X	X	X	X	X	X	X		
22. Adverse Event/Toxicity Reporting	Throughout															

Original to be kept in the Investigator Site File, 1 copy in hospital notes, 1 copy to the patient,*1 copy to the ALS Trial Office*

Schedule of Assessments (2) Treatment Extension (Week 25 to Week 38)

Schedule of Assessments	Week 25	Week 26	Week 27	Week 28	Week 29	Week 30	Week 31	Week 32	Week 33	Week 34	Week 35	Week 36	Week 37	Week 38	Week 40	Treatment continuation
Time schedule **Days +/- 1 day**	**Day 169**	**Day 176**	**Day 183**	**Day 190**	**Day 197**	**Day 204**	**Day 211**	**Day 218**	**Day 225**	**Day 232**	**Day 239**	**Day 246**	**Day 253**	**Day 260**	**End of treatment visit** **Only for those patients completing up to 38 weeks of treatment**	**Extended treatment will be a discussion between patient and clinician. If treatment is continued, treatment and visits will follow the same pattern as week 11-24 for a maximum of 48 weeks**
1. Re-consent																
2. ALSFRS-R				X					X					X	X	
3. Concomitant drug history	Throughout															
4. Vital signs				X					X					X	X	
5. ECG				X					X					X	X	
6. Lung Function Tests (FVC)				X					X					X	X	
7. Sniff Nasal Inspiratory Pressure (SNIP)				X					X					X	X	
8.Haematology Tests				X					X					X	X	
9. Coagulation Tests				X					X					X	X	
10 Biochemistry Tests				X					X					X	X	
11. Creatinine Kinase (serum)				X					X					X	X	

Original to be kept in the Investigator Site File, 1 copy in hospital notes, 1 copy to the patient,*1 copy to the ALS Trial Office*

**Schedule of Assessments**	**Week 25**	**Week 26**	**Week 27**	**Week 28**	**Week 29**	**Week 30**	**Week 31**	**Week 32**	**Week 33**	**Week 34**	**Week 35**	**Week 36**	**Week 37**	**Week 38**	**Week 40**	**Treatment continuation**
Time schedule **Days +/- 1 day**	**Day 169**	**Day 176**	**Day 183**	**Day 190**	**Day 197**	**Day 204**	**Day 211**	**Day 218**	**Day 225**	**Day 232**	**Day 239**	**Day 246**	**Day 253**	**Day 260**	**End of treatment visit** **Only for those patients completing up to 38 weeks of treatment**	**Extended treatment will be a discussion between patient and clinician. If treatment is continued, treatment and visits will follow the same pattern as week 11-24 for a maximum of 48 weeks**
12.U&Es & LFTs				X					X					X	X	
13.Thyroid Function Tests																
14.Immunoglobulins				X					X					X	X	
15.Quality of Life Questionnaire				X					X					X	X	
16 Urinary $p75^{ECD12}$														X		
17. NfL levels														X	X	
18. Exploratory Biomarkers (serum and PBMC)														X	X	
19. Pregnancy test (for WOCBP only)				X					X					X	X	
20. Dynamometer test				X					X					X	X	

Original to be kept in the Investigator Site File, 1 copy in hospital notes, 1 copy to the patient,*1 copy to the ALS Trial Office*

**Schedule of Assessments**	**Week 25**	**Week 26**	**Week 27**	**Week 28**	**Week 29**	**Week 30**	**Week 31**	**Week 32**	**Week 33**	**Week 34**	**Week 35**	**Week 36**	**Week 37**	**Week 38**	**Week 40**	**Treatment continuation**
Time schedule **Days +/- 1 day**	**Day 169**	**Day 176**	**Day 183**	**Day 190**	**Day 197**	**Day 204**	**Day 211**	**Day 218**	**Day 225**	**Day 232**	**Day 239**	**Day 246**	**Day 253**	**Day 260**	**End of treatment visit Only for those patients completing up to 38 weeks of treatment**	**Extended treatment will be a discussion between patient and clinician. If treatment is continued, treatment and visits will follow the same pattern as week 11-24 for a maximum of 48 weeks**
21. Study drug administration	X	X	X	X	X	X	X	X	X	X	X	X	X	X		
22. Adverse Event/Toxicity Reporting	Throughout															

Schedule of Assessments (2) Treatment Extension (Week 39 to Week 48)

**Schedule of Assessments**	**Week 39**	**Week 40**	**Week 41**	**Week 42**	**Week 43**	**Week 44**	**Week 45**	**Week 46**	**Week 47**	**Week 48**	**Week 50**
Time schedule **Days +/- 1 day**	**Day 267**	**Day 274**	**Day 281**	**Day 288**	**Day 295**	**Day 302**	**Day 309**	**Day 316**	**Day 323**	**Day 330**	**End of treatment visit** **Only for those patients completing up to 48 weeks of treatment**
1. Re-consent											
2. ALSFRS-R					X					X	X
3. Concomitant drug history	Throughout										
4. Vital signs					X					X	X
5. ECG					X					X	X
6. Lung Function Tests (FVC)					X					X	X
7. Sniff Nasal Inspiratory Pressure (SNIP)					X					X	X
8.Haematology Tests					X					X	X
9. Coagulation Tests					X					X	X
10 Biochemistry Tests					X					X	X
11. Creatinine Kinase (serum)					X					X	X
12.U&Es & LFTs					X					X	X
13.Thyroid Function Tests											

Original to be kept in the Investigator Site File, 1 copy in hospital notes, 1 copy to the patient,*1 copy to the ALS Trial Office*

**Schedule of Assessments**	**Week 39**	**Week 40**	**Week 41**	**Week 42**	**Week 43**	**Week 44**	**Week 45**	**Week 46**	**Week 47**	**Week 48**	**Week 50**
Time schedule **Days +/- 1 day**	**Day 267**	**Day 274**	**Day 281**	**Day 288**	**Day 295**	**Day 302**	**Day 309**	**Day 316**	**Day 323**	**Day 330**	**End of treatment visit** **Only for those patients completing up to 48 weeks of treatment**
14.Immunoglobulins					X					X	X
15.Quality of Life Questionnaire					X					X	X
16 Urinary $p75^{ECD12}$										X	
17. NfL levels										X	X
18. Exploratory Biomarkers (serum and PBMC)										X	X
19. Pregnancy test (for WOCBP only)					X					X	X
20. Dynamometer test					X					X	X
21. Study drug administration	X	X	X	X	X	X	X	X	X	X	
22. Adverse Event/Toxicity Reporting	Throughout										

Original to be kept in the Investigator Site File, 1 copy in hospital notes, 1 copy to the patient,*1 copy to the ALS Trial Office*

Assessments

Medical History & Eligibility Assessment: an assessment of your medical history, and screening tests (performed prior to the study to confirm your ALS diagnosis)

The Amyotrophic Lateral Sclerosis Functional Rating Scale (Revised) (ALSFRS-R): A functional rating scale or questionnaire to assess your ability to perform activities of daily living

Concomitant drug history: a history of all medications you are using prior to and during the study

Vital signs: recordings of your Blood pressure, heart rate, temperature, oxygen levels, body weight and height (height measurements at the screening visit only)

Electrocardiogram (ECG): This test allows the study doctor to see the electrical activity of your heart. Small sticky patches called electrodes are attached to your arms, legs and chest. These detect the electrical signals that make your heart beat, and transmit the signals via wires to an ECG recording machine, which in turn transcribes the signals on to paper, which a clinician (doctor or trained nurse) can interpret.

Lung Function Tests (FVC): this is a breathing test

Sniff Nasal Inspiratory Pressure (SNIP): this is a breathing test

Haematology Tests (also known as a Full Blood Count): This is a routine test that provides information on the various components of blood, including red and white blood cells and platelets. Blood samples will be taken at every visit. At the screening visit, these tests will be used to see if you are eligible for the study and to provide baseline values by which to compare any subsequent change after treatment. After treatment, the purpose of the blood tests is to monitor your wellbeing and health, and determine what effects the drug has had.

Coagulation Tests: These are a group of tests designed to detect possible problems with blood coagulation/clotting mechanisms, and can indicate if a you have a tendency to bleed. These blood tests will also be taken at every visit. At the screening visit, they will be used to see if you are eligible for the study and to provide baseline values by which to compare any subsequent change after

SAMPLE

treatment. We will also monitor the time it takes for your blood to clot. After treatment, the purpose of the blood tests is to monitor your wellbeing and health, and determine what effects the drug has had.

Biochemistry, Creatinine Kinase, Urea and Electrolytes (U&Es), Liver Function Tests (LFTs), thyroid function tests, Immunoglobulins: These tests will tell us about your liver, kidneys, thyroid and immune system. These blood tests will also be taken at each visit. At the screening visit, they will be used to see if you are eligible for the study and to provide baseline values by which to compare any subsequent change after treatment, including effects on kidney and liver functions. After treatment, the purpose of the blood tests is to monitor your wellbeing and health, and determine what effects the drug has had.

*Quality of Life Questionnaire (**ALSAQ-40**):* We will ask you to complete this questionnaire at every visit to help us assess your ALS status (or disease) and monitor any changes both during (treatment visits) and after treatment (at follow-up visits)

Urinary p75^{ECD}: this is a urine test. The purpose of this test is to help us assess the effects of the drug on your ALS disease

Neurofilament Light Chain (NfL) sample levels: This blood test will measure the levels of a particular substance in the blood. This blood test may help us to assess the effects of the drug on your ALS disease. 6 ml (equivalent to 1 teaspoon) of blood will be collected at each visit specified in the Schedule of Assessments.

Exploratory Biomarkers: Components of the blood sample (serum and peripheral blood mononuclear cells (PBMC)) will be stored for future analysis looking for markers of the condition.

Pregnancy test: this urine test will be performed on all women of child-bearing potential (WOCBP) who have not had a hysterectomy

PK Sampling (pharmacokinetic tests): These blood tests will provide us with information on how the body handles the study drug. These are blood tests taken before the study injections and after the injections after 30 minutes, 60 minutes (1 hour), 120 minutes (2 hours), 150 minutes, 180 minutes, 240 minutes and 360 minutes. These tests will only be performed after the first injection at Visit 2

SAMPLE

(Day 1) and will help us to understand what your body does to the drug and to understand what the drug does to you. The total volume of blood collected for pharmacokinetic (PK) analysis will be 38.5 ml (equivalent to 2 and ¾ tablespoons).

Dynamometer test (hand grip test): this test will measure your handgrip strength using a handheld dynamometer. These tests will be carried out weekly for the first 10 weeks, then every 4 weeks if treatment is extended.

Adverse event/Toxicity reporting: An assessment of your current wellbeing and health and an assessment of any symptoms you have been experiencing.

The method of blood samples taken during this trial are the same as that for any other normal blood sample. All blood tests may cause a momentary sharp pain on insertion of the needle and carry a risk of infection, which is extremely rare.

2. Risks and Benefits

What are the potential benefits?

There may be no immediate clinical benefit from taking part , and you may or may not feel better from participating in this study. At best we would hope to reduce the rate of disease progression but unfortunately this drug will not offer a cure for the disease. However, the study has been designed to help us gain a better understanding of this drug and may result in changes in the future treatment and follow-up of patients with ALS.

What are the possible disadvantages and risks of taking part in the study?

The possible disadvantages and risks of taking part in this study are:

- Possible side-effects from taking the drug
- The drug may not be effective and delay you starting standard treatment
- Attending multiple treatment and follow-up appointments

SAMPLE

What are the possible side effects of the drug?

In clinical studies using this treatment in healthy volunteers, the time it took for the blood to clot was very slightly longer than before taking the treatment. We will monitor you closely throughout the study by taking blood samples at every visit to check your clotting ability. If at any time, your results indicate it is not safe for you to receive treatment we will stop treatment immediately and your study doctor will continue to check your blood levels until your results are back to normal for you.

When using this study drug in previous studies in humans, the time it took for the blood to clot was very slightly longer than before taking the drug, and this may lead to bleeding complications. Of note, no spontaneous bleedings were observed in these previous studies. If however, you experience any unusual bleeding, e.g nose bleeds, blood in the urine or coughing up blood, you must inform your study doctor by using the telephone contact number at the end of this information sheet.

Previous studies of the study drug have shown possible side effects from the injection itself, including mild swelling, pain and irritation/itching around the injection sites.

If something goes wrong while you are taking part in this study, we will do everything we can to look after you. We will make sure you are closely supervised while taking part in the study.

You are free to stop at any time without a reason.

If you decide to stop taking part in the study, you will only need to inform your doctor and he or she will withdraw you from the study. This will not affect your standard of care.

Are there any prohibited medications?

At the start of the study, the clinical team will ask you about all of the medicines you are currently taking to make sure you are safe to be considered for the study treatment.

Whilst taking part in the study we will continue to ask about any medications you are taking. You will be asked to let your doctors know if you are taking any other medicines including over the counter medicines, supplements, vitamins or herbal remedies during your time in the study.

SAMPLE

Harm to the unborn child and pregnancy

It is required that both men and women taking part in this study use effective contraception whilst receiving study treatment from the screening visit through to study completion up until the last dose of study drug. For the purposes of participating in this study, effective contraception includes: hormonal contraception, (oral, injection or implant), an intrauterine device (IUD), a vasectomised partner or sexual abstinence. Although unlikely, it is not known whether the study treatment causes harm during pregnancy.

If you are female and of childbearing potential, we will ask you to have a pregnancy test at your clinic visit before every trial treatment to confirm you are not pregnant before administering the treatment injections.

If you are female and become pregnant whilst taking part in the study, or you are male and your partner becomes pregnant, you must tell your study doctor immediately. The pregnancy will need to be monitored and, with the mother`s consent, information about the outcome of the pregnancy will be collected from the mother`s and baby`s medical notes.

Expenses and payments

When you come into hospital for your outpatient study visits, reasonable travel expenses can be reimbursed for these visits where you are able to provide appropriate receipts. Travel by car will be reimbursed at the standard NHS patient reimbursement rate of your local NHS trust (typically 23 pence per mile).

Depending on individual circumstances, it may be possible to find additional ways to help with extra travel or accommodation costs for example due to the distance you have to travel, car parking charges or any other reason. Please discuss this with the local hospital team.

3. Stopping the trial treatment

What happens when the study stops?

SAMPLE

As an experimental drug is being used you will not be able to receive the drug after the trial is complete.

Your subsequent treatment will proceed according to your individual needs and will not be affected by the study, and when the study stops, and you are no longer receiving the study drug your routine ALS care will continue in the normal way.

You will return to clinical care as normal. Your clinical team will continue to follow up your condition and offer standard treatment as required. You will be informed about all treatments (standard of care or experimental) during your clinic appointment with the doctor looking after your care.

What if relevant new information becomes available?

Sometimes we get new information about the treatment being studied. If this happens, your study doctor will inform you and discuss with you whether you should continue in the study. If you decide not to carry on, your study doctor will arrange for your normal care to continue. If you decide to continue in the study, your doctor may ask you to sign an updated consent form. It is possible that your study doctor will suggest you withdraw from the study. He/she will explain the reasons and arrange for your normal care to continue. If the study is stopped for any other reason, we will tell you and arrange your continuing care.

4. What to do if there is a problem

If you have concerns about any aspect of this study, you should ask to speak with the study doctor who will do their best to answer your questions (see contact number at the end of this information sheet).

Complaints

If you remain unhappy and wish to complain formally, you can do this through your hospital's Patient Advice and Liaison Services (PALS), they can be contacted by:

SAMPLE

(Insert local contact details).

If you are harmed during the study

In the event that something does go wrong and you are harmed during the study, there are no special compensation arrangements. The Cancer Research UK Clinical Trials Unit (CRCTU) at University of Birmingham does not hold insurance against claims for compensation for injury caused by participation in this study, and they cannot offer any indemnity. If you are harmed and this is due to someone's negligence then you may have grounds for legal action for compensation against the University of Birmingham or the NHS Trust but you may have to pay your legal costs. NHS Trusts and Non-Trust Hospitals have a duty of care to patients treated, whether or not the patient is taking part in a study and the normal NHS complaints mechanisms will still be available to you (if appropriate).

5. Confidentiality

Will my taking part be kept confidential?

All information collected about you for this study will be subject to the General Data Protection Regulation and Data Protection Act 2018 for health and care research and will be kept strictly confidential. University of Birmingham is the Sponsor for this study. The University of Birmingham will be using information from your medical records in order to undertake this trial and will act as the data controller for this study. This means that the University of Birmingham are responsible for looking after your information and using it properly. University of Birmingham and the NHS will keep identifiable information about you for at least 25 years after the study has finished, to allow the results of the study to be verified if needed.

All information collected by the Sponsor will be securely stored at the Trials Office at the University of Birmingham on paper and electronically and will only be accessible by authorised personnel. The only people in the University of Birmingham who will have access to information that identifies you will be people who manage the study or audit the data collection process.

SAMPLE

The NHS will use your name and contact details to contact you about the research study, and make sure that relevant information about the study is recorded for your care, and to oversee the quality of the trial. With your permission, your research doctor will notify your GP that you intend to participate in the study. They will also send a copy of your signed consent form in the post to the Trials Office. This is to allow the Trials Office to review the consent form to ensure appropriate consent procedures have been followed. Once the in-house review has been carried out and documented, the consent form at the Trials Office will be destroyed so that only your study number, initials and date of birth are stored and used to identify you.

In the Trials Office, you will be identified by a unique study number. In routine communication between your hospital and the Trials Office, you will only be identified by study number, initials and date of birth. Data may be provided to the Trials Office on paper or electronically.

By taking part in the study, you will be agreeing to allow research staff from the Trial Team at the University of Birmingham to look at the study records, including your medical records. It may be necessary to allow authorised personnel from government regulatory agencies (e.g. Medicines and Healthcare products Regulatory Agency (MHRA), the Sponsor and/or NHS bodies to have access to your medical and research records. This is to ensure that the study is being conducted to the highest possible standards. Anonymised data from the study may also be provided to other third parties (e.g. the manufacturer of the trial treatment) for research, safety monitoring or licensing purposes.

In addition, if you have provided blood samples for the study, your study number and date of birth will be passed on to personnel at external (to your local hospital) blood testing laboratories to help them identify the blood samples.

From time to time we may be asked to share the trial information (data) we have collected with researchers running other studies in this organisation and in other organisations so that they can perform analysis on the data to answer other important questions about ALS. These organisations may be universities, NHS organisations or companies involved in health research and may be in this country or abroad. Any such request is carefully considered by the study researchers and will only be granted if the necessary procedures and approvals are in place. This information will not identify you and will not be combined with other information in a way that could identify you. The information will only be used for the purpose of health research, and cannot be used to contact you or to affect your care. It will not be used to make decisions about future services available to you, such as insurance.

SAMPLE

Under no circumstances will you be identified in any way in any report, presentation or publication arising from this or any other study.

All individuals who have access to your information have a duty of confidentiality to you.
You can withdraw your consent to our processing of your data at any time. Your rights to access change or move your information are limited, as we need to manage your information in specific ways in order for the research to be reliable and accurate. If you withdraw from the study, we will keep the information about you that we have already obtained. To safeguard your rights, we will use the minimum personally-identifiable information possible. Under the provisions of the General Data Protection Regulation (GDPR) 2018, you have the right to know what information the Trials Office has recorded about you. If you wish to view this information, or find more about how we use this information, please contact Legal Services at the address below.

Legal Services
University of Birmingham
Edgbaston
Birmingham, B15 2TT

Involvement of your General Practitioner (GP)

With your permission, we will inform your GP that you are taking part in this study.

6. What will happen to samples I give?

What will happen to the samples I give?

Throughout the course of your treatment, blood samples will be taken for research and analysis. The majority of the blood samples you give are to allow the research team to monitor your safety and will be analysed in laboratories at your hospital and discarded once the necessary results have been obtained.

SAMPLE

Blood and urine samples taken for research purposes will be sent for analysis and include tests to see if and how the treatment is working, and to help us understand the different mechanisms of action within the body. These details will remain confidential within the study team.

All samples will be labelled with a unique number and transported by courier to the appropriate laboratories both in the United Kingdom (UK) at the University of Birmingham and elsewhere within Europe including laboratories in Sweden. These details will remain confidential within the study team.

Any remaining blood samples at the end of the study will also be sent to a central biorepository for long-term storage at the University of Birmingham. These samples may be very useful for research in the future and will help us to understand more about the disease. Such future research could be conducted by researchers in the UK or abroad, and may be carried out by academic institutions (e.g. universities) or commercial organisations (e.g. pharmaceutical companies). By giving your consent for your blood samples to be collected and stored, you will be offering your samples as a gift. The samples are stored under strict security and are given a code, so that researchers receiving the samples do not know your name or any other personal details. Researchers who wish to use the samples will only be given access to the samples after their research has been approved by an independent Research Ethics Committee (REC) who make sure that the research is in the interest of patients and is carried out ethically. The use of any remaining samples at the end of the study is completely optional and you therefore have the right to decline the use of these samples for any further research (see OPTIONAL statements on the ALS Study Informed Consent Form) and this will not affect your participation in the clinical trial.

During and after the study, you are the owner of the samples. This gives you the right to have any remaining sample material destroyed (by the Sponsor) at any time. If your individual sample(s) have already been processed as part of this trial at the time of your request, the results from this analysis will remain part of the information collected as part of this study. It is important to note that there may not be any samples remaining as some of these tests use the complete sample and may result in the sample being destroyed as part of the test procedure. If you choose to have any remaining samples destroyed, please contact your study doctor. Whether or not you agree to this further use will not affect your participation in the clinical trial.

Will any genetic tests be done?

SAMPLE

No. No genetic tests will be conducted in this study.

However, if you agree any remaining samples at the end of the study can be stored for use in future research studies and this may include genetic studies. Genetic studies look for changes in a person`s genetic make-up, in particular looking at chromosomes (the packages of genetic material) and changes in genes (the individual genetic instructions). This is an optional statement on the ALS Informed Consent Form and you therefore have the right to decline the use of these samples and it will not affect your participation in the clinical trial.

7. What will happen to the results of the study?

At the end of the study, the information collected will be analysed and published in recognised medical journals. The identity of the patients who took part in the study will remain confidential.

Should you wish to discuss the results of the study, you should contact your study doctor; you will have the opportunity if you wish to be informed of the results of the study once fully analysed.

8. Organising and Funding

Who is organising and funding the study?

This research is being funded by an educational grant from industry (TikoMed). The study is being sponsored by the University of Birmingham and coordinated by the D^3B trial management team, part of the Cancer Research UK Clinical Trials Unit (CRCTU) at University of Birmingham.

Who has reviewed this study?

All research in the NHS is looked at by an independent group of people called a Research Ethics Committee (REC) to protect your safety, rights, wellbeing and dignity. This study has been reviewed and given favourable opinion by the South Central – Oxford B Research Ethics Committee and by the NHS Health Research Authority (HRA). While the study is ongoing, the results will be reviewed by an

independent Data Monitoring Committee (DMC) to ensure that it is appropriate to continue with the study.

9. Further information and contact details

If you have any questions or concerns about your disease or this study, please discuss them with your doctor. You can get in touch with the doctors and nurses at any time to discuss any doubts or worries you may have about the study, and we will give you a card with contact details. Contact details are also shown below:

Principal Investigator: <Insert name of PI> **Tel:** <Insert PI tel. number>

Research Nurse: <Insert name of Research Nurse> **Tel:** <Insert nurse tel. number>

24 hour Hospital Details: <Insert 24 hour emergency contact details>

SAMPLE
