# Supplementary material for: A low molecular weight dextran sulphate, ILB®, for the treatment of amyotrophic lateral sclerosis (ALS): An open-label, single-arm, single-centre, phase II trial
Source: PLoS One. 2024 Jul 11;19(7):e0291285. doi: 10.1371/journal.pone.0291285 (PMC11239073; doi:10.1371/journal.pone.0291285)
Supplement: S3 Appendix — Pre-analytical quality control indicators collected during sample processing. (DOCX) [file pone.0291285.s003.docx]

# S4 Appendix. Pre-analytical sample quality indicators

A deviation checklist was completed for each sample taken. The following deviations were logged:

1. Activity log form error
2. Un-notified lab manual amendments
3. Insufficient volume of sample
4. Equipment failure/ lack of equipment
5. Incorrect samples
6. Sample haemolysed
7. Unlabelled sample
8. Protocol error - incorrect processing
9. Missing samples
10. Protocol error - shipping & courier
11. Partially spun sample
12. Protocol error - samples arriving late into lab

If no deviation occurred, a [-] was entered into the checklists presented in tables S4A-C; a deviation is presented by [Yes].

S4A Table. Pharmacokinetic blood samples ordered by patients’ treatment duration

On the first day of ILB® administration whole blood was taken before and after injection of the trial drug.

| Number of Treatment Weeks | Number of Treatment Administrations | Samples  (hours after ILB® injection) | Deviation Checklist Number | | | | | | | | | | | |
| --- | --- | --- | --- | --- | --- | --- | --- | --- | --- | --- | --- | --- | --- | --- |
|  |  |  | 1 | 2 | 3 | 4 | 5 | 6 | 7 | 8 | 9 | 10 | 11 | 12 |
| 38 | 38 | -0.5 | - | - | - | - | - | Yes | - | - | - | - | - | - |
|  |  | 0.5 | - | - | - | - | - | Yes | - | - | - | - | - | - |
|  |  | 1 | - | - | - | - | - | - | - | - | - | - | - | - |
|  |  | 2 | - | - | - | - | - | - | - | - | - | - | - | - |
|  |  | 2.5 | - | - | - | - | - | - | - | - | - | - | - | - |
|  |  | 3 | - | - | - | - | - | - | - | - | - | - | - | - |
|  |  | 4 | - | - | - | - | - | - | - | - | - | - | - | - |
|  |  | 6 | - | - | - | - | - | - | - | - | - | - | - | - |
| 38 | 37 | -0.5 | - | - | - | - | - | - | - | - | - | - | - | - |
|  |  | 0.5 | - | - | - | - | - | Yes | - | - | - | - | - | - |
|  |  | 1 | - | - | - | - | - | Yes | - | - | - | - | - | - |
|  |  | 2 | - | - | - | - | - | - | - | - | - | - | - | - |
|  |  | 2.5 | - | - | - | - | - | Yes | - | - | - | - | - | - |
|  |  | 3 | - | - | - | - | - | - | - | - | - | - | - | - |
|  |  | 4 | - | - | - | - | - | - | - | - | - | - | - | - |
|  |  | 6 | - | - | - | - | - | Yes | - | - | - | - | - | - |
| 36 | 35 | -0.5 | - | - | - | - | - | - | - | - | - | - | - | - |
|  |  | 0.5 | - | - | - | - | - | - | - | - | - | - | - | - |
|  |  | 1 | - | - | - | - | - | - | - | - | - | - | - | - |
|  |  | 2 | - | - | - | - | - | - | - | - | - | - | - | - |
|  |  | 2.5 | - | - | - | - | - | - | - | - | - | - | - | - |
|  |  | 3 | - | - | - | - | - | - | - | - | - | - | - | - |
|  |  | 4 | - | - | - | - | - | - | - | - | - | - | - | - |
|  |  | 6 | - | - | - | - | - | - | - | - | - | - | - | - |
| 36 | 34 | -0.5 | - | - | - | - | - | - | - | - | - | - | - | - |
|  |  | 0.5 | - | - | - | - | - | - | - | - | - | - | - | - |
|  |  | 1 | - | - | - | - | - | - | - | - | - | - | - | - |
|  |  | 2 | - | - | - | - | - | - | - | - | - | - | - | - |
|  |  | 2.5 | - | - | - | - | - | - | - | - | - | - | - | - |
|  |  | 3 | - | - | - | - | - | - | - | - | - | - | - | - |
|  |  | 4 | - | - | - | - | - | - | - | - | - | - | - | - |
|  |  | 6 | - | - | - | - | - | - | - | - | - | - | - | - |
| 35 | 35 | -0.5 | - | - | - | - | - | Yes | - | - | - | - | - | - |
|  |  | 0.5 | - | - | - | - | - | - | - | - | - | - | - | - |
|  |  | 1 | - | - | - | - | - | - | - | - | - | - | - | - |
|  |  | 2 | - | - | - | - | - | - | - | - | - | - | - | - |
|  |  | 2.5 | - | - | - | - | - | - | - | - | - | - | - | - |
|  |  | 3 | - | - | - | - | - | - | - | - | - | - | - | - |
|  |  | 4 | - | - | - | - | - | - | - | - | - | - | - | - |
|  |  | 6 | - | - | - | - | - | Yes | - | - | - | - | - | - |
| 26 | 24 | -0.5 | - | - | - | - | - | Yes* | - | - | - | - | - | - |
|  |  | 0.5 | - | - | - | - | - | - | - | - | - | - | - | - |
|  |  | 1 | - | - | - | - | - | - | - | - | - | - | - | - |
|  |  | 2 | - | - | - | - | - | - | - | - | - | - | - | - |
|  |  | 2.5 | - | - | - | - | - | - | - | - | - | - | - | - |
|  |  | 3 | - | - | - | - | - | - | - | - | - | - | - | - |
|  |  | 4 | - | - | - | - | - | - | - | - | - | - | - | - |
|  |  | 6 | - | - | - | - | - | - | - | - | - | - | - | - |
| 22 | 21 | -0.5 | - | - | - | - | - | Yes | - | - | - | - | - | - |
|  |  | 0.5 | - | - | - | - | - | - | - | - | - | - | - | - |
|  |  | 1 | - | - | - | - | - | - | - | - | - | - | - | - |
|  |  | 2 | - | - | - | - | - | - | - | - | - | - | - | - |
|  |  | 2.5 | - | - | - | - | - | - | - | - | - | - | - | - |
|  |  | 3 | - | - | - | - | - | - | - | - | - | - | - | - |
|  |  | 4 | - | - | - | - | - | - | - | - | - | - | - | - |
|  |  | 6 | - | - | - | - | - | Yes | - | - | - | - | - | - |
| 21 | 21 | -0.5 | - | - | - | - | - | - | - | - | - | - | - | - |
|  |  | 0.5 | - | - | - | - | - | - | - | - | - | - | - | - |
|  |  | 1 | - | - | - | - | - | - | - | - | - | - | - | - |
|  |  | 2 | - | - | - | - | - | - | - | - | - | - | - | - |
|  |  | 2.5 | - | - | - | - | - | - | - | - | - | - | - | - |
|  |  | 3 | - | - | - | - | - | - | - | - | - | - | - | - |
|  |  | 4 | - | - | - | - | - | - | - | - | - | - | - | - |
|  |  | 6 | - | - | - | - | - | - | - | - | - | - | - | - |
| 10 | 7 | -0.5 | - | - | - | - | - | - | - | - | - | - | - | - |
|  |  | 0.5 | - | - | - | - | - | - | - | - | - | - | - | - |
|  |  | 1 | - | - | - | - | - | - | - | - | - | - | - | - |
|  |  | 2 | - | - | - | - | - | - | - | - | - | - | - | - |
|  |  | 2.5 | - | - | - | - | - | - | - | - | Yes | - | - | - |
|  |  | 3 | - | - | - | - | - | - | - | - | Yes | - | - | - |
|  |  | 4 | - | - | - | - | - | - | - | - | Yes | - | - | - |
|  |  | 6 | - | - | - | - | - | - | - | - | Yes | - | - | - |
| 6 | 6 | -0.5 | - | - | - | - | - | - | - | - | - | - | - | - |
|  |  | 0.5 | - | - | - | - | - | - | - | - | - | - | - | - |
|  |  | 1 | - | - | - | - | - | - | - | - | - | - | - | - |
|  |  | 2 | - | - | - | - | - | - | - | - | - | - | - | - |
|  |  | 2.5 | - | - | - | - | - | - | - | - | - | - | - | - |
|  |  | 3 | - | - | - | - | - | - | - | - | - | - | - | - |
|  |  | 4 | - | - | - | - | - | - | - | - | - | - | - | - |
|  |  | 6 | - | - | - | - | - | - | - | - | - | - | - | - |
| 4 | 4 | -0.5 | - | - | - | - | - | - | - | - | - | - | - | - |
|  |  | 0.5 | - | - | - | - | - | - | - | - | - | - | - | - |
|  |  | 1 | - | - | - | - | - | - | - | - | - | - | - | - |
|  |  | 2 | - | - | - | - | - | - | - | - | - | - | - | - |
|  |  | 2.5 | - | - | - | - | - | - | - | - | - | - | - | - |
|  |  | 3 | - | - | - | - | - | - | - | - | - | - | - | - |
|  |  | 4 | - | - | - | - | - | - | - | - | - | - | - | - |
|  |  | 6 | - | - | - | - | - | - | - | - | - | - | - | - |

* Alternative non-haemolysed sample was available from the patient.

S4B Table. NfL plasma samples

Plasma samples were taken on weeks 1, 5, 10, 24 and 38 of ILB® treatment (where possible), and two weeks after the last treatment visit.

| Number of Treatment Weeks | Number of Treatment Administrations | Visit | Deviation Checklist Number | | | | | | | | | | | |
| --- | --- | --- | --- | --- | --- | --- | --- | --- | --- | --- | --- | --- | --- | --- |
|  |  |  | 1 | 2 | 3 | 4 | 5 | 6 | 7 | 8 | 9 | 10 | 11 | 12 |
| 38 | 38 | Week 1 ILB® | - | - | - | - | - | Yes | - | - | - | - | - | - |
|  |  | Week 5 ILB® | - | - | - | - | - | - | - | - | - | - | - | - |
|  |  | Week 10 ILB® | - | - | - | - | - | - | - | - | - | - | - | - |
|  |  | Week 24 ILB® | - | - | - | - | - | - | - | - | - | - | - | - |
|  |  | Week 38 ILB® | - | - | - | - | - | - | - | - | - | - | - | - |
|  |  | End of Treatment | - | - | - | - | - | - | - | - | - | - | - | - |
| 38 | 37 | Week 1 ILB® | - | - | - | - | - | - | - | - | - | - | - | - |
|  |  | Week 5 ILB® | - | - | - | - | - | - | - | - | - | - | - | - |
|  |  | Week 10 ILB® | - | - | - | - | - | - | - | - | - | - | - | - |
|  |  | Week 24 ILB® | - | - | - | - | - | - | - | - | - | - | - | - |
|  |  | Week 38 ILB® | - | - | - | - | - | - | - | - | - | - | - | - |
|  |  | End of Treatment | - | - | - | - | - | - | - | - | - | - | - | - |
| 36 | 35 | Week 1 ILB® | - | - | - | - | - | - | - | - | - | - | - | - |
|  |  | Week 5 ILB® | - | - | - | - | - | - | - | - | - | - | - | - |
|  |  | Week 10 ILB® | - | - | - | - | - | - | - | - | - | - | - | - |
|  |  | Week 24 ILB® | - | - | - | - | - | - | - | - | - | - | - | - |
|  |  | End of Treatment | - | - | - | - | - | - | - | - | - | - | - | - |
| 36 | 34 | Week 1 ILB® | - | - | - | - | - | - | - | - | - | - | - | - |
|  |  | Week 5 ILB® | - | - | - | - | - | - | - | - | - | - | - | - |
|  |  | Week 10 ILB® | - | - | - | - | - | - | - | - | - | - | - | - |
|  |  | Week 24 ILB® | - | - | - | - | - | - | - | - | - | - | - | - |
|  |  | End of Treatment | - | - | - | - | - | - | - | - | - | - | - | - |
| 35 | 35 | Week 1 ILB® | - | - | - | - | - | Yes | - | - | - | - | - | - |
|  |  | Week 5 ILB® | - | - | - | - | - | - | - | - | - | - | - | - |
|  |  | Week 10 ILB® | - | - | - | - | - | - | - | - | - | - | - | - |
|  |  | Week 24 ILB® | - | - | - | - | - | - | - | - | - | - | - | - |
|  |  | End of Treatment | - | - | - | - | - | - | - | - | - | - | - | - |
| 26 | 24 | Week 1 ILB® | - | - | - | - | - | - | - | - | - | - | - | - |
|  |  | Week 5 ILB® | - | - | - | - | - | - | - | - | - | - | - | - |
|  |  | Week 10 ILB® | - | - | - | - | - | - | - | - | - | - | - | - |
|  |  | Week 24 ILB® | - | - | - | - | - | - | - | - | - | - | - | - |
|  |  | End of Treatment | - | - | - | - | - | - | - | - | - | - | - | - |
| 22 | 21 | Week 1 ILB® | - | - | - | - | - | Yes | - | - | - | - | - | - |
|  |  | Week 5 ILB® | - | - | - | - | - | - | - | - | - | - | - | - |
|  |  | Week 10 ILB® | - | - | - | - | - | - | - | Yes | - | - | - | - |
|  |  | End of Treatment | - | - | - | - | - | - | - | - | - | - | - | - |
| 21 | 21 | Week 1 ILB® | - | - | - | - | - | - | - | - | - | - | - | - |
|  |  | Week 5 ILB® | - | - | - | - | - | - | - | - | - | - | - | - |
|  |  | Week 10 ILB® | - | - | - | - | - | - | - | Yes | - | - | - | - |
|  |  | End of Treatment | - | - | - | - | - | - | - | - | - | - | - | - |
| 10 | 7 | Week 1 ILB® | - | - | - | - | - | - | - | - | - | - | - | - |
|  |  | Week 5 ILB® | - | - | - | - | - | - | - | - | - | - | - | - |
|  |  | Week 10 ILB® | - | - | - | - | - | - | - | - | - | - | - | - |
|  |  | End of Treatment | - | - | - | - | - | - | - | - | - | - | - | - |
| 6 | 6 | Week 1 ILB® | - | - | - | - | - | - | - | - | - | - | - | - |
|  |  | Week 5 ILB® | - | - | - | - | - | - | - | Yes | - | - | - | - |
|  |  | Week 10 ILB® | - | - | - | - | - | - | - | - | - | - | - | - |
|  |  | End of Treatment | - | - | - | - | - | - | - | - | - | - | - | - |
| 4 | 4 | Week 1 ILB® | - | - | - | - | - | - | - | - | - | - | - | - |
|  |  | Week 5 ILB® | - | - | - | - | - | - | - | - | - | - | - | - |
|  |  | Week 10 ILB® | - | - | - | - | - | - | - | - | - | - | - | - |
|  |  | End of Treatment | - | - | - | - | - | - | - | - | - | - | - | - |

S4C Table. Urine samples

Urine samples were taken on weeks 1, 5, 10, 24 and 38 of ILB® treatment (where possible), and two weeks after the last treatment visit.

| Number of Treatment Weeks | Number of Treatment Administrations | Visit | Deviation Checklist Number | | | | | | | | | | | |
| --- | --- | --- | --- | --- | --- | --- | --- | --- | --- | --- | --- | --- | --- | --- |
|  |  |  | 1 | 2 | 3 | 4 | 5 | 6 | 7 | 8 | 9 | 10 | 11 | 12 |
| 38 | 38 | Week 1 ILB® | - | - | - | - | - | - | - | - | Yes | - | - | - |
|  |  | Week 5 ILB® | - | - | - | - | - | - | - | - | - | - | - | - |
|  |  | Week 10 ILB® | - | - | - | - | - | - | - | - | - | - | - | - |
|  |  | Week 24 ILB® | - | - | - | - | - | - | - | - | Yes | - | - | - |
|  |  | End of Treatment | - | - | - | - | - | - | - | - | - | - | - | - |
|  |  | Week 38 ILB® | - | - | - | - | - | - | - | - | - | - | - | - |
| 38 | 37 | Week 1 ILB® | - | - | - | - | - | - | - | - | - | - | - | - |
|  |  | Week 5 ILB® | - | - | - | - | - | - | - | - | - | - | - | - |
|  |  | Week 10 ILB® | - | - | - | - | - | - | - | - | - | - | - | - |
|  |  | Week 24 ILB® | - | - | - | - | - | - | - | - | - | - | - | - |
|  |  | Week 38 ILB® | - | - | - | - | - | - | - | - | - | - | - | - |
|  |  | End of Treatment | - | - | - | - | - | - | - | - | - | - | - | - |
| 36 | 35 | Week 1 ILB® | - | - | - | - | - | - | - | - | - | - | - | - |
|  |  | Week 5 ILB® | - | - | - | - | - | - | - | - | - | - | - | - |
|  |  | Week 10 ILB® | - | - | - | - | - | - | - | - | - | - | - | - |
|  |  | Week 24 ILB® | - | - | - | - | - | - | - | - | - | - | - | - |
|  |  | End of Treatment | - | - | - | - | - | - | - | - | - | - | - | - |
| 36 | 34 | Week 1 ILB® | - | - | - | - | - | - | - | - | - | - | - | - |
|  |  | Week 5 ILB® | - | - | - | - | - | - | - | - | - | - | - | - |
|  |  | Week 10 ILB® | - | - | - | - | - | - | - | - | - | - | - | - |
|  |  | Week 24 ILB® | - | - | - | - | - | - | - | - | - | - | - | - |
|  |  | End of Treatment | - | - | - | - | - | - | - | - | - | - | - | - |
| 35 | 35 | Week 1 ILB® | - | - | - | - | - | - | - | - | - | - | - | - |
|  |  | Week 5 ILB® | - | - | - | - | - | - | - | - | - | - | - | - |
|  |  | Week 10 ILB® | - | - | - | - | - | - | - | - | - | - | - | - |
|  |  | Week 24 ILB® | - | - | - | - | - | - | - | - | - | - | - | - |
|  |  | End of Treatment | - | - | - | - | - | - | - | - | - | - | - | - |
| 26 | 24 | Week 1 ILB® | - | - | - | - | - | - | - | - | - | - | - | - |
|  |  | Week 5 ILB® | - | - | - | - | - | - | - | - | - | - | - | - |
|  |  | Week 10 ILB® | - | - | - | - | - | - | - | - | - | - | - | - |
|  |  | Week 24 ILB® | - | - | - | - | - | - | - | - | - | - | - | - |
|  |  | End of Treatment | - | - | - | - | - | - | - | - | - | - | - | - |
| 22 | 21 | Week 1 ILB® | - | - | - | - | - | - | - | - | - | - | - | - |
|  |  | Week 5 ILB® | - | - | - | - | - | - | - | - | - | - | - | - |
|  |  | Week 10 ILB® | - | - | - | - | - | - | - | - | - | - | - | - |
|  |  | End of Treatment | - | - | - | - | - | - | - | - | - | - | - | - |
| 21 | 21 | Week 1 ILB® | - | - | - | - | - | - | - | - | - | - | - | - |
|  |  | Week 5 ILB® | - | - | - | - | - | - | - | - | - | - | - | - |
|  |  | Week 10 ILB® | - | - | - | - | - | - | - | - | - | - | - | - |
|  |  | End of Treatment | - | - | - | - | - | - | - | - | - | - | - | - |
| 1 | 7 | Week 1 ILB® | - | - | - | - | - | - | - | - | - | - | - | - |
|  |  | Week 5 ILB® | - | - | - | - | - | - | - | - | - | - | - | - |
|  |  | Week 10 ILB® | - | - | - | - | - | - | - | - | - | - | - | - |
|  |  | End of Treatment | - | - | - | - | - | - | - | - | Yes | - | - | - |
| 6 | 6 | Week 1 ILB® | - | - | - | - | - | - | - | - | - | - | - | - |
|  |  | Week 5 ILB® | - | - | - | - | - | - | - | - | - | - | - | - |
|  |  | Week 10 ILB® | - | - | - | - | - | - | - | - | - | - | - | - |
|  |  | End of Treatment | - | - | - | - | - | - | - | - | - | - | - | - |
| 4 | 4 | Week 1 ILB® | - | - | - | - | - | - | - | - | Yes | - | - | - |
|  |  | Week 5 ILB® | - | - | - | - | - | - | - | - | - | - | - | - |
|  |  | Week 10 ILB® | - | - | - | - | - | - | - | - | - | - | - | - |
|  |  | End of Treatment | - | - | - | - | - | - | - | - | - | - | - | - |
