# Supplementary material for: A low molecular weight dextran sulphate, ILB®, for the treatment of amyotrophic lateral sclerosis (ALS): An open-label, single-arm, single-centre, phase II trial
Source: PLoS One. 2024 Jul 11;19(7):e0291285. doi: 10.1371/journal.pone.0291285 (PMC11239073; doi:10.1371/journal.pone.0291285)
Supplement: S4 Appendix — S5 Table A-C indicate the Bias, imprecision, and total error (within levels of quantification) of the Calibrators used for the heparin red assay to determine the pharmacokinetics of ILB®. All data are expressed as a percentage of the expected value. Unacceptable high error rates (systematic or random error) are highlighted in red. S5 Table D-F indicate the Bias, imprecision, and total error of the Internal QC samples used for the heparin red assay to determine the pharmacokinetics of ILB®. All data are expressed as a percentage of the expected value. Unacceptable high error rates (systematic or random error) are highlighted in red. The colour gradient from green to red in S5 Table E represents the quality gradient of the internal control samples (green: good; red: poor). (DOCX) [file pone.0291285.s004.docx]

# S5 Appendix. Quality control data for the heparin red assay used for ILB® pharmacokinetic measurements

S5A Table

S5B Table

Table S5C

S5D Table

S5E Table

S5F Table
