# Supplementary material for: A low molecular weight dextran sulphate, ILB®, for the treatment of amyotrophic lateral sclerosis (ALS): An open-label, single-arm, single-centre, phase II trial
Source: PLoS One. 2024 Jul 11;19(7):e0291285. doi: 10.1371/journal.pone.0291285 (PMC11239073; doi:10.1371/journal.pone.0291285)
Supplement: S5 Appendix — S6 Table A-C indicate the Bias, imprecision, and total error (within levels of quantification) of the Calibrators used for the HGF assay on the pharmacokinetic samples. All data are expressed as a percentage of the expected value. (DOCX) [file pone.0291285.s005.docx]

# S6 Appendix. Quality control data for ELISA used for HGF pharmacokinetic measurements

S6A Table

| Bias HGF Calibrator value (pg/ml) | Average | Min | Max | Stdev |  | P001 | P002 | P003 | P004 | P005 |
| --- | --- | --- | --- | --- | --- | --- | --- | --- | --- | --- |
| 10000.00 | 0.03 | 0.01 | 0.06 | 0.02 |  | 0.06 |  | 0.02 | 0.03 | 0.01 |
| 5000.00 | 0.18 | 0.10 | 0.27 | 0.07 |  | 0.21 | 0.27 | 0.12 | 0.10 | 0.21 |
| 2500.00 | 0.32 | 0.02 | 0.76 | 0.33 |  | 0.76 | 0.02 | 0.56 | 0.16 | 0.09 |
| 1250.00 | 1.03 | 0.11 | 1.74 | 0.75 |  | 0.11 | 1.74 | 1.62 | 0.34 | 1.36 |
| 625.00 | 1.91 | 0.07 | 4.04 | 1.61 |  | 0.81 | 4.04 | 2.97 | 1.64 | 0.07 |
| 312.50 | 2.89 | 0.28 | 8.55 | 3.30 |  | 2.61 | 8.55 | 2.20 | 0.84 | 0.28 |
| 156.25 | 2.00 | 0.64 | 3.00 | 0.97 |  | 2.84 | 3.00 | 1.52 | 0.64 | 2.02 |
| 0.00 |  |  |  |  |  |  |  |  |  |  |
| Average per plate | 1.28 | 0.54 | 2.94 | 0.98 |  | 1.06 | 2.94 | 1.29 | 0.54 | 0.58 |

S6B Table

| Variance HGF Calibrator value (pg/ml) | Average | Min | Max | Stdev |  | P001 | P002 | P003 | P004 | P005 |
| --- | --- | --- | --- | --- | --- | --- | --- | --- | --- | --- |
| 10000.00 | 1.30 | 0.19 | 1.86 | 0.77 |  | 1.86 |  | 1.35 | 1.80 | 0.19 |
| 5000.00 | 4.21 | 0.77 | 9.12 | 3.51 |  | 9.12 | 3.64 | 0.77 | 1.24 | 6.27 |
| 2500.00 | 5.56 | 1.22 | 11.12 | 4.92 |  | 1.22 | 10.74 | 2.34 | 2.39 | 11.12 |
| 1250.00 | 8.19 | 3.44 | 14.62 | 4.04 |  | 7.12 | 14.62 | 7.96 | 3.44 | 7.80 |
| 625.00 | 5.25 | 1.19 | 13.93 | 5.25 |  | 6.42 | 13.93 | 1.19 | 1.84 | 2.84 |
| 312.50 | 5.24 | 2.37 | 7.03 | 1.83 |  | 7.03 | 6.60 | 4.92 | 2.37 | 5.27 |
| 156.25 | 5.65 | 2.30 | 12.50 | 4.40 |  | 7.68 | 12.50 | 2.87 | 2.90 | 2.30 |
| 0.00 |  |  |  |  |  |  |  |  |  |  |
| Average per plate | 5.31 | 2.28 | 10.34 | 3.15 |  | 5.78 | 10.34 | 3.06 | 2.28 | 5.11 |

S6C Table

| TE HGF Calibrator value (pg/ml) | Average | Min | Max | Stdev |  | P001 | P002 | P003 | P004 | P005 |
| --- | --- | --- | --- | --- | --- | --- | --- | --- | --- | --- |
| 10000.00 | 1.33 | 0.20 | 1.92 | 0.79 |  | 1.92 |  | 1.37 | 1.83 | 0.20 |
| 5000.00 | 4.39 | 0.89 | 9.34 | 3.56 |  | 9.34 | 3.91 | 0.89 | 1.35 | 6.48 |
| 2500.00 | 5.88 | 1.97 | 11.21 | 4.67 |  | 1.97 | 10.75 | 2.90 | 2.55 | 11.21 |
| 1250.00 | 9.22 | 3.78 | 16.35 | 4.60 |  | 7.23 | 16.35 | 9.58 | 3.78 | 9.16 |
| 625.00 | 7.15 | 2.91 | 17.97 | 6.27 |  | 7.23 | 17.97 | 4.17 | 3.48 | 2.91 |
| 312.50 | 8.13 | 3.20 | 15.14 | 4.57 |  | 9.64 | 15.14 | 7.11 | 3.20 | 5.56 |
| 156.25 | 7.65 | 3.54 | 15.50 | 5.21 |  | 10.51 | 15.50 | 4.39 | 3.54 | 4.32 |
| 0.00 |  |  |  |  |  |  |  |  |  |  |
| Average per plate | 6.59 | 2.82 | 13.27 | 4.02 |  | 6.83 | 13.27 | 4.34 | 2.82 | 5.69 |
