# Supplementary material for: A low molecular weight dextran sulphate, ILB®, for the treatment of amyotrophic lateral sclerosis (ALS): An open-label, single-arm, single-centre, phase II trial
Source: PLoS One. 2024 Jul 11;19(7):e0291285. doi: 10.1371/journal.pone.0291285 (PMC11239073; doi:10.1371/journal.pone.0291285)
Supplement: S6 Appendix — S7 Table A-C show the dose of ILB® (mg) administered at each treatment visit during the initial 10-week treatment period, the first treatment extension (weeks 11–24), and the second treatment extension (weeks 25–48), respectively. Due to the premature termination of the trial due to the COVID-19 pandemic, no patient received treatment past 38 weeks. (DOCX) [file pone.0291285.s006.docx]

# S7 Appendix. ILB® doses administered during the ALS trial

S7A Table. Dose of ILB® (mg) administered at each treatment visit during the initial 10-week treatment period ordered by patients’ treatment duration

| Duration of treatment* | Total Dose Administered (mg) | | | | | | | | | |
| --- | --- | --- | --- | --- | --- | --- | --- | --- | --- | --- |
|  | Week 1 | Week 2 | Week 3 | Week 4 | Week 5 | Week 6 | Week 7 | Week 8 | Week 9 | Week 10 |
| 38 (38) | 173 | 174 | 173 | 174 | 172 | 174 | 174 | 174 | 173 | 173 |
| 38 (37) | 194 | 194 | 192 | 191 | 193 | 191 | 191 | 189 | 192 | 191 |
| 36 (35) | 181 | 181 | 181 | 181 | 181 | 179 | 180 | 179 | 177 | 177 |
| 36 (34) | 143 | - | 140 | 140 | 140 | 144 | 140 | 140 | 140 | 140 |
| 35 (35) | 150 | 150 | 151 | 150 | 150 | 152 | 152 | 152 | 153 | 150 |
| 26 (24) | 180 | 181 | 181 | - | 180 | 180 | 178 | 179 | 177 | 180 |
| 22 (21) | 148 | 148 | 147 | 147 | 147 | - | 144 | 142 | 141 | 143 |
| 21 (21) | 137 | 134 | 134 | 135 | 134 | 134 | 134 | 135 | 135 | 133 |
| 10 (7) | 119 | 121 | 121 | 120 | - | - | - | 118 | 118 | 117 |
| 6 (6) | 167 | 169 | 171 | 168 | 171 | 170 | - | - | - | - |
| 4 (4) | 187 | 185 | 186 | 187 | - | - | - | - | - | - |

* Data presented as N(n); where N = number of weeks on treatment; and n = total number of treatment administrations.

S7B Table. Dose of ILB® (mg) administered at each treatment visit during the first treatment extension (weeks 11-24)

| Duration of treatment* | Total Dose Administered (mg) | | | | | | | | | | | | | |
| --- | --- | --- | --- | --- | --- | --- | --- | --- | --- | --- | --- | --- | --- | --- |
|  | Week 11 | Week 12 | Week 13 | Week 14 | Week 15 | Week 16 | Week 17 | Week 18 | Week 19 | Week 20 | Week 21 | Week 22 | Week 23 | Week 24 |
| 38 (38) | 175 | 174 | 173 | 174 | 172 | 173 | 174 | 174 | 173 | 172 | 173 | 171 | 173 | 170 |
| 38 (37) | 184 | 182 | - | 186 | 185 | 183 | 180 | 181 | 181 | 179 | 177 | 176 | 176 | 176 |
| 36 (35) | 177 | 175 | 175 | 176 | 174 | 176 | 176 | 177 | 178 | 176 | 178 | 176 | - | 177 |
| 36 (34) | 144 | 144 | 144 | 143 | - | 145 | 145 | 145 | 144 | 143 | 144 | 145 | 144 | 145 |
| 35 (35) | 154 | 153 | 154 | 154 | 154 | 152 | 152 | 152 | 152 | 150 | 152 | 152 | 152 | 153 |
| 26 (24) | 178 | 178 | - | 176 | 177 | 175 | 175 | 174 | 171 | 170 | 170 | 170 | 169 | 167 |
| 22 (21) | 142 | 140 | 139 | 140 | 139 | 139 | 138 | 137 | 136 | 136 | 137 | 137 | - | - |
| 21 (21) | 134 | 133 | 132 | 133 | 132 | 131 | 130 | 130 | 131 | 129 | 129 | - | - | - |
| 10 (7) | - | - | - | - | - | - | - | - | - | - | - | - | - | - |
| 6 (6) | - | - | - | - | - | - | - | - | - | - | - | - | - | - |
| 4 (4) | - | - | - | - | - | - | - | - | - | - | - | - | - | - |

* Data presented as N(n); where N = number of weeks on treatment; and n = total number of treatment administrations.

S7C Table. Dose of ILB® (mg) administered at each treatment visit during the second treatment extension (weeks 25-38)

| Duration of treatment* | Total Dose Administered (mg) | | | | | | | | | | | | | |
| --- | --- | --- | --- | --- | --- | --- | --- | --- | --- | --- | --- | --- | --- | --- |
|  | Week 25 | Week 26 | Week 27 | Week 28 | Week 29 | Week 30 | Week 31 | Week 32 | Week 33 | Week 34 | Week 35 | Week 36 | Week 37 | Week 38 |
| 38 (38) | 175 | 175 | 174 | 172 | 176 | 171 | 172 | 170 | 169 | 168 | 169 | 168 | 168 | 169 |
| 38 (37) | 175 | 174 | 174 | 173 | 173 | 172 | 171 | 172 | 170 | 171 | 171 | 170 | 169 | 169 |
| 36 (35) | 178 | 177 | 177 | 178 | 178 | 178 | 178 | 178 | 179 | 178 | 179 | 176 | - | - |
| 36 (34) | 145 | 145 | 145 | 146 | 146 | 145 | 146 | 145 | 145 | 145 | 144 | 144 | - | - |
| 35 (35) | 152 | 151 | 151 | 150 | 150 | 150 | 150 | 147 | 146 | 147 | 147 | - | - | - |
| 26 (24) | - | - | - | - | - | - | - | - | - | - | - | - | - | - |
| 22 (21) | - | - | - | - | - | - | - | - | - | - | - | - | - | - |
| 21 (21) | - | - | - | - | - | - | - | - | - | - | - | - | - | - |
| 10 (7) | - | - | - | - | - | - | - | - | - | - | - | - | - | - |
| 6 (6) | - | - | - | - | - | - | - | - | - | - | - | - | - | - |
| 4 (4) | - | - | - | - | - | - | - | - | - | - | - | - | - | - |

* Data presented as N(n); where N = number of weeks on treatment; and n = total number of treatment administrations
