# Supplementary material for: A low molecular weight dextran sulphate, ILB®, for the treatment of amyotrophic lateral sclerosis (ALS): An open-label, single-arm, single-centre, phase II trial
Source: PLoS One. 2024 Jul 11;19(7):e0291285. doi: 10.1371/journal.pone.0291285 (PMC11239073; doi:10.1371/journal.pone.0291285)
Supplement: S7 Appendix — Pharmacokinetics for ILB® detected within the ALS trial. (DOCX) [file pone.0291285.s007.docx]

# S10 Appendix. Additional pharmacokinetic data of ILB®

## S10A Table. Pharmacokinetics of ILB® per patient ordered on patients’ treatment duration

| Duration of treatment* | AUC_0-last_ | C_max_ | T_max_ | t_1/2_ |
| --- | --- | --- | --- | --- |
| 36 (35) | 23.6 | 5.0 | 2.0 | 3.1 |
| 36 (34) | 29.5 | 7.0 | 2.0 | 5.6 |
| 26 (24) | 22.5 | 5.2 | 3.0 | -^&^ |
| 21 (21) | 27.6 | 7.0 | 2.5 | 2.6^^^ |
| 6 (6) | 22.8 | 4.7 | 2.5 | -^&^ |
| 4 (4) | 37.1 | 7.2 | 2.5 | -^&^ |

* Data presented as N(n); where N = number of weeks on treatment; and n = total number of treatment administrations.

^&^ t_1/2_ could not be calculated (see requirements in the “Statistical analysis” subsection of the Methods).

^^^ The sample prior to ILB® administration for this patient was not analysed/missing but all other samples were collected and have, therefore, been included.

AUC, area under the curve; C_max_, maximum concentration; t_1/2_, half-life; T_max_, time to the maximum concentration.

Notes: Results from four patients were not included due to haemolysis of the plasma in some of their samples. In addition, the results from one patient were not included due to an incomplete pharmacokinetic sample series.

## S10B Table. Summary pharmacokinetics of ILB®

|  | AUC_0-last_ | C_max_ | T_max_ | t_1/2_ |
| --- | --- | --- | --- | --- |
| N | 6 | 6 | 6 | 3 |
| Mean (S.D.) | 27.2 (5.6) | 6.0 (1.2) | 2.4 (0.4) | 3.7 (1.6) |
| Median | 25.6 | 6.1 | 2.5 | 3.1 |
| IQR | (23.0, 29.0) | (5.1, 7.0) | (2.1, 2.5) | - |
| Range | (22.5, 37.1) | (4.7, 7.2) | (2.0, 3.0) | (2.6, 5.6) |

AUC, area under the curve; C_max_, maximum concentration; IQR, interquartile range; S.D., standard deviation; t_1/2_, half-life; T_max_, time to at the maximum concentration.

Notes: Data from samples from four patients were not included due to haemolysis of the plasma. In addition, the data from one patient were not included due to an incomplete pharmacokinetic sample series. Calculations for t_1/2_ were only possible in three patients based on the terminal phase of the pharmacokinetic curve (see the “Statistical analysis” subsection of the Methods).
