# Supplementary material for: A low molecular weight dextran sulphate, ILB®, for the treatment of amyotrophic lateral sclerosis (ALS): An open-label, single-arm, single-centre, phase II trial
Source: PLoS One. 2024 Jul 11;19(7):e0291285. doi: 10.1371/journal.pone.0291285 (PMC11239073; doi:10.1371/journal.pone.0291285)
Supplement: S8 Appendix — Pharmacokinetics for HGF detected within the ALS trial. (DOCX) [file pone.0291285.s008.docx]

# S11 Appendix. Additional pharmacokinetic data of HGF

## S11A Table. Pharmacokinetics of HGF per patient ordered on patients’ treatment duration

| Duration of treatment* | AUC_0-last_ | C_max_ | T_max_ | t_1/2_^&^ |
| --- | --- | --- | --- | --- |
| 36 (35) | 205402.8 | 41101.0 | 2.0 | - |
| 36 (34) | 292161.4 | 64415.5 | 2.0 | - |
| 26 (24) | 229245.4 | 48461.9 | 3.0 | - |
| 21 (21) | 290215.0 | 64830.56 | 2.0 | -^^^ |
| 6 (6) | 144501.0 | 31425.7 | 2.5 | - |
| 4 (4) | 315222.2 | 67683.0 | 2.0 | - |

* Data presented as N(n); where N = number of weeks on treatment; and n = total number of treatment administrations.

^&^ t_1/2_ was not calculated for all patients as the concentration of HGF in plasma did not drop below half its maximal value.

^^^ The sample prior to ILB® administration for this patient was not analysed/missing but all other samples were collected and have, therefore, been included.

AUC, area under the curve; C_max_, maximum concentration; t_1/2_, half-life; T_max_, time to maximum concentration.

Notes: Results from four patients were not included due to haemolysis of the plasma in some of their samples. In addition, results from one patient were not included due to an incomplete pharmacokinetic sample series.

## S11B Table. Summary pharmacokinetics of HGF

|  | AUC_0-last_ | C_max_ | T_max_ | t_1/2_^*^ |
| --- | --- | --- | --- | --- |
| N | 6 | 6 | 6 | - |
| Mean (S.D.) | 246124.6 (64974.4) | 52986.3 (14923.1) | 2.25 (0.42) | - |
| Median | 259730.2 | 56438.7 | 2 | - |
| IQR | (211363.4, 291674.8) | (42941.2, 64726.8) | (2, 2.38) | - |
| Range | (211363.4, 291674.8) | (42941.2, 64726.8) | (2, 2.38) | - |

* t_1/2_ could not be calculated as the concentration of HGF in plasma did not drop below half its maximal value.

AUC, area under the curve; C_max_, maximum concentration; IQR, interquartile range; S.D., standard deviation; t_1/2_, half-life; T_max_, time to maximum concentration.

Note: Data from four patients were not included due to haemolysis of the plasma in some of their samples. In addition, results from one patient were not included due to an incomplete pharmacokinetic sample series.
