# Supplementary material for: A low molecular weight dextran sulphate, ILB®, for the treatment of amyotrophic lateral sclerosis (ALS): An open-label, single-arm, single-centre, phase II trial
Source: PLoS One. 2024 Jul 11;19(7):e0291285. doi: 10.1371/journal.pone.0291285 (PMC11239073; doi:10.1371/journal.pone.0291285)
Supplement: S1 Table — Schedules for phase II liver chemistry monitoring and required follow up assessments. (DOCX) [file pone.0291285.s011.docx]

# S3 Table. Liver safety required actions and follow-up assessments

**Phase II liver chemistry increased monitoring criteria** have been designed to assure subject safety and evaluate liver event aetiology (in alignment with the FDA premarketing clinical liver safety guidance).

<https://www.fda.gov/regulatory-information/search-fda-guidance-documents/drug-induced-liver-injury-premarketing-clinical-evaluation>

| **Phase II liver chemistry increased monitoring criteria and required follow up assessments Liver Chemistry Increased Monitoring Criteria** | |
| --- | --- |
| **ALT-absolute** | ALT > 5xULN |
| **ALT Increase** | ALT > 3xULN persists for 4 weeks |
| **Bilirubin^1, 2^** | ALT > 3xULN **and** bilirubin 2xULN (>35% direct bilirubin) |
| **INR^2^** | ALT > 3xULN **and** INR>1.5, if INR measured |
| **Cannot Monitor** | ALT > 3xULN and cannot be monitored weekly for 4 weeks |
| **Symptomatic^3^** | ALT > 3xULN associated with symptoms (new or worsening) believed to be related to liver injury or hypersensitivity |
| **Required Actions and Follow up Assessments following ANY Liver Event** | |
| **Actions** | **Follow Up Assessments** |
| - Report the event to Trials Office **within 72 hours** - Complete the liver event CRF and complete an SAE - if the event also meets the criteria for an SAE - Perform liver event follow up assessments - Monitor the subject until liver chemistries resolve, stabilize, or return to within baseline (see **MONITORING** below). “Baseline” refers to the laboratory assessments performed closest and prior to dosing of study treatment.   **MONITORING:**  **For bilirubin or INR criteria:**   - Repeat liver chemistries (include ALT, AST, alkaline phosphatase, bilirubin) and perform liver event follow up assessments within 24 hrs | - Viral hepatitis serology^4^ - Blood sample for pharmacokinetic (PK) analysis, obtained within 7after an identified liver event if within 6 months post dose^5^ - Serum creatine phosphokinase (CPK) and lactate dehydrogenase (LDH) - Fractionate bilirubin, if total bilirubin 2xULN - Obtain complete blood count with differential to assess eosinophilia - Record the appearance or worsening of clinical symptoms of liver injury, or hypersensitivity, on the AE report form |
| - Monitor subjects twice weekly until liver chemistries resolve, stabilize or return to within baseline - A specialist or hepatology consultation is recommended   For All other criteria:   - Repeat liver chemistries (include ALT, AST, alkaline phosphatase, bilirubin) and perform liver event follow up assessments within 24-72 hrs - Monitor subjects weekly until liver chemistries resolve, stabilize or return to within baseline | - Record use of concomitant medications on the concomitant medications report form including acetaminophen, herbal remedies, other over the counter medications. - Record alcohol use on the liver event alcohol intake case report form   For bilirubin or INR criteria:   - Anti-nuclear antibody, anti-smooth muscle antibody, Type 1 anti-liver kidney microsomal antibodies, and quantitative total immunoglobulin G (IgG or gamma globulins). - Serum acetaminophens adduct HPLC assay (quantifies potential acetaminophen contribution to liver injury in subjects with definite or likely acetaminophen use in the preceding week [James, 2009]). - Liver imaging (ultrasound, magnetic resonance, or computerised tomography) and /or liver biopsy to evaluate liver disease; complete Liver Imaging and/or Liver Biopsy CRF forms. |

1. Serum bilirubin fractionation should be performed if testing is available. If serum bilirubin fractionation is not immediately available, discontinue study treatment for that subject if ALT 3xULN **and** bilirubin 2xULN. Additionally, if serum bilirubin fractionation testing is unavailable, **record presence of detectable urinary bilirubin on dipstick**, indicating direct bilirubin elevations and suggesting liver injury.

2. All events of ALT > 3xULN **and** bilirubin > 2xULN (>35% direct bilirubin) or ALT > 3xULN **and** INR>1.5, if INR measured which may indicate severe liver injury (possible ‘Hy’s Law’), **must be reported as an SAE (excluding studies of hepatic impairment or cirrhosis)**; INR measurement is not required and the threshold value stated will not apply to subjects receiving anticoagulants

3. New or worsening symptoms believed to be related to liver injury (such as fatigue, nausea, vomiting, right upper quadrant pain or tenderness, or jaundice) or believed to be related to hypersensitivity (such as fever, rash or eosinophilia)

4. Includes: Hepatitis A IgM antibody; Hepatitis B surface antigen and Hepatitis B Core Antibody (IgM); Hepatitis C RNA; Cytomegalovirus IgM antibody; Epstein-Barr viral capsid antigen IgM antibody (or if unavailable, obtain heterophile antibody or monospot testing); Hepatitis E IgM antibody

5. Record the date/time of the PK blood sample draw and the date/time of the last dose of study treatment prior to blood sample draw on the CRF. If the date or time of the last dose is unclear, provide the subject’s best approximation. If the date/time of the last dose cannot be approximated OR a PK sample cannot be collected in the time period indicated above, do not obtain a PK sample.
