# Supplementary material for: A low molecular weight dextran sulphate, ILB®, for the treatment of amyotrophic lateral sclerosis (ALS): An open-label, single-arm, single-centre, phase II trial
Source: PLoS One. 2024 Jul 11;19(7):e0291285. doi: 10.1371/journal.pone.0291285 (PMC11239073; doi:10.1371/journal.pone.0291285)
Supplement: S2 Table — Liver function tests measurements of a patient who, within the trial, who experienced raised valued. (DOCX) [file pone.0291285.s012.docx]

# S8 Table. Liver function test results from the suspected drug induced liver injury

|  | Screening | Week 1 | Week 2 | Week 3 | Week 4 | Week 8 | Week 9 | Week 10 | Week 12 |
| --- | --- | --- | --- | --- | --- | --- | --- | --- | --- |
| Albumin (g/L) | 37 | 39 | 37 | 38 | 37 | 35 | 37 | 38 | 41 |
|  | Normal | Normal | Normal | Normal | Normal | Normal | Normal | Normal | Normal |
| ALP (IU/L) | 225 | 229 | 169 | 138 | 145 | 312 | 199 | 268 | 292 |
|  | Abnormal clinically insignificant | Abnormal clinically insignificant | Normal | Abnormal clinically insignificant | Abnormal clinically insignificant | **Abnormal clinically significant** | Abnormal clinically insignificant | **Abnormal clinically significant** | **Abnormal clinically significant** |
| ALT (IU/L) | 60 | 57 | 51 | 63 | 44 | 99 | 72 | 168 | 122 |
|  | Abnormal clinically insignificant | Abnormal clinically insignificant | Normal | Abnormal clinically insignificant | Normal | **Abnormal clinically significant** | Abnormal clinically insignificant | **Abnormal clinically significant** | **Abnormal clinically significant** |
| AST (IU/L) | 45 | 27 | 37 | 44 | 26 | 88 | 54 | 104 | 50 |
|  | Abnormal clinically insignificant | Normal | Abnormal clinically insignificant | Abnormal clinically insignificant | Normal | **Abnormal clinically significant** | Abnormal clinically insignificant | **Abnormal clinically significant** | Abnormal clinically insignificant |
| Bilirubin (total, umol/L) | 6 | 7 | 5 | 4 | 5 | 4 | 4 | 5 | 6 |
|  | Normal | Normal | Normal | Normal | Normal | Normal | Normal | Normal | Normal |
| GGT (IU/L) | 235 | 244 | 204 | 215 | 192 | 367 | 297 | 436 | 595 |
|  | Abnormal clinically insignificant | Abnormal clinically insignificant | Abnormal clinically insignificant | Abnormal clinically insignificant | Abnormal clinically insignificant | Abnormal clinically insignificant | Abnormal clinically insignificant | Abnormal clinically insignificant | Normal |

N.B. Clinically significant test results are highlighted in bold, assuming the normal ranges for these were albumin, 35-50 g/L, alkaline phosphatase (ALP), 30-130 IU/L, alanine transaminase (ALT), 0-55 IU/L, aspartate transaminase (AST), 5-34 IU/L, bilirubin: <21 μmol/L, and gamma-glutamyl transferase (GGT), 8-33 IU/L.
