# Supplementary material for: A low molecular weight dextran sulphate, ILB®, for the treatment of amyotrophic lateral sclerosis (ALS): An open-label, single-arm, single-centre, phase II trial
Source: PLoS One. 2024 Jul 11;19(7):e0291285. doi: 10.1371/journal.pone.0291285 (PMC11239073; doi:10.1371/journal.pone.0291285)
Supplement: S3 Table — The list of adverse events reported during the ALS trial. (DOCX) [file pone.0291285.s013.docx]

# S9 Table. Adverse events

|  |  | **Number of patients affected (n) and adverse events (E) according to CTCAE toxicity [n (%), E]** | | |
| --- | --- | --- | --- | --- |
|  |  | **Grade*** | | |
| **Category** | **Toxicity** | **1** | **2** | **3** |
| Blood and lymphatic system disorders | Blood and lymphatic system disorders - Other, specify: Not clinically significant abnormal blood creatinine | 1 (9.1%), 1 | 0 (0%), 0 | 0 (0%), 0 |
|  | Blood and lymphatic system disorders - Other, specify: Not clinically significant creatinine kinase, 286, (normal range 30-200) | 1 (9.1%), 1 | 0 (0%), 0 | 0 (0%), 0 |
|  | Blood and lymphatic system disorders - Other, specify: Not clinically significant monocyte count, 0.9, (normal range 0.2 - 0.8) | 1 (9.1%), 1 | 0 (0%), 0 | 0 (0%), 0 |
|  | Blood and lymphatic system disorders - Other, specify: Not clinically significant raised red cell distribution, 14.8 (normal range 11-14) | 1 (9.1%), 1 | 0 (0%), 0 | 0 (0%), 0 |
| Eye disorders | Eye disorders - Other, specify: 'Sticky' left eye | 1 (9.1%), 1 | 0 (0%), 0 | 0 (0%), 0 |
| Gastrointestinal disorders | Constipation | 0 (0%), 0 | 1 (9.1%), 1 | 0 (0%), 0 |
|  | Diarrhoea | 1 (9.1%), 1 | 0 (0%), 0 | 0 (0%), 0 |
|  | Gastrointestinal disorders - Other, specify: Rectal bleeding | 1 (9.1%), 1 | 0 (0%), 0 | 0 (0%), 0 |
| General disorders and administration site conditions | General disorders and administration site conditions - Other, specify: Chesty cough | 1 (9.1%), 1 | 0 (0%), 0 | 0 (0%), 0 |
|  | General disorders and administration site conditions - Other, specify: Cold chills down left arm | 1 (9.1%), 1 | 0 (0%), 0 | 0 (0%), 0 |
|  | General disorders and administration site conditions - Other, specify: Cramps in the chest and abdomen | 1 (9.1%), 1 | 0 (0%), 0 | 0 (0%), 0 |
|  | General disorders and administration site conditions - Other, specify: Discomfort at injection site when touched | 1 (9.1%), 1 | 0 (0%), 0 | 0 (0%), 0 |
|  | General disorders and administration site conditions - Other, specify: Patient felt faint | 1 (9.1%), 1 | 0 (0%), 0 | 0 (0%), 0 |
| Infections and infestations | Infections and infestations - Other, specify: Bilateral ear infection | 1 (9.1%), 1 | 0 (0%), 0 | 0 (0%), 0 |
|  | Infections and infestations - Other, specify: Chest infection | 1 (9.1%), 1 | 0 (0%), 0 | 0 (0%), 0 |
|  | Infections and infestations - Other, specify: Common cold | 3 (27.3%), 4 | 0 (0%), 0 | 0 (0%), 0 |
|  | Infections and infestations - Other, specify: Patient visited the dentist and had a filling. | 1 (9.1%), 1 | 0 (0%), 0 | 0 (0%), 0 |
| Injury, poisoning and procedural complications | Bruising | 9 (81.8%), 92 | 0 (0%), 0 | 0 (0%), 0 |
|  | Fall | 5 (45.5%), 9 | 0 (0%), 0 | 0 (0%), 0 |
|  | Injury, poisoning and procedural complications - Other, specify: Bruising to right hip from fall | 1 (9.1%), 1 | 0 (0%), 0 | 0 (0%), 0 |
|  | Injury, poisoning and procedural complications - Other, specify: Bruising to right shoulder following fall. | 1 (9.1%), 1 | 0 (0%), 0 | 0 (0%), 0 |
|  | Injury, poisoning and procedural complications - Other, specify: Bruising to the face following fall. | 1 (9.1%), 1 | 0 (0%), 0 | 0 (0%), 0 |
|  | Injury, poisoning and procedural complications - Other, specify: Fall up the stairs leading to flat, due to increasing leg weakness | 1 (9.1%), 1 | 0 (0%), 0 | 0 (0%), 0 |
|  | Injury, poisoning and procedural complications - Other, specify: Fall while getting out of bed, due to increase in leg weakness | 1 (9.1%), 1 | 0 (0%), 0 | 0 (0%), 0 |
| Investigations | Activated partial thromboplastin time prolonged | 1 (9.1%), 1 | 0 (0%), 0 | 0 (0%), 0 |
|  | Alanine aminotransferase increased | 2 (18.2%), 3 | 0 (0%), 0 | 0 (0%), 0 |
|  | Alkaline phosphatase increased | 2 (18.2%), 2 | 0 (0%), 0 | 0 (0%), 0 |
|  | Aspartate aminotransferase increased | 3 (27.3%), 6 | 0 (0%), 0 | 0 (0%), 0 |
|  | Blood bilirubin increased | 1 (9.1%), 4 | 0 (0%), 0 | 0 (0%), 0 |
|  | Cholesterol high | 5 (45.5%), 8 | 0 (0%), 0 | 0 (0%), 0 |
|  | Haemoglobin increased | 1 (9.1%), 1 | 0 (0%), 0 | 0 (0%), 0 |
|  | Investigations - Other, specify: Abnormal crp blood results | 1 (9.1%), 1 | 0 (0%), 0 | 0 (0%), 0 |
|  | Investigations - Other, specify: Abnormal eosinophil blood results | 1 (9.1%), 1 | 0 (0%), 0 | 0 (0%), 0 |
|  | Investigations - Other, specify: Abnormal glucose levels (3.1, normal results 3.5-11.0) | 1 (9.1%), 1 | 0 (0%), 0 | 0 (0%), 0 |
|  | Investigations - Other, specify: Abnormal hdl level, clinically insignificant. | 1 (9.1%), 1 | 0 (0%), 0 | 0 (0%), 0 |
|  | Investigations - Other, specify: Abnormal mean cell haemoglobin level, clinically insignificant. | 1 (9.1%), 1 | 0 (0%), 0 | 0 (0%), 0 |
|  | Investigations - Other, specify: Abnormal platelet distribution width result | 1 (9.1%), 1 | 0 (0%), 0 | 0 (0%), 0 |
|  | Investigations - Other, specify: Abnormal ptt blood result (elevated) | 1 (9.1%), 1 | 0 (0%), 0 | 0 (0%), 0 |
|  | Investigations - Other, specify: Abnormal red cell distribution levels | 1 (9.1%), 1 | 0 (0%), 0 | 0 (0%), 0 |
|  | Investigations - Other, specify: Abnormal red cell distribution levels. | 1 (9.1%), 1 | 0 (0%), 0 | 0 (0%), 0 |
|  | Investigations - Other, specify: Creatinine level decreased | 1 (9.1%), 1 | 0 (0%), 0 | 0 (0%), 0 |
|  | Investigations - Other, specify: Crp increased | 1 (9.1%), 1 | 0 (0%), 0 | 0 (0%), 0 |
|  | Investigations - Other, specify: Decreased aptt result | 1 (9.1%), 1 | 0 (0%), 0 | 0 (0%), 0 |
|  | Investigations - Other, specify: Decreased basophil count | 1 (9.1%), 1 | 0 (0%), 0 | 0 (0%), 0 |
|  | Investigations - Other, specify: Decreased basophil level | 2 (18.2%), 2 | 0 (0%), 0 | 0 (0%), 0 |
|  | Investigations - Other, specify: Decreased basophil result | 4 (36.4%), 4 | 0 (0%), 0 | 0 (0%), 0 |
|  | Investigations - Other, specify: Decreased creatinine | 1 (9.1%), 1 | 0 (0%), 0 | 0 (0%), 0 |
|  | Investigations - Other, specify: Decreased creatinine levels | 1 (9.1%), 2 | 0 (0%), 0 | 0 (0%), 0 |
|  | Investigations - Other, specify: Decreased creatinine result | 1 (9.1%), 1 | 0 (0%), 0 | 0 (0%), 0 |
|  | Investigations - Other, specify: Decreased creatinine value - not clinically significant | 1 (9.1%), 1 | 0 (0%), 0 | 0 (0%), 0 |
|  | Investigations - Other, specify: Decreased haematocrit value - not clinically significant | 1 (9.1%), 1 | 0 (0%), 0 | 0 (0%), 0 |
|  | Investigations - Other, specify: Decreased haemoglobin value | 1 (9.1%), 1 | 0 (0%), 0 | 0 (0%), 0 |
|  | Investigations - Other, specify: Decreased hdl cholesterol result | 2 (18.2%), 2 | 0 (0%), 0 | 0 (0%), 0 |
|  | Investigations - Other, specify: Decreased hdl cholesterol value - not clinically significant | 1 (9.1%), 1 | 0 (0%), 0 | 0 (0%), 0 |
|  | Investigations - Other, specify: Decreased hdl cholesterol value -not clinically significant | 1 (9.1%), 1 | 0 (0%), 0 | 0 (0%), 0 |
|  | Investigations - Other, specify: Decreased hdl value - not clinically significant | 1 (9.1%), 1 | 0 (0%), 0 | 0 (0%), 0 |
|  | Investigations - Other, specify: Decreased igm result | 1 (9.1%), 1 | 0 (0%), 0 | 0 (0%), 0 |
|  | Investigations - Other, specify: Decreased monocyte result | 1 (9.1%), 1 | 0 (0%), 0 | 0 (0%), 0 |
|  | Investigations - Other, specify: Decreased potassium level | 1 (9.1%), 1 | 0 (0%), 0 | 0 (0%), 0 |
|  | Investigations - Other, specify: Decreased rbc dist width | 1 (9.1%), 1 | 0 (0%), 0 | 0 (0%), 0 |
|  | Investigations - Other, specify: Decreased rbc distribution width | 2 (18.2%), 2 | 0 (0%), 0 | 0 (0%), 0 |
|  | Investigations - Other, specify: Decreased red blood cell value - not clinically significant | 1 (9.1%), 1 | 0 (0%), 0 | 0 (0%), 0 |
|  | Investigations - Other, specify: Decreased sodium levels | 1 (9.1%), 1 | 0 (0%), 0 | 0 (0%), 0 |
|  | Investigations - Other, specify: Elevated aptt ratio | 1 (9.1%), 2 | 0 (0%), 0 | 0 (0%), 0 |
|  | Investigations - Other, specify: Elevated ast blood level | 1 (9.1%), 1 | 0 (0%), 0 | 0 (0%), 0 |
|  | Investigations - Other, specify: Elevated c- reactive protein blood results | 1 (9.1%), 1 | 0 (0%), 0 | 0 (0%), 0 |
|  | Investigations - Other, specify: Elevated calcim level | 1 (9.1%), 1 | 0 (0%), 0 | 0 (0%), 0 |
|  | Investigations - Other, specify: Elevated calcium level | 1 (9.1%), 1 | 0 (0%), 0 | 0 (0%), 0 |
|  | Investigations - Other, specify: Elevated ck levels | 1 (9.1%), 1 | 0 (0%), 0 | 0 (0%), 0 |
|  | Investigations - Other, specify: Elevated creatine kinase result | 1 (9.1%), 1 | 0 (0%), 0 | 0 (0%), 0 |
|  | Investigations - Other, specify: Elevated crp result | 1 (9.1%), 1 | 0 (0%), 0 | 0 (0%), 0 |
|  | Investigations - Other, specify: Elevated crp value - not clinically significant | 1 (9.1%), 1 | 0 (0%), 0 | 0 (0%), 0 |
|  | Investigations - Other, specify: Elevated eosinophil result | 1 (9.1%), 1 | 0 (0%), 0 | 0 (0%), 0 |
|  | Investigations - Other, specify: Elevated haematocrit blood level | 1 (9.1%), 1 | 0 (0%), 0 | 0 (0%), 0 |
|  | Investigations - Other, specify: Elevated hdl cholesterol blood levels. | 1 (9.1%), 1 | 0 (0%), 0 | 0 (0%), 0 |
|  | Investigations - Other, specify: Elevated mean cell haemoglobin | 1 (9.1%), 1 | 0 (0%), 0 | 0 (0%), 0 |
|  | Investigations - Other, specify: Elevated mean cell hb concentration | 1 (9.1%), 1 | 0 (0%), 0 | 0 (0%), 0 |
|  | Investigations - Other, specify: Elevated mean cell hb level | 1 (9.1%), 1 | 0 (0%), 0 | 0 (0%), 0 |
|  | Investigations - Other, specify: Elevated mean cell hb result | 2 (18.2%), 2 | 0 (0%), 0 | 0 (0%), 0 |
|  | Investigations - Other, specify: Elevated monocyte result | 1 (9.1%), 1 | 0 (0%), 0 | 0 (0%), 0 |
|  | Investigations - Other, specify: Elevated red blood cell count | 3 (27.3%), 4 | 0 (0%), 0 | 0 (0%), 0 |
|  | Investigations - Other, specify: Elevated sodium levels | 1 (9.1%), 1 | 0 (0%), 0 | 0 (0%), 0 |
|  | Investigations - Other, specify: Elevated total protein result | 1 (9.1%), 1 | 0 (0%), 0 | 0 (0%), 0 |
|  | Investigations - Other, specify: Hdl cholesterol levels high | 1 (9.1%), 1 | 0 (0%), 0 | 0 (0%), 0 |
|  | Investigations - Other, specify: Increased ck value - not clinically significant | 1 (9.1%), 1 | 0 (0%), 0 | 0 (0%), 0 |
|  | Investigations - Other, specify: Increased crp level | 1 (9.1%), 1 | 0 (0%), 0 | 0 (0%), 0 |
|  | Investigations - Other, specify: Increased eosinophils count - not clinically significant | 1 (9.1%), 1 | 0 (0%), 0 | 0 (0%), 0 |
|  | Investigations - Other, specify: Increased esr value - not clinically significant | 1 (9.1%), 1 | 0 (0%), 0 | 0 (0%), 0 |
|  | Investigations - Other, specify: Increased iga value - not clinically significant | 1 (9.1%), 1 | 0 (0%), 0 | 0 (0%), 0 |
|  | Investigations - Other, specify: Increased monocyte count - not clinically significant | 1 (9.1%), 1 | 0 (0%), 0 | 0 (0%), 0 |
|  | Investigations - Other, specify: Increased neutrophil count - not clinically significant | 1 (9.1%), 2 | 0 (0%), 0 | 0 (0%), 0 |
|  | Investigations - Other, specify: Increased platelet dist width | 1 (9.1%), 1 | 0 (0%), 0 | 0 (0%), 0 |
|  | Investigations - Other, specify: Increased platelet dist. width | 1 (9.1%), 1 | 0 (0%), 0 | 0 (0%), 0 |
|  | Investigations - Other, specify: Increased rbc distribution width | 1 (9.1%), 1 | 0 (0%), 0 | 0 (0%), 0 |
|  | Investigations - Other, specify: Increased white blood cell value - not clinically significant | 1 (9.1%), 1 | 0 (0%), 0 | 0 (0%), 0 |
|  | Investigations - Other, specify: Low albumin level | 1 (9.1%), 1 | 0 (0%), 0 | 0 (0%), 0 |
|  | Investigations - Other, specify: Low haematocrit | 1 (9.1%), 1 | 0 (0%), 0 | 0 (0%), 0 |
|  | Investigations - Other, specify: Low sodium | 1 (9.1%), 1 | 0 (0%), 0 | 0 (0%), 0 |
|  | Investigations - Other, specify: Mean cell hb concentration level elevated | 1 (9.1%), 1 | 0 (0%), 0 | 0 (0%), 0 |
|  | Investigations - Other, specify: Prolonged pr interval on ecg | 1 (9.1%), 1 | 0 (0%), 0 | 0 (0%), 0 |
|  | Investigations - Other, specify: Raised ast blood levels | 1 (9.1%), 2 | 0 (0%), 0 | 0 (0%), 0 |
|  | Investigations - Other, specify: Raised ast levels | 1 (9.1%), 1 | 0 (0%), 0 | 0 (0%), 0 |
|  | Investigations - Other, specify: Raised blood glucose level | 1 (9.1%), 1 | 0 (0%), 0 | 0 (0%), 0 |
|  | Investigations - Other, specify: Raised crp result | 1 (9.1%), 1 | 0 (0%), 0 | 0 (0%), 0 |
|  | Investigations - Other, specify: Raised eosinophil count | 1 (9.1%), 1 | 0 (0%), 0 | 0 (0%), 0 |
|  | Investigations - Other, specify: Raised eosinophils | 1 (9.1%), 1 | 0 (0%), 0 | 0 (0%), 0 |
|  | Investigations - Other, specify: Raised hdl cholesterol blood levels. | 1 (9.1%), 1 | 0 (0%), 0 | 0 (0%), 0 |
|  | Investigations - Other, specify: Raised sodium level | 1 (9.1%), 1 | 0 (0%), 0 | 0 (0%), 0 |
|  | Investigations - Other, specify: Raised total protein result | 1 (9.1%), 1 | 0 (0%), 0 | 0 (0%), 0 |
|  | Investigations - Other, specify: Reduced aptt ratio value - not clinically significant | 1 (9.1%), 1 | 0 (0%), 0 | 0 (0%), 0 |
|  | Investigations - Other, specify: Reduced basophil count | 1 (9.1%), 1 | 0 (0%), 0 | 0 (0%), 0 |
|  | Investigations - Other, specify: Reduced free thyroxine levels | 1 (9.1%), 1 | 0 (0%), 0 | 0 (0%), 0 |
|  | Lymphocyte count decreased | 1 (9.1%), 1 | 0 (0%), 0 | 0 (0%), 0 |
|  | Neutrophil count decreased | 2 (18.2%), 3 | 0 (0%), 0 | 0 (0%), 0 |
|  | White blood cell decreased | 3 (27.3%), 5 | 0 (0%), 0 | 0 (0%), 0 |
| Musculoskeletal and connective tissue disorders | Back pain | 1 (9.1%), 1 | 0 (0%), 0 | 0 (0%), 0 |
|  | Musculoskeletal and connective tissue disorder - Other, specify: Fractured nasal bone following fall. | 1 (9.1%), 1 | 0 (0%), 0 | 0 (0%), 0 |
|  | Musculoskeletal and connective tissue disorder - Other, specify: Increased leg weakness leading to inability to stand resulting in admission to hospital | 0 (0%), 0 | 0 (0%), 0 | 1 (9.1%), 1 |
|  | Musculoskeletal and connective tissue disorder - Other, specify: Muscle spasm in legs | 1 (9.1%), 1 | 0 (0%), 0 | 0 (0%), 0 |
|  | Musculoskeletal and connective tissue disorder - Other, specify: Occasional spasm in neck when yawning | 1 (9.1%), 1 | 0 (0%), 0 | 0 (0%), 0 |
|  | Musculoskeletal and connective tissue disorder - Other, specify: Painful left shoulder blade. | 0 (0%), 0 | 1 (9.1%), 1 | 0 (0%), 0 |
|  | Musculoskeletal and connective tissue disorder - Other, specify: Right hip pain | 0 (0%), 0 | 1 (9.1%), 1 | 0 (0%), 0 |
| Nervous system disorders | Headache | 1 (9.1%), 1 | 0 (0%), 0 | 0 (0%), 0 |
| Psychiatric disorders | Insomnia | 1 (9.1%), 1 | 0 (0%), 0 | 0 (0%), 0 |
| Respiratory, thoracic and mediastinal disorders | Allergic rhinitis | 1 (9.1%), 1 | 0 (0%), 0 | 0 (0%), 0 |
|  | Respiratory, thoracic and mediastinal disorders - Other, specify: Pneumonia | 1 (9.1%), 1 | 0 (0%), 0 | 0 (0%), 0 |
|  | Sore throat | 1 (9.1%), 1 | 0 (0%), 0 | 0 (0%), 0 |
| Skin and subcutaneous tissue disorders | Skin and subcutaneous tissue disorders - Other, specify: Eczema | 0 (0%), 0 | 1 (9.1%), 1 | 0 (0%), 0 |
|  | Skin and subcutaneous tissue disorders - Other, specify: Rash on abdomen around injection site | 1 (9.1%), 1 | 0 (0%), 0 | 0 (0%), 0 |
|  | Skin and subcutaneous tissue disorders - Other, specify: Rash on both feet | 1 (9.1%), 1 | 0 (0%), 0 | 0 (0%), 0 |
| **Total Number of Adverse Events** | | **265** | **4** | **1** |
| Total Number of Patients Affected | | 159 | 4 | 1 |

aptt, activated partial thromboplastin time; ast, aspartate aminotransferase; ck, creatine kinase; crp, c-reactive protein; ecg, electrocardiogram; esr, erythrocyte sedimentation rate; hb, haemoglobin; hdl, high-density lipoprotein; iga, immunoglobulin A; igm, immunoglobulin M; ptt, partial thromboplastin time; rbc, red blood cell.

* According to National Cancer Institute (NCI) Common Terminology Criteria for Adverse Events (CTCAE) v4.03 (2010). Available from: <https://ctep.cancer.gov/protocoldevelopment/electronic_applications/ctc.htm#ctc_40>. where grades 1-to-3 are defined as:

- Grade 1 – mild; asymptomatic or mild symptoms; clinical or diagnostic observations only; intervention not indicated.
- Grade 2 – moderate; minimal, local, or non-invasive intervention indicated; limiting age-appropriate instrumental activities of daily life.
- Grade 3 – severe or medically significant but not immediately life-threatening; hospitalisation or prolongation of hospitalisation indicated; disabling; limiting self-care activities of daily life
